# Prompt engineering in consistency and reliability with the evidence-based guideline for LLMs

**DOI:** 10.1038/s41746-024-01029-4

**Published:** 2024-02-20

**Authors:** Li Wang, Xi Chen, XiangWen Deng, Hao Wen, MingKe You, WeiZhi Liu, Qi Li, Jian Li

**Affiliations:** 1 Sports Medicine Center, West China Hospital, Sichuan University, Chengdu, China; 2 Department of Orthopedics and Orthopedic Research Institute, West China Hospital, Sichuan University, Chengdu, China; 3 Shenzhen International Graduate School, Tsinghua University, Beijing, China

**Keywords:** Preclinical research, Mathematics and computing

## Abstract

The use of large language models (LLMs) in clinical medicine is currently thriving. Effectively transferring LLMs’ pertinent theoretical knowledge from computer science to their application in clinical medicine is crucial. Prompt engineering has shown potential as an effective method in this regard. To explore the application of prompt engineering in LLMs and to examine the reliability of LLMs, different styles of prompts were designed and used to ask different LLMs about their agreement with the American Academy of Orthopedic Surgeons (AAOS) osteoarthritis (OA) evidence-based guidelines. Each question was asked 5 times. We compared the consistency of the findings with guidelines across different evidence levels for different prompts and assessed the reliability of different prompts by asking the same question 5 times. gpt-4-Web with ROT prompting had the highest overall consistency (62.9%) and a significant performance for strong recommendations, with a total consistency of 77.5%. The reliability of the different LLMs for different prompts was not stable (Fleiss kappa ranged from −0.002 to 0.984). This study revealed that different prompts had variable effects across various models, and the gpt-4-Web with ROT prompt was the most consistent. An appropriate prompt could improve the accuracy of responses to professional medical questions.

## Introduction

Large language models (LLMs) have shown good performance in various natural language processing (NLP) tasks, such as summarizing, translating, code synthesis, and even logical reasoning^1–3^. There is growing interest in exploring the potential of LLMs in medicine. They have been used in related medical studies in case diagnoses, medical examinations, and guideline consistency assessments^4–7^.

However, the current performance of LLMs in the medical field is not perfect. In the diagnosis of complex cases, 39% of the GPT-4-related diagnoses were consistent with the final diagnosis, and an average consistency of 60% was shown with the guidelines for digestive system diseases^4,6^. Eighteen percent of the Med-PaLM-related answers were judged to contain inappropriate or incorrect content^8^. Moreover, LLMs may generate different answers to the same question, and self-consistency has always been a crucial parameter for assessing the performance of LLMs^9,10^. Further research and exploration on how to optimize its performance in the medical field are necessary^1,4,6,8^.

Prompt engineering is a new discipline that focuses on the development and optimization of prompt words, thereby helping users apply LLMs to various scenarios and research fields. In computer science, LLMs can obtain ideal and stable answers through prompt engineering, and adopting different prompts will affect the performance of LLMs, which is somewhat reflected in mathematical problems^9,11–13^. The newly used prompt designs currently include chain of thoughts (COT) prompting and tree of thoughts (TOT) prompting^12,13^. With the proposal of the COT and TOT theories in the computer science LLM field, corresponding prompts have been developed and exhibited improved performance in mathematical problems^12,13^.

In clinical medicine, a few studies have shown the application of prompts such as COT prompting, few-shot prompting and self-consistency prompting in the study of Karan et al. ^8^. In addition, the study of Bertalan et al. ^14^. Summarizes the current state of research on prompt engineering and provides a tutorial for prompt engineering for medical professionals. Overall, few studies have focused on the differences in the performance of different prompts in medical questions or examined whether there is a need to develop prompts specifically for medical questions. In summary, the application of LLMs in medicine is currently thriving. However, most of the current research seems to focus more on the results of using LLMs rather than how to better use LLMs in clinical medicine. Testing the reliability of LLMs in answering medical questions, using different prompts, and even developing prompts specifically for medical questions could change the application of LLMs in medicine and future research. It is important to investigate whether and how prompt engineering may improve the performance of LLMs in answering medical-related questions. Additionally, other factors, such as the type of model architecture, model parameters, training data, and fine-tuning techniques, can influence the performance of LLMs^15–17^.

To explore the influence of different types of prompts combined with other factors on the performance of LLMs, we conducted a pilot study on osteoarthritis (OA)-related questions. The 2019 Global Burden of Disease tool identified OA as one of the most prevalent and debilitating diseases^18^. In terms of prevalence and impact, OA is one of the most prevalent musculoskeletal disorders and affects a substantial portion of the global population, especially elderly individuals^19^. This widespread impact makes it a public health concern of significant importance, and the management of OA is complex and multifaceted, encompassing pain control, physical therapy, lifestyle modifications, and, in some cases, surgical interventions^20^. Given that it is a common disease with a large patient population and complex management, patients and doctors may seek relevant professional knowledge online, which includes LLMs. Therefore, investigating the performance of LLMs with respect to OA-related questions could serve as an appropriate example of how to improve answer quality through prompt engineering. The potential of prompt engineering to assist both doctors and patients in medical queries of common diseases could also be explored by using LLMs.

Our research applied the same set of prompts to different LLMs, asking OA-related questions and aiming to explore the effectiveness of prompt engineering. We hypothesized that different prompts would result in different consistency and reliability and that the effectiveness of prompts on LLMs would be influenced by various factors.

## Results

### Consistency

**Fig. 1:**
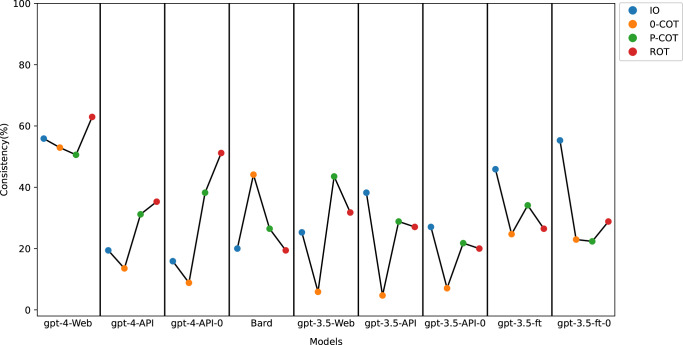
Consistency of different prompts in different models. Detailed information of each model could be found in Table 3.

The results indicated that gpt-4-Web outperformed the other models, as shown in Fig. 1. The consistency rates for the four prompts in gpt-4-Web ranged between 50.6% and 63%. Other consistency rates were also observed with IO prompting in the gpt-3.5-ft-0 at 55.3% and ROT prompting in gpt-4-API-0 at 51.2%. The consistency rates for the other models were all less than 50% (4.7% to 45.9%).

**Fig. 2:**
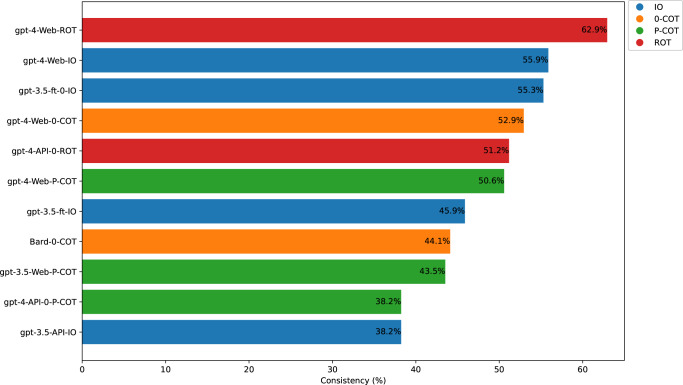
Top 10 consistency. The vertical axis represents the combination of the chosen model and prompt, for example, ‘gpt-4-Web-ROT’ indicates that the selected model is gpt-4-Web, and the prompt is ROT prompting.

The combination of gpt-4-Web and ROT generated the treatment recommendation most adherent to the clinical guidelines. The top 10 combinations of prompts and models are shown in Fig. 2. Specifically, the consistency of different prompts with the guidelines in a series of GPT-4 models ranged from 8.8% to 62.9%; in a series of GPT-3.5 models, including fine-tuned versions, it ranged from 4.7% to 55.3%. For different prompts in Bard, the consistency ranged from 19.4% to 44.1%. For the three versions of the GPT-4, the ROT prompting was consistently the best prompt, ranging from 35.3% to 63%. For five versions of the GPT-3.5, except for P-COT prompting, which was the best prompt for gpt-3.5-Web at 43.5%, the best prompt for the other versions was IO prompting (ranging from 27.1% to 55.3%). For Bard, the best prompt was 0-COT prompting at 44.1%.

### Subgroup analysis

The AAOS categorizes recommendation levels based on the strength of supporting evidence, ranging from strong to moderate, limited, and consensus. We hypothesized that different levels of evidence strength might lead to variations in consistency. To explore this, we conducted a subgroup analysis to examine the performance differences of various prompts across different evidence strength levels. Within the same model, we conducted multiple comparisons between different prompts, with a focus on the performance of the outperformed gpt-4-Web across various evidence strengths. The results of the subgroup analysis and the multiple comparisons within each model can be found in Supplementary Table 1.

#### Strong level

**Fig. 3:**
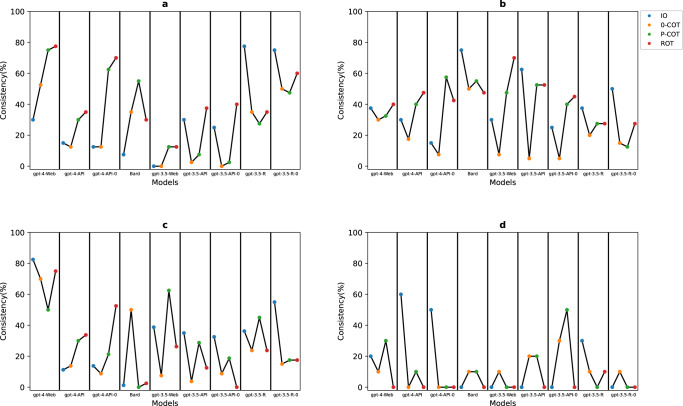
Consistency in different levels. **a** Strong; **b** moderate; **c** limited; **d** consensus.

The consistency of the different prompts in the different models at the strong level is shown in Fig. 3a. Eight pieces of advice are rated as strong by the AAOS guidelines, with 40 responses for each prompt. According to the multiple comparisons of consistency in gpt-4-Web, the percentage differences in the ROT prompting (77.5%) and P-COT prompting (75%) scores were significantly greater than that in the IO prompting (30%). According to the other models, the consistency of the IO prompting at gpt-3.5-ft and gpt-3.5-ft-0 was 77.5% and 75%, respectively.

#### Moderate level

The consistency of the different prompts in the different models at the moderate level is shown in Fig. 3b. Eight pieces of advice were rated as moderate, with 40 responses for each prompt. According to the multiple comparisons of consistency in gpt-4-Web (30% to 40%), there was no significant difference between the groups. According to the other models, the consistency of the IO prompting in Bards is 75%.

#### Limited level

The consistency of the different prompts in the different models at the limited level is shown in Fig. 3c. Sixteen pieces of advice had a limited recommendation rating, with 80 responses for each prompt. According to the multiple comparisons of consistency in gpt-4-Web, after Bonferroni correction, the percentage of patients with a 0-point difference in P-COT prompting (50%) was significantly lower than that in ROT prompting (75%) and IO prompting (82.5%). In the other models, all the consistency is lower than 70%.

#### Consensus level

The two pieces of advice were recommended upon consensus. Considering the small sample size, no statistical test was conducted, and the consistency of different prompts in different models is shown in Fig. 3d.

### Reliability of LLMs

The Fleiss kappa values of the 4 prompts in the 9 models are shown in Table 1. and the values ranged from −0.002 to 0.984. Detailed statistical data are shown in Supplementary Table 2.

**Table 1:** Fleiss Kappa of different prompts in different models

Model	Prompt	Fleiss Kappa	95% CI	
gpt-4-Web	IO	0.525	0.523	0.527
	0-COT	0.450	0.448	0.452
	P-COT	0.334	0.332	0.337
	ROT	0.467	0.465	0.470
gpt-4-API	IO	0.288	0.286	0.290
	0-COT	0.067	0.065	0.069
	P-COT	0.331	0.330	0.333
	ROT	0.205	0.203	0.206
gpt-4-API-0	IO	0.525	0.523	0.526
	0-COT	0.285	0.283	0.287
	P-COT	0.660	0.658	0.661
	ROT	0.451	0.449	0.453
Bard	IO	0.374	0.372	0.376
	0-COT	0.355	0.353	0.357
	P-COT	0.323	0.321	0.326
	ROT	0.180	0.178	0.182
gpt-3.5-Web	IO	0.409	0.407	0.411
	0-COT	−0.002	−0.004	0.000
	P-COT	0.276	0.274	0.278
	ROT	0.016	0.014	0.018
gpt-3.5-API	IO	0.188	0.186	0.190
	0-COT	0.004	0.002	0.006
	P-COT	0.031	0.029	0.033
	ROT	0.014	0.012	0.016
gpt-3.5-API-0	IO	0.984	0.983	0.986
	0-COT	0.461	0.459	0.464
	P-COT	0.533	0.531	0.535
	ROT	0.581	0.578	0.583
gpt-3.5-ft	IO	0.162	0.160	0.164
	0-COT	0.021	0.020	0.023
	P-COT	0.065	0.063	0.067
	ROT	0.033	0.032	0.035
gpt-3.5-ft-0	IO	0.982	0.980	0.984
	0-COT	0.412	0.410	0.414
	P-COT	0.355	0.353	0.356
	ROT	0.398	0.396	0.400

**Fig. 4:**
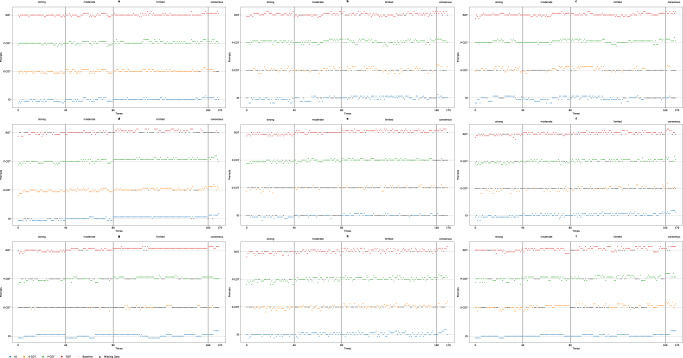
Scatter plots of each answer. **a** gpt-4-Web; **b** gpt-4-API; **c** gpt-4-API-0; **d** Bard; **e** gpt-3.5-Web; **f** gpt-3.5-API; **g** gpt-3.5-API-0; **h** gpt-3.5-ft; **i** gpt-3.5-ft-0.

The kappa values for IO prompting in gpt-3.5-ft-0 and gpt-3.5-API-0 were nearly 1 (0.982 and 0.984, respectively). In the corresponding scatter plots, as shown in Fig. 4g, i, points that match the answers with the guidelines fall on the baseline (level difference = 0). A positive difference indicates being above the baseline, while a negative difference indicates being below it. Starting from the first data point of IO prompting in Fig. 4g, i shows that almost every set of five points lies on a horizontal line. This pattern indicates that the models consistently generate the same response five times in a row. In contrast, the responses in other circumstances exhibit more variability. The kappa of P-COT prompting in response to the gpt-4-API-0 was 0.660. The other kappa values are all lower than 0.6. For the gpt-4-Web, the Fleiss kappa results indicate that the reliability of each prompt is fair to moderate (0.334 to 0.525). Overall, IO prompting in the gpt-3.5-ft-0 and gpt-3.5-API-0 trials demonstrated perfect reliability. P-COT prompting in the gpt-4-API-0 indicated substantial reliability, and others were moderate or lower.

### Invalid data and corresponding processing measures

There were three categories of invalid data: Category A: the final rating was not provided. Category B: the rating was not an integer. All the invalid data were processed according to the invalid data procedure^21^. In the calculation of Fleiss kappa, all invalid data in category A are considered to constitute an independent classification, and the invalid data in category B are treated as different classifications based on the values (if the rating is ‘2 or 3’, it is recorded as 2.5) generated by the LLMs. In the creation of the scatter plot (Fig. 4), invalid data from category A were labeled missing data. Notably, a significant amount of invalid data from category A was observed in multiple datasets; for instance, 81.1% of the responses to 0-COT prompting were recorded in gpt-3.5-API-0. Conversely, the proportion of invalid data in gpt-4-Web was relatively small (a total of 14 out of 680 across all four prompts).

## Discussion

The results of this study suggested that prompt engineering may change the accuracy of LLMs in answering medical questions. Additionally, LLMs do not always provide the same answer to the same medical questions. The combination of ROT prompting and gpt-4-Web outperformed the other combinations in providing professional OA knowledge consistent with clinical guidelines.

We have summarized the current performance of LLMs in diagnosing patients, querying patients, and examining patients within clinical medicine in Supplementary Table 3. Indeed, GPT-4 has shown superior results and exhibited superior performance compared to both GPT-3.5 and Bard in the field of clinical medicine^16,22–29^. In our study, by combining the performance of the four types of prompts across different models, as shown in Fig. 1, gpt-4-Web, also known as ChatGPT-4, demonstrated a more balanced and prominent performance.

Previous research has primarily assessed GPT-4 through web interfaces in clinical medicine. The study of Fares et al. ^30^ accessed GPT-4 via the API and set different temperatures (temperature = 0, 0.3, 0.7, 1) and found that the model set at a temperature of 0.3 performed better in answering ophthalmology-related questions. Our study revealed differences in consistency and reliability between GPT-4 scores accessed via the web and GPT-4 scores accessed through the API. In our study, we found that among the gpt-4-Web products with specific parameter settings, gpt-4-API with a temperature of 0 (gpt-4-API-0) and gpt-4-API with a temperature of 1, gpt-4-Web exhibited the most prominent performance. This indicated that adjusting the internal parameters of LLMs during different tasks can alter the performance of LLMs.

To our knowledge, there has not yet been research exploring the impact of fine-tuning ChatGPT on clinical medicine. For other LLMs, in the study by Karan et al. ^8^, Med-PaLM, which is a version of Flan-PaLM that has been instruction prompt-tuned and is not currently publicly available, was evaluated by a panel of clinicians. They found that 92.6% of the answers generated by Med-PaLM were consistent with the scientific consensus. For our study, in the fine-tuning versions of GPT-3.5, where IO prompting is used as the input part of the dataset during fine-tuning, the 2 fine-tuning models achieve consistencies of 55.3% and 45.9% when IO prompting is used for inputs. However, when other types of prompts are used as inputs in the fine-tuning models, the performance deteriorates (22.4% to 34.1%). Furthermore, fine-tuning could not ensure that GPT-3.5 fully understood the rationale behind each piece of advice in the dataset. As a result, answers can be generated with incorrect rationales. The less-than-ideal fine-tuning results in our study might be due to the setup of the fine-tuning dataset, the capability of the base model or the fine-tuning methods employed by OpenAI.

Overall, the comparison of nine LLMs indicates that parameter settings and fine-tuning, along with prompt engineering, could influence the performance of LLMs. Improving LLMs in clinical medicine requires a combination of multiple approaches, accounting for various factors, including model architecture, parameter settings, and fine-tuning techniques.

Supplementary Table 4 briefly summarizes the current application of different types of prompts in clinical medicine. Studies on the topic of prompt engineering in clinical medicine are limited, and most studies primarily apply prompt engineering techniques directly^31^ or provide an overview of prompt engineering^14,32,33^ in clinical medicine. The study of Karan et al. ^8^ did not significantly differ between the COT and few-shot prompting strategies. However, self-consistency prompting, particularly in the context of the MedQA dataset, showed an improvement of more than 7%. Conversely, self-consistency led to a decrease in performance for the PubMedQA dataset. Wan et al. ^31^ demonstrated that few-shot prompting and zero-shot prompting exhibit different levels of sensitivity and specificity in converting symptom narratives using the ChatGPT-4.

This study, built upon previous research, further indicated that prompt engineering could influence the performance of LLMs in clinical medicine. Based on current theories of prompt engineering, we developed a new prompting framework, ROT prompting, which demonstrated good performance on the gpt-4-Web. As shown in Fig. 2, ROT prompting achieved the highest consistency rate. According to our subgroup analysis, compared to those of the other three types of prompts within gpt-4-Web, the ROT prompting performed more evenly and prominently. In terms of ‘strong’ intensity, ROT prompting is superior to IO prompting, and it is not significantly inferior to other prompts at other levels. In contrast, although answers of P-COT prompting at ‘strong’ intensity are better than those of IO prompting, its performance at the ‘limited’ intensity level is significantly worse than that of other prompts.

However, ROT promoting is not necessarily the best prompt for other LLMs. For instance, for five versions of GPT-3.5, except for P-COT prompting being the best prompt for GPT-3.5-Web, the best prompt for other versions was IO prompting. For Bard, the best prompt was 0-COT. This indicated that we could try different prompting strategies to obtain the best responses.

The ROT prompting asked LLM to return to previous thoughts and examine if they were appropriate, which may improve the robustness of the answer. Furthermore, the ROT-based design can minimize the occurrence of egregiously incorrect answers from the gpt-4-Web. For instance, regarding a ‘strong’ level suggestion, “Lateral wedge insoles are not recommended for patients with knee osteoarthritis.” ROT prompting provided four ‘strong’ answers and one ‘moderate’ answer in five responses. Initially, in this ‘moderate’ response (Supplementary Note 1), two “experts” provided “limited” answers, and one “expert” answered “moderate”. After “discussion”, all “experts” agreed on a ‘moderate’ recommendation. The final reason was that even though there was high-quality evidence to support the advice, there might still be slight potential benefits for some individuals. Notably, the reasons given by the two experts for “limiting” seem to be more in line with the statement “Lateral wedge insoles are recommended for patients with knee osteoarthritis.” This implies that these two “experts” did not fully understand the medical advice correctly, as “Expert C” mentioned in step five: “Observes that the results are somewhat mixed, but there’s a general agreement that the benefits, if any, from lateral wedge insoles are limited.” However, after the “discussion”, the final revised recommendation and reason were deemed acceptable. Referring to the application of TOT in the 24-point game^13^, the prompt designed in the style of TOT as well as the ROT prompting in this study could offer more possibilities at every step of the task, and LLM could be asked to return to previous thoughts, aiming to induce LLM to generate more accurate answers.

In future studies, considering the varying effectiveness of the ROT prompting across different models, a potential direction might involve optimizing it based on model differences. In the future, the design of the ROT prompting needs to be more closely aligned with different clinical scenarios. For instance, setting up roles with various professional backgrounds in disease diagnosis and treatment could provide more specialized advice. Additionally, incorporating different clinical application scenarios, such as testing and improving the effectiveness of ROT prompting in disease diagnosis and patient treatment plan formulation, will be crucial.

Three previous studies^6,7,34^ briefly described reliability. Yoshiyasu et al. ^7^ reproduced inaccurate responses only. Walker et al. ^6^ reported that the internal concordance of the provided information was complete (100%) according to human evaluation. In the study of Fares et al. ^34^, the authors repeated the experiments 3 times and extracted the responses from ChatGPT-3.5; the κ values were 0.769 for the BCSC set and 0.798 for the OphthoQuestions set.

In this study, reliability was investigated by asking LLMs the same question five times, and according to the results of our study, it is suggested that LLMs cannot always provide consistent answers to the same medical question (Table 1 and Fig. 4). The study used the strength of recommendation of the AAOS as an evaluation standard and found that LLMs always provide different strengths for the same advice in multiple answers. Only IO prompting in gpt-3.5-API-0 and gpt-3.5-ft-0, both of which were set at a temperature of 0, demonstrated perfect reliability.

Based on the description on the official OpenAI website regarding the endpoint of Audio (https://platform.openai.com/docs/api-reference/audio/createTranscription), “The sampling temperature, between 0 and 1, affects randomness. Higher values, such as 0.8, increase randomness, while lower values, such as 0.2, make outputs more focused and deterministic. A setting of 0 allows the model to automatically adjust the temperature based on log probability until certain thresholds are met.” We hypothesize that this mechanism also applies to the endpoint of Chat (https://platform.openai.com/docs/api-reference/chat/object), although this is not explicitly stated in the corresponding section. The specific thresholds for GPT-3.5 and GPT-4 might differ, and the prompts could influence these thresholds, as consistent responses were observed only in the two groups corresponding to the IO prompting in gpt-3.5-API-0 and gpt-3.5-ft-0. Therefore, it is recommended that LLMs be asked the same questions several times to obtain more comprehensive answers and that they keep asking the ChatGPT-4 the same question until it does not provide any additional information.

In future research, within the clinical application of LLMs, particularly from the patient’s perspective, OA is a common and frequently occurring condition associated with various treatment methods. Hence, prompt engineering could play a crucial role in guiding patients to ask medical questions correctly, potentially enhancing patient education and answering their queries more effectively. On the side of doctors, our study demonstrated that the ROT developed for the web version of the gpt-4 generated better results. However, multiple variables, such as different model architectures and parameters, can complicate outcomes. Therefore, we believe that prompt engineering should be combined with model development, parameter adjustment, and fine-tuning techniques to develop specialized LLMs with medical expertise, which could assist physicians in making clinical decisions.

The application of prompt engineering faces several challenges in the future. First, there is the issue of the robustness of prompts. Prompts based on the same framework may yield different answers due to minor changes in a few words^35^. Patients or doctors might receive different answers even when using prompts from the same framework. Second, prompt engineering performance depends on the inherent capabilities of the LLM itself. Prompts effective for one model may not be suitable for another. Guidelines for prompt engineering tailored for patients and doctors need to be developed according to the corresponding requirements. Overall, future related studies should examine the applicability and robustness of prompts and formulate relevant guidelines.

Importantly, our research does not include real-time interactions or validations with healthcare professionals or patients. However, our approach to data collection relies on nonhuman subjective scoring, objectively assessing the consistency and reliability of LLM responses. Furthermore, the study was designed based on expected answers derived from guidelines and lacked prospective validation. Nevertheless, we acknowledge that this field remains underexplored and that a multitude of techniques warrant further investigation. Our study represents only a preliminary foray into this vast domain.

Given these limitations, future research should aim to develop both an objective benchmark evaluation framework for LLM responses and a human evaluation framework^8^ involving healthcare professionals and patients.

Our work represents an initial step into this expansive domain, highlighting the importance of continuing research to refine and enhance the application of large language models in healthcare. Future studies should further explore various methodologies to improve the effectiveness and reliability of LLMs in medical settings.

This study revealed that different prompts had variable effects across various models, and gpt-4-Web with ROT prompting had the highest consistency. An appropriate prompt may improve the accuracy of responses to professional medical questions. Moreover, it is advisable to pose the input questions multiple times to gather more comprehensive insights, as responses may vary with each inquiry. In the future of AI healthcare involving LLMs, prompt engineering will serve as a crucial bridge in communication between LLMs and patients, as well as between LLMs and doctors.

## Method

### Disease selection and evidence-based CPG selection

The American Academy of Orthopedic Surgeons (AAOS) evidence-based clinical practice guidelines (CPGs) for OA were used to test the consistency of the answers given by the LLMs. With more than 39,000 members, the AAOS is the world’s largest medical association of musculoskeletal specialists^36^, and the OA guidelines provided by the AAOS are supported by detailed evidence and review reports^37^. The OA guidelines include a detailed evidence assessment system based on research evidence and cover various management recommendations, including drug treatment for OA, physical therapy, and patient education. It is an authoritative and comprehensive guide with detailed content. More detailed information can be found in the complete version of the OA guidelines^38^.

### Prompt design

Based on the current application of prompting engineering in computer science and the task of this study, four types of prompts were applied for this study: IO prompting, 0-COT prompting, P-COT prompting and ROT prompting. These types of prompts were developed to test the compliance of LLMs’ answers regarding the AAOS guidelines and to assess the reliability of the answers in repeated requests. LLMs were tasked with generating an answer that included the rating score as the final output.

**Fig. 5:**
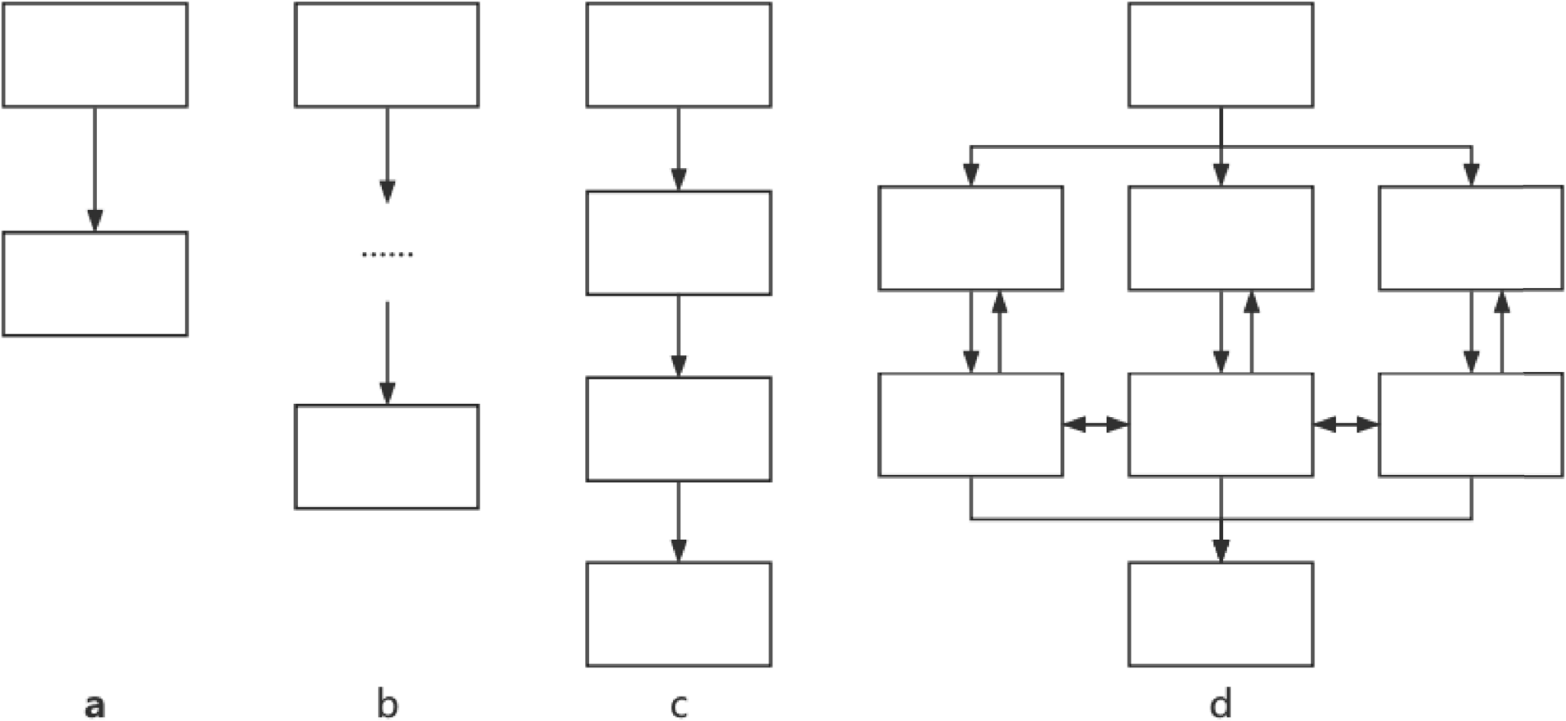
The schematic diagram of four prompt words guiding LLMs to output answers. **a** IO prompting；**b** 0-COT prompting; **c** P-COT prompting; **d** ROT prompting. The design of this figure was inspired by the study of Yao et al. ^13^, and the copyright is authorized under the CC BY 4.0 DEED (https://creativecommons.org/licenses/by/4.0/).

A brief illustration and examples of each prompt type are shown in Fig. 5 and Table 2. For the detailed content of the four prompts, please refer to Supplementary Table 5.

**Table 2:** Definition and explanation of each prompt

Prompt	Definition	Brief explanation
Input-output (IO) prompting	Input the instruction directly	Consider the following medical advice: <insert the advice> Rate the medical advice using the following criteria, and make a selection from integer 1,2,3,4 <insert the criteria>
0-shot-Chain of thought (0-COT) prompting	Use “Think it step by step” on the base of IO to steer the LLM complete reasoning.	<Describe your task> Complete the task above step by step.
Performed-Chain of thought (P-COT) prompting	Break down the task into different steps to perform what reasoning processes need to be conducted by the LLM.	<Describe your task> Complete the task above step by step: Step 1….. Step 2….. …… Show your work of each step.
Reflection of thoughts (ROT) prompting	Break down the task into different steps and steer the LLM to backtrack previous steps by let the LLM simulates the mode of discussion.	<Describe your task> Imagine 3 medical experts are completing the task above step by step: Step 1 to Step X: Each expert independently completes reasoning. After step X: Experts discuss together and backtrack previous steps and finally reach agreement.

### Model setting

We utilized a total of 9 LLMs, the details of which are shown in Table 3. The default web versions of GPT-4, GPT-3.5 and Bard were accessed via web interfaces, while other LLMs were accessed through the Application Programming Interface (API). The fine-tuning and calling of an API were conducted as described in the OpenAI platform. For the fine-tuning data, the IO prompting and the rationale of each advice in AAOS were used to form the fine-tuning data, and all the fine-tuning data can be found in Supplementary Table 6.

**Table 3:** Details of included models

Model name	Version name	Details
GPT-4	gpt-4-Web	The default web version of GPT-4 and the release notes were on July 20, 2023.
	gpt-4-API	gpt-4-0613 with parameters when assessing API (temperature = 1).
	gpt-4-API-0	gpt-4-0613 with setting temperature as 0 when assessing API.
Bard	Bard	Assess through web and the release notes were on October 30, 2023.
GPT-3.5	gpt-3.5-Web	Assess through web and the release notes were on October 17, 2023.
	gpt-3.5-API	gpt-3.5-turbo-0613 with default parameters when assessing API.
	gpt-3.5-API-0	gpt-3.5-turbo-0613 with setting temperature as 0 when assessing API.
	gpt-3.5-ft	gpt-3.5-turbo-0613 with fine-tuning techniques and default parameters (temperature=1) when assessing API.
	gpt-3.5-ft-0	gpt-3.5-ft with setting temperature as 0 when assessing API.

### Data collection and data processing

Each item from the AAOS guidelines was reformatted as an instruction for assessing the strength of the recommendation to different LLMs, and the results showed the level of recommendation. The AAOS’s level of recommendation was based on the level of evidence, and any upgrade or downgrade of the recommendation strength based on evidence to the decision framework requires supermajority approval by the AAOS working group^36^. The answers provided by the LLMs were compared to those of the AAOS guidelines, and each level provided by the LLMs was offset from the corresponding AAOS level, as shown in Table 4.

**Table 4:** An example of calculating level difference

Input	Output
Consider the following medical advice: Dry needling may be used to improve pain and function in patients with knee osteoarthritis. Rate the medical advice using the following criteria, and make a selection from integer 1,2,3,4: Score 1 (Consensus): There is no supporting evidence, and the medical advice is possibly based on common practice or expert opinion. Score 2 (Limited): Supported by evidence from one or more low-quality studies with consistent findings, or evidence from a single moderate-quality study. Score 3 (Moderate): Supported by evidence from two or more moderate-quality studies with consistent findings, or evidence from a single high-quality study. Score 4 (Strong): Supported by evidence from two or more high-quality studies with consistent findings.	As of my knowledge cut-off in September 2021, the use of dry needling for the improvement of pain and function in patients with knee osteoarthritis may be rated as 2 (Limited). The evidence for this practice comes from a few low to moderate-quality studies showing some potential benefits. However, more rigorous, high-quality studies are needed to confirm these findings and establish a solid consensus in the medical community. Please consult with a healthcare professional for updated information or advice as the state of medical knowledge and research is constantly evolving.
	Recommendation level of AAOS: 2
	Level difference: 2-2 = 0

We extracted 34 items (Supplementary Table 7) from the evidenced-based OA CPG provided by the AAOS. Each piece of advice was asked 5 times. When assessing via web interfaces, each question was asked in a separate dialog box to avoid the influence of context on the answers. When assessing the API, the process was completed by means of codes in Python (version 3.9.7). Finally, each prompt was asked a total of 170 times, and the four prompts were asked a total of 680 times for each LLM. The answer to each question was recorded. Answers that did not follow the instructions of the prompt were considered invalid data.

### Outcome measures and statistical analysis

Statistical analysis was conducted using SPSS 23.0 (IBM, New York, NY, USA) and Python (version 3.9.7). Consistency and reliability were used to evaluate the performance of the LLMs. Consistency is defined as the proportion of instances where the level gap equals zero. To compare consistency, we grouped the categorical data collected into a category with a rank difference of 0 and another with a rank difference not equal to 0 and then conducted the chi-square test, Fisher’s exact test, or Yates’s continuity correction^39,40^. Bonferroni correction was used for multiple comparisons^41^. Reliability refers to the repeatability of responses to the same questions and was assessed using the Fleiss kappa test. The values of Fleiss kappa, as interpreted based on previous studies^42,43^, are considered to indicate no reliability (<0.01), slight reliability (0.01–0.2), fair reliability (0.21–0.40), moderate reliability (0.41–0.60), substantial reliability (0.61–0.80), or almost perfect reliability (0.81–1.00). Invalid data were treated according to invalid data procedures in the statistical analysis^21^.

### Reporting summary

Further information on research design is available in the Nature Research Reporting Summary linked to this article.

### Supplementary information

Supplementary File

Reporting Summary

## Data Availability

The original answers for the gpt-4-Web can be found in the supplementary files of the preprint version of this article at https://www.researchsquare.com/article/rs-3336823/v1, and others are available at 10.6084/m9.figshare.25232381 on figShare.

## Code availability

The codes for data analysis and API calls are available at 10.6084/m9.figshare.25232381 on figShare.
