# Supplementary material for: Prompt engineering in consistency and reliability with the evidence-based guideline for LLMs
Source: NPJ Digit Med. 2024 Feb 20;7:41. doi: 10.1038/s41746-024-01029-4 (PMC10879172; doi:10.1038/s41746-024-01029-4)
Supplement: Supplementary file 1 — Supplementary File [file 41746_2024_1029_MOESM1_ESM.pdf]

Displayed order of the supplementary file: 1. Supplementary Table 1-7; 2. Supplementary Note 1; 3. Supplementary References.

Supplementary Table 1. Multiple Comparisons within each model.

Model	Level	Sample size	Consistency				P value of multiple comparisons					
			IO	0-COT	COT	ROT	IO vs 0-COT	IO vs P-COT	IO vs ROT	0-COT vs P-COT	0-COT vs ROT	P-COT vs ROT
gpt-4-Web	Strong	40	0.3	0.525	0.75	0.775	0.041	0.000	0.000	0.036	0.019	0.793
gpt-4-API	Strong	40	0.15	0.125	0.3	0.35	0.745	0.108	0.039	0.056	0.018	0.633
gpt-4-API-0	Strong	40	0.125	0.125	0.625	0.7	1.000	0.000	0.000	0.000	0.000	0.478
Bard	Strong	40	0.075	0.35	0.55	0.3	0.003	0.000	0.010	0.072	0.633	0.024
gpt-3.5-Web	Strong	40	0	0	0.125	0.125	1.000	0.065	0.065	0.065	0.065	1.000
gpt-3.5-API	Strong	40	0.3	0.025	0.075	0.375	0.001	0.010	0.478	0.608	0.000	0.001
gpt-3.5-API-0	Strong	40	0.25	0	0.025	0.4	0.001	0.003	0.152	1.000	0.000	0.000
gpt-3.5-API-ft	Strong	40	0.775	0.35	0.275	0.35	0.000	0.000	0.000	0.469	1.000	0.469
gpt-3.5-APT-ft-0	Strong	40	0.75	0.5	0.475	0.6	0.021	0.012	0.152	0.823	0.369	0.262
gpt-4-Web	Moderate	40	0.375	0.3	0.325	0.4	0.478	0.639	0.818	0.809	0.348	0.485
gpt-4-API	Moderate	40	0.3	0.175	0.4	0.475	0.189	0.348	0.108	0.026	0.004	0.499
gpt-4-API-0	Moderate	40	0.15	0.075	0.575	0.425	0.479	0.000	0.007	0.000	0.000	0.180
Bard	Moderate	40	0.75	0.5	0.55	0.475	0.021	0.061	0.012	0.654	0.823	0.502
gpt-3.5-Web	Moderate	40	0.3	0.075	0.475	0.7	0.010	0.108	0.000	0.000	0.000	0.041
gpt-3.5-API	Moderate	40	0.625	0.05	0.525	0.525	0.000	0.366	0.366	0.000	0.000	1.000
gpt-3.5-API-0	Moderate	40	0.25	0.05	0.4	0.45	0.012	0.152	0.061	0.000	0.000	0.651
gpt-3.5-API-ft	Moderate	40	0.375	0.2	0.275	0.275	0.084	0.340	0.340	0.431	0.431	1.000
gpt-3.5-APT-ft-0	Moderate	40	0.5	0.15	0.125	0.275	0.001	0.000	0.039	0.745	0.172	0.094
gpt-4-Web	Limited	80	0.825	0.7	0.5	0.75	0.063	0.000	0.246	0.010	0.479	0.001
gpt-4-API	Limited	80	0.1125	0.1375	0.3	0.3375	0.633	0.003	0.001	0.013	0.003	0.611
gpt-4-API-0	Limited	80	0.1375	0.0875	0.2125	0.525	0.317	0.212	0.000	0.027	0.000	0.000
Bard	Limited	80	0.0125	0.5	0	0.025	0.000	1.000	1.000	0.000	0.000	0.477
gpt-3.5-Web	Limited	80	0.3875	0.075	0.625	0.2625	0.000	0.003	0.091	0.000	0.002	0.000
gpt-3.5-API	Limited	80	0.35	0.0375	0.2875	0.125	0.000	0.396	0.001	0.000	0.043	0.011
gpt-3.5-API-0	Limited	80	0.325	0.0875	0.1875	0	0.000	0.046	0.000	0.066	0.020	0.000
gpt-3.5-API-ft	Limited	80	0.3625	0.2375	0.45	0.2375	0.084	0.260	0.084	0.005	1.000	0.005
gpt-3.5-APT-ft-0	Limited	80	0.55	0.15	0.175	0.175	0.000	0.000	0.000	0.668	0.668	1.000
gpt-4-Web	Consensus	10	0.2	0.1	0.3	0						
gpt-4-API	Consensus	10	0.6	0	0.1	0						
gpt-4-API-0	Consensus	10	0.5	0	0	0						
Bard	Consensus	10	0	0.1	0.1	0						
gpt-3.5-Web	Consensus	10	0	0.1	0	0						
gpt-3.5-API	Consensus	10	0	0.2	0.2	0						
gpt-3.5-API-0	Consensus	10	0	0.3	0.5	0						
gpt-3.5-API-ft	Consensus	10	0.3	0.1	0	0.1						
gpt-3.5-APT-ft-0	Consensus	10	0	0.1	0	0						

Supplementary Table 2. Detailed statistical data of Fleiss kappa.

Model	Prompt	Fleiss Kappa	Standard Error	Z	p-value	95% CI Lower Bound	95% CI Upper Bound
gpt-4-Web	IO	0.524893396	0.038258799	13.71954704	0	0.522687889	0.527098904
	0-COT	0.450060655	0.034766393	12.94527884	0	0.448056475	0.452064835
	P-COT	0.334384216	0.036733266	9.103035242	0	0.332266652	0.336501781
	ROT	0.467327263	0.037700577	12.39575902	0	0.465153936	0.46950059
gpt-4-API	IO	0.287798908	0.029857642	9.639036591	0	0.286077703	0.289520113
	0-COT	0.06694496	0.030637295	2.185080615	0.028882936	0.06517881	0.06871111
	P-COT	0.331350002	0.029768315	11.13096253	0	0.329633946	0.333066058
	ROT	0.204944669	0.026616417	7.699934444	1.35447E-14	0.203410311	0.206479027
gpt-4-API-0	IO	0.524505302	0.026915758	19.48692296	0	0.522953688	0.526056916
	0-COT	0.284876606	0.028299244	10.06657997	0	0.283245238	0.286507974
	P-COT	0.659581023	0.030887726	21.35414636	0	0.657800436	0.661361609
	ROT	0.450779171	0.031907258	14.12779429	0	0.448939812	0.452618531
Bard	IO	0.373848987	0.03853985	9.700322829	0	0.371627278	0.376070696
	0-COT	0.354914686	0.035722781	9.93524787	0	0.352855373	0.356974
	P-COT	0.323342102	0.044135118	7.326186407	2.367E-13	0.320797842	0.325886361
	ROT	0.180487561	0.034777219	5.189821544	2.10496E-07	0.178482757	0.182492366
gpt-3.5-Web	IO	0.408970276	0.037690546	10.85073896	0	0.406797527	0.411143025
	0-COT	-0.001833421	0.039100212	-0.0468903	0.962600659	-0.004087433	0.000420592
	P-COT	0.276056638	0.036991681	7.462668122	8.4821E-14	0.273924176	0.2781891
	ROT	0.016009852	0.037968643	0.421659852	0.673273313	0.013821072	0.018198633
gpt-3.5-API	IO	0.188089623	0.037060713	5.075175565	3.87138E-07	0.185953182	0.190226064
	0-COT	0.003817305	0.032058709	0.119072328	0.905218054	0.001969215	0.005665395
	P-COT	0.031001754	0.033632839	0.921770353	0.356648375	0.02906292	0.032940588
	ROT	0.014312578	0.03397239	0.421300301	0.67353581	0.01235417	0.016270987
gpt-3.5-API-0	IO	0.984315896	0.029928688	32.88870875	0	0.982590595	0.986041197
	0-COT	0.461457233	0.037879225	12.18233051	0	0.459273607	0.463640859
	P-COT	0.533155296	0.034100894	15.63464256	0	0.53118948	0.535121112
	ROT	0.580764488	0.043314377	13.408123	0	0.578267542	0.583261435
gpt-3.5-ft	IO	0.162275566	0.033560472	4.835318307	1.32933E-06	0.160340903	0.164210228
	0-COT	0.02134232	0.030103334	0.708968645	0.478343935	0.019606951	0.023077688
	P-COT	0.064928377	0.030765838	2.110405003	0.034823486	0.063154817	0.066701938
	ROT	0.033293589	0.029835679	1.115898474	0.264465595	0.03157365	0.035013528
gpt-3.5-ft-0	IO	0.982215713	0.038486659	25.52094014	0	0.97999707	0.984434356
	0-COT	0.411904085	0.031635735	13.02021545	0	0.410080378	0.413727792
	P-COT	0.354638321	0.029254683	12.12244615	0	0.352951875	0.356324768
	ROT	0.397958187	0.031716143	12.54749624	0	0.396129844	0.399786529

Supplementary Table 3. Performance of LLMs in clinical medicine.

Author	Year	Type	Subject	Models	Result	Outperformed model
Yang Xi *et al.*[1]	2022	Queries	Comprehensive	GatorTron-large model	the GatorTron-large model achieved the best exact match score of 0.3155, outperforming BioBERT and ClinicalBERT by 6.8% and 7.5%, respectively.	
Zahir et al.[2]	2023	Diagnose	Comprehensive	GPT-4	Of 80 cases, 10 were excluded (4 were not diagnostic dilemmas; 6 were deleted for length). The 2 primary scorers agreed on 66% of scores. The AI model's top diagnosis agreed with the final diagnosis in 39% (27/70) of cases. In 64% of cases (45/70), the model included the final diagnosis in its differential.	
Karan et al.[3]	2023	Queries	Queries	Med-PaLM (instruction prompt-tuned Flan-PaLM); Flan-PaLM; PubMedGPT; DRAGON; BioLinkBERT; Galactica; PubMedBERT; GPT-Neo	Med-PaLM, a state-of-the-art large language model for medicine, is introduced and evaluated across several medical question answering tasks, demonstrating the promise of these models in this domain.	Med-PaLM
Amir et al.[4]	2023	Queries	Oncology	GPT-3.5 Bard	ChatGPT-3.5 answered 120 questions, with 85 (70.8%) correct answers, 14 (11.7%) partially correct answers, and 21 (17.5%) incorrect answers. Google Bard did not answer 23 of the 120 questions (19.2%), while it provided an answer for 97 questions (80.8%).	GPT-3.5
Isaac et al.[5]	2023	Queries	Ophthalmology	GPT-3.5	A total of 200 pairs of user questions and answers by AAO-affiliated ophthalmologists were evaluated. The mean (SD) accuracy for distinguishing between AI and human responses was 61.3% (9.7%). Of 800 evaluations of chatbot-written answers, 168 answers (21.0%) were marked as human-written, while 517 of 800 human-written answers (64.6%) were marked as AI-written.	
Zhi Wei et al.[6]	2023	Queries	Ophthalmology	GPT-3.5; GPT-4.0; Bard	ChatGPT-4.0 demonstrated superior accuracy, with 80.6% of responses rated as 'good', compared to 61.3% in ChatGPT-3.5 and 54.8% in Google Bard. ChatGPT-4.0 still performed superiorly in this domain, receiving 70% 'good' ratings, compared to 40% in ChatGPT-3.5 and 45% in Google Bard。	GPT-4
Yee et al.[7]	2023	Queries	Gastroenterology	GPT-3.5	We showed that ChatGPT regurgitated extensive knowledge of cirrhosis (79.1% correct) and HCC (74.0% correct), but only small proportions (47.3% in cirrhosis, 41.1% in HCC) were labeled as comprehensive. For the quality measures, the model answered 76.9% of questions correctly but failed to specify decision-making cut-offs and treatment durations.	
Wilhelm et al.[8]	2023	Queries	Comprehensive	Claude-instant-v1.0,;GPT-3.5-Turbo; Command-xlarge-nightly;	Claude-instant-v1.0 emerged with the highest mean mDISCERN score (3.35, 95% CI 3.23-3.46). In contrast, Bloomz lagged with the lowest score (1.07, 95% CI 1.03-1.10).	Claude-instant-v1.0

				Bloomz		
Harriet et al.[9]	2023	Queries	Gastroenterology	GPT-4	Five conditions were identified (gallstone disease, pancreatitis, liver cirrhosis, pancreatic cancer, and hepatocellular carcinoma). The median EQIP score across all conditions was 16 (IQR 14.5-18) for the total of 36 items. Divided by subsection, median scores for content, identification, and structure data were 10 (IQR 9.5-12.5), 1 (IQR 1-1), and 4 (IQR 4-5), respectively. Agreement between guideline recommendations and answers provided by ChatGPT was 60% (15/25).	
Shunsuke et al.[10]	2023	Diagnose	Neurology	GPT-4;<br>GPT-3.5;<br>Bard	A total of 25 cases of neurodegenerative disorders presented at Mayo Clinic brain bank Clinico-Pathological Conferences were analyzed. The LLMs provided multiple pathologic diagnoses and their rationales, which were compared with the final clinical diagnoses made by physicians. ChatGPT-3.5, ChatGPT-4, and Google Bard correctly made primary diagnoses in 32%, 52%, and 40% of cases, respectively, while correct diagnoses were included in 76%, 84%, and 76% of cases, respectively.	GPT-4
Yuki et al. [11]	2023	Queries	Rhinology	GPT-4	In our study, GPT-4 qualified all responses (100%) by mentioning the importance of discussing individual treatment plans with a health-care professional. Among 13 policies with weak evidence, defined as Grade D and no evidence, 9 responses from GPT-4 (69%) noted lack of consensus within existing guidelines.	
Takanobu et al.[12]	2023	Diagnose	Comprehensive	Bard	A total of 82 clinical descriptions (52 case reports and 30 mock cases) were used. The accuracy rates of physicians were still higher than Google Bard in the top 10 (56.1% vs 82.9%, P < .001), the top 5 (53.7% vs 78.0%, P = .002), and the top differential diagnosis (40.2% vs 64.6%, P = .003). When it came to mock cases, the performances of the differential-diagnosis lists by Google Bard were no different from those of the physicians in the top 10 (80.0% vs 96.6%, P = .11) and the top 5 (76.7% vs 96.6%, P = .06), except for those in the top diagnoses (60.0% vs 90.0%, P = .02).	
Krithi et al.[13]	2023	Queries	Ophthalmology	GPT-4;<br>GPT-3.5;<br>Bard	Responses were masked, randomly shuffled, and then graded by three consultant-level ophthalmologists for accuracy (poor, borderline, good) and comprehensiveness. Additionally, we evaluated the self-awareness capabilities (ability to self-check and self-correct) of the LLM-Chatbots. 89.2% of ChatGPT-4.0 responses were 'good'-rated, outperforming ChatGPT-3.5 (59.5%) and Google Bard (40.5%) significantly (all p < 0.001). All three LLM-Chatbots showed optimal mean comprehensiveness scores as well (ranging from 4.6 to 4.7 out of 5). However, they exhibited subpar to moderate self-awareness capabilities. Our study underscores the potential of ChatGPT-4.0 in delivering accurate and comprehensive responses to ocular symptom inquiries.	GPT-4
Hamish et al.[14]	2023	Diagnose	Emergency Medicine	GPT-3.5;<br>GPT-4.0;	Overall, 30 and 37 cases had sufficient data for diagnostic and triage analysis, respectively. The rate of top-1 diagnosis matches for Ada, ChatGPT 3.5, ChatGPT 4.0, and WebMD was 9 (30%), 12 (40%), 10 (33%), and 12 (40%), respectively, with a mean rate of 47% for the physicians. The rate of top-3 diagnostic matches for Ada, ChatGPT 3.5, ChatGPT 4.0, and WebMD was 19 (63%), 19 (63%), 15 (50%), and 17 (57%), respectively, with a mean rate of 69% for physicians. The distribution of triage results for Ada was 62% (n=23) agree, 14% unsafe (n=5), and 24% (n=9) too cautious; that for ChatGPT 3.5 was 59% (n=22) agree, 41% (n=15) unsafe, and 0% (n=0) too cautious; that for ChatGPT 4.0 was 76% (n=28) agree, 22% (n=8) unsafe, and 3% (n=1) too cautious; and that for WebMD	GPT-3.5 had high diagnostic accuracy but a high unsafe triage rate. GPT-4.0 had the poorest diagnostic accuracy, but a lower unsafe triage rate and the highest triage

					was 70% (n=26) agree, 19% (n=7) unsafe, and 11% (n=4) too cautious. The unsafe triage rate for ChatGPT 3.5 (41%) was significantly higher (P=.009) than that of Ada (14%).	agreement with the physicians.
Ana et al.[15]	2023	Queries	Dentistry	GPT-4;<br>GPT-3.5;<br>Bard	The answers generated by ChatGPT showed high consistency (85.44%). No significant differences in consistency were found based on question difficulty. In terms of answer accuracy, ChatGPT achieved an average accuracy of 57.33%.	
Rohaid et al.[16]	2023	Test	Neurology	GPT-3.5;<br>GPT-4	ChatGPT (GPT-3.5) and GPT-4 achieved scores of 73.4% (95% CI: 69.3%-77.2%) and 83.4% (95% CI: 79.8%-86.5%), respectively, relative to the user average of 72.8% (95% CI: 68.6%-76.6%).	GPT-4
Catherine et al.[17]	2023	Queries	Oncology	GPT-3.5	ChatGPT provided correct and comprehensive answers to 34 (53.1%) questions, correct but not comprehensive answers to 19 (29.7%) questions, partially incorrect answers to 10 (15.6%) questions, and completely incorrect answers to 1 (1.6%) question. Prevention and survivorship/QOL had the highest proportion of "correct" scores (scores of 1 or 2) at 22/24 (91.7%) and 15/16 (93.8%), respectively. ChatGPT performed less well in the treatment category, with 15/21 (71.4%) correct scores. It performed the worst in the diagnosis category with only 1/3 (33.3%) correct scores.	
Louis et al.[18]	2023	Test	Ophthalmology	GPT-4;<br>GPT-3.5;<br>Bing Chat	Human respondents had an average accuracy of 72.2%. ChatGPT-3.5 scored the lowest (58.8%), whereas ChatGPT-4.0 (71.6%) and Bing Chat (71.2%) performed comparably.	GPT-4
Fares et al.[19]	2023	Queries	Ophthalmology	GPT-3.5；GPT-4.0	GPT-4–0.3 (GPT-4 with a temperature of 0.3) achieved the highest accuracy among GPT-4 models, with 75.8% on the BCSC set and 70.0% on the OphthoQuestions set. The combined accuracy was 72.9%, which represents an 18.3% raw improvement in accuracy compared with GPT-3.5 (p<0.001). Human graders preferred responses from models with a temperature higher than 0 (more creative).	GPT-4-0.3 (temperature=0.3)
Thomas et al.[20]	2023	Test	Ophthalmology	GPT-4-0314;<br>Bard	GPT-4 answered 42 out of the 49 questions correctly, corresponding to a proportion correct of 0.857 (95% CI (0.753 to 0.961)). When scaled up to the full 180-question examination, this would translate to a score of approximately 154 out of 180 (95% CI (135 to 172)). Bard answered 22 out of the 49 questions correctly, corresponding to a proportion correct of 0.449 (95% CI (0.308 to 0.590)).	GPT-4-0314
Lauren et al.[21]	2023	Test	Dermatology	GPT-4;<br>GPT-3.5	We asked two iterations of ChatGPT: ChatGPT-3.5 and ChatGPT-4 84 multiple-choice sample questions from the sample dermatology SCE question bank. ChatGPT-3.5 achieved an overall score of 63.1%, and ChatGPT-4 scored 90.5% (a significant improvement in performance (p<0.001)).	GPT-4
Martin et al.[22]	2023	Diagnose	Rheumatology	GPT-4	ChatGPT-4 listed the correct diagnosis comparable often to rheumatologists as the top diagnosis 35% vs 39% (p = 0.30); as well as among the top 3 diagnoses, 60% vs 55%, (p = 0.38). In IRD-positive cases, ChatGPT-4 provided the top diagnosis in 71% vs 62% in the rheumatologists' analysis. Correct diagnosis was among the top 3 in 86% (ChatGPT-4) vs 74% (rheumatologists). In non-IRD cases, ChatGPT-4 provided the correct top diagnosis in 15% vs 27% in the rheumatologists' analysis. Correct diagnosis was among the top 3 in non-IRD cases in 46% of the ChatGPT-4 group vs 45% in the rheumatologists group. If only the first suggestion for diagnosis was considered, ChatGPT-4 correctly classified 58% of cases as IRD compared to 56% of the rheumatologists (p = 0.52).	
Xiao Yan et	2023	Diagnose	Ophthalmology	GPT-4	Among the 30 responses, 83.3% were considered "appropriate" by senior	

al.[23]					ophthalmologists. In the scenarios of simulated patient, family physician, and junior ophthalmologist, seven (70%), ten (100%), and eight (80%) responses were graded as “appropriate” by senior ophthalmologists. However, compared to the ground truth, GPT-4 could only output several possible diseases generally without “right” responses in the simulated patient scenarios. In contrast, in the simulated family physician scenario, 50% of GPT-4's responses were “right,” and in the simulated junior ophthalmologist scenario, the model achieved a higher “right” rate of 90%.	
Adem et al.[24]	2023	Test	Thoracic Surgery	GPT-3.5; GPT-4.0;	The overall mean score of students was 12.50 ± 1.20, corresponding to 83.33%. Moreover, ChatGPT-3.5 managed to surpass students' score of 12.5 with an average of 13.57 ± 0.49 questions correctly on average, while ChatGPT-4 answered 14 ± 0.76 questions correctly (83.3%, 90.48%, and 93.33%, respectively).	GPT-4
Jesse et al.[25]	2023	Test	Emergency Medicine	GPT-3.5<br>GPT-4.0<br>Bard-PaLM2<br>Bard-PaLM	All LLMs achieved a passing score, with scores with GPT 4.0 outperforming the average candidate.	GPT-4
Yoshitaka et al.[26]	2023	Test	Radiology	GPT-3.5<br>GPT-4<br>Bard	ChatGPT, GPT-4, and Google Bard correctly answered 40.8% (42 of 103), 65.0% (67 of 103), and 38.8% (40 of 103) of the questions, respectively. GPT-4 significantly outperformed ChatGPT by 24.2% ($p < 0.001$) and Google Bard by 26.2% ($p < 0.001$).	GPT-4

Supplementary Table 4. Prompt engineering in clinical medicine.

Author	Year	Involved Prompt Techniques	Description in the study	Application in the study
Karan et al.[3]	2023	Chain-of-thought prompting	COT involves augmenting each few-shot example in the prompt with a step-by-step breakdown and a coherent set of intermediate reasoning steps towards the final answer.	Study did not observe improvements using COT over the standard few-shot prompting strategy.<br>Using this self-consistency prompting, study observed considerable improvements over the standard few-shot prompting strategy for the Flan-PaLM 540B model on the MedQA and MedMCQA datasets. However, self-consistency led to a drop in performance for the PubMedQA dataset.
		Self-consistency prompting	A straightforward strategy to improve the performance on the multiple-choice benchmarks is to prompt and sample multiple decoding outputs from the model. The final answer is the one received the majority (or plurality) vote.	
		Few-shot prompting	The prompt to the model is designed to include few-shot examples describing the task through text-based demonstrations.	
Oliver et al.[27]	2023	Prompt techniques including “Preparing the LLM” and “Powerful prompts”	The study gives a few examples of prompt.The “Preparing the LLM” is described as ”I am going to provide X; I want you to output Y”<br>And “Powerful prompts” is described as “Powerful prompts guide LLMs in higher-order tasks, such as requesting more information for a conclusion”	The study presents several examples of prompt techniques applied in the field of ophthalmology and the study did not conduct a practice with these prompts on a dataset.
Tugba et al.[28]	2023	Zero-shot prompting	The prompt gives out an example input:<br>Question: What color is the sky?<br>Answer: [Output]	The study gives out some examples of the prompt techniques and a practical example to illustrate how prompt engineering can be used to structure a free-text radiology report and also translate the report into different languages
		Few-shot prompting	The study gives out an example input:<br>Question: What color is human blood?<br>Answer: red<br>Question: What color is grass?<br>Answer: green<br>Question: What color is the sky? Answer: [Output]	
		Instruction following	Please answer the following question to determine the usual color of the object that is mentioned.<br>Question: What color is the sky? Answer: [Output]	
		Chain-of-thought prompting	Question: What color is the sky at 12:00 pm?<br>Let’s think step-by-step and explain your thoughts.<br>Answer: [Output]	
Wan et al.[29]	2023	Zero-shot prompting	Zero-shot prompting requires the language model to respond without examples.	Overall, ChatGPT recognized 54 symptoms of Covid-19 among the 300 narratives. Among common symptoms, the sensitivity ranged from 0.853 to 1.000, and the specificity from 0.947 to 1.000. Among less common symptoms, the sensitivity ranged from 0.200 to 1.000, and the specificity from 0.993 to 1.000. Using few-shot prompting in GPT-4, the sensitivity was from 0.944 to 1.000, and the specificity was from 0.985 to 1.000 for common symptoms; while the sensitivity was from 0.625 to 1.000 and the
		Few-shot prompting	Few-shot prompting offers the language model several examples to shape its responses.	

				specificity was from 0.976 to 1.000 for less common symptoms.
Matthias et al.[30]	2023	User-defined prompts	Language-based foundation models, known as large language models (LLMs), can learn from textual instruction (user-defined prompts) with few or no examples in so-called few- or zero-shot settings.	Using user-defined prompts, GPT-4 outperformed GPT-3.5 in extracting oncologic phenotypes from free-text CT reports on lung cancer and demonstrated better oncologic reasoning with fewer confabulations.
Bertalan et al.[31]	2023	Various tips of writing prompts	This paper summarizes the current state of research about prompt engineering and, at the same time, aims at providing practical recommendations for the wide range of health care professionals to improve their interactions with LLMs.	

Supplementary Table 5. Designed prompts.

Prompt	Details
IO	Consider the following medical advice:<br><input the advice><br>Rate the medical advice using the following criteria, and make a selection from integer 1,2,3,4:<br>Score 1 (Consensus): There is no supporting evidence, and the medical advice is possibly based on common practice or expert opinion.<br>Score 2 (Limited): Supported by evidence from one or more low-quality studies with consistent findings, or evidence from a single moderate-quality study.<br>Score 3 (Moderate): Supported by evidence from two or more moderate-quality studies with consistent findings, or evidence from a single high-quality study.<br>Score 4 (Strong): Supported by evidence from two or more high-quality studies with consistent findings.
0-COT	Consider the following medical advice:<br><input the advice><br>Rate the medical advice using the following criteria, and make a selection from integer 1,2,3,4:<br>Score 1 (Consensus): There is no supporting evidence, and the medical advice is possibly based on common practice or expert opinion.<br>Score 2 (Limited): Supported by evidence from one or more low-quality studies with consistent findings, or evidence from a single moderate-quality study.<br>Score 3 (Moderate): Supported by evidence from two or more moderate-quality studies with consistent findings, or evidence from a single high-quality study.<br>Score 4 (Strong): Supported by evidence from two or more high-quality studies with consistent findings.<br><br>Complete the task above step by step.<br><br>Show your work of each step.
P-COT	Consider the following medical advice:<br><input the advice><br>Rate the medical advice using the following criteria, and make a selection from integer 1,2,3,4:<br>Score 1 (Consensus): There is no supporting evidence, and the medical advice is possibly based on common practice or expert opinion.<br>Score 2 (Limited): Supported by evidence from one or more low-quality studies with consistent findings, or evidence from a single moderate-quality study.<br>Score 3 (Moderate): Supported by evidence from two or more moderate-quality studies with consistent findings, or evidence from a single high-quality study.<br>Score 4 (Strong): Supported by evidence from two or more high-quality studies with consistent findings.<br><br>Complete the task above step by step:<br>Step 1: Understand the medical advice.

	Step 2: Gather evidence up until your knowledge cut-off in September 2021.<br>Step 3: Evaluate the quality and quantity of evidence.<br>Step 4: Evaluate the consistency of evidence.<br>Step 5: Rate the medical advice as the following format:<br>Score: <Insert an integer><br>Reasons: <Explain the reasons for the score given><br><br>Show your work of each step.
ROT	Consider the following medical advice:<br><input the advice><br>Rate the medical advice using the following criteria, and make a selection from integer 1,2,3,4:<br>Score 1 (Consensus): There is no supporting evidence, and the medical advice is possibly based on common practice or expert opinion.<br>Score 2 (Limited): Supported by evidence from one or more low-quality studies with consistent findings, or evidence from a single moderate-quality study.<br>Score 3 (Moderate): Supported by evidence from two or more moderate-quality studies with consistent findings, or evidence from a single high-quality study.<br>Score 4 (Strong): Supported by evidence from two or more high-quality studies with consistent findings.<br><br>Imagine three different experts (Expert A, Expert B, Expert C) are completing the task above step by step:<br>Step 1: Each expert independently understands the medical advice without knowing others' thoughts.<br>Step 2: Each expert independently gathers evidence up until your knowledge cut-off in September 2021 without knowing others' thoughts.<br>Step 3: Experts share all the gathered evidence.<br>Step 4: Each expert independently evaluates the quality and quantity of evidence gathered by all experts without knowing others' thoughts.<br>Step 5: Each expert independently evaluates the consistency of evidence gathered by all experts without knowing others' thoughts.<br>Step 6: Each expert independently rates the medical advice as the following format without knowing others' thoughts:<br>Score: <Insert an integer><br>Reasons: <Explain the reasons for the score given><br>Step 7: Experts discuss together, and experts should be open to reconsidering their initial evaluations based on the shared evidence and others' thoughts.<br>Step 8: After discussing, each expert rates the medical advice again as the following format:<br>Score: <Insert an integer><br>Reasons: <Explain the reasons for the score given><br>Step 9: Experts reach a final answer as the following format:<br>Score: <Insert an integer><br>Reasons: <Explain the reasons for the score given>

	I must emphasize that in Step 1, 2, 4, 5, and 6, experts absolutely should not know others' thoughts.<br><br>Show your work of each step.

Supplementary Table 6. Fine-tuning dataset.

Advice	Strength of the recommendation	Rationale	Question	Answer
Lateral wedge insoles are not recommended for patients with knee osteoarthritis.	4(Strong)	Although lateral heel wedges had historical support for their use in knee arthritis, contemporary studies have not shown a reliable improvement in pain relief, and no contemporary studies have shown sufficient functional improvement for patients suffering from knee arthritis to recommend using lateral wedge insoles. Lateral heel wedges can be prepared as an insert for the heel alone, or included in the heel of an independent arch support (i.e. lateral heel wedge arch support of LWAS) or built into the shoe itself (as was used by Hinman et al 2016). In the arthritic knee, medial compartment compression forces are commonly increased, especially in the knee with varus tibiofemoral alignment. The knee adduction moment can be calculated by gait analysis. The lateral wedge is thought to change the knee adduction moment thus relieving medial compartment pressure, hence relieving arthritic pain.<br><br>Our literature review screening culled several papers for analysis. Baker and Goggins 2007 was a high-quality study finding no important differences between insole and wedged insole. 90 patients were randomized to one treatment for 6 weeks followed by a 4-week washout period and then the opposite treatment. There were no major differences in pain during either phase of the study. More musculoskeletal symptoms and more blisters occurred with neutral insoles. No patient falls were attributed to the treatment alternatives.<br><br>Felson and Parke 2019 prescreened patients to eliminate those with patellofemoral OA and biomechanical non-responders. Lateral wedge insoles reduced knee pain, but the effect of treatment was small and was considered likely of clinical significance in only a minority of patients. 21 of 83 of patients did not show sufficient biomechanical correction. Only 28% of patients in the active phase of treatment had minimally important improvement whereas 22% of patients wearing neutral insoles reached the same level of improvement. 2 patients stopped treatment while wearing lateral wedge insoles (calf pain at night and increased knee pain) and 2 stopped while wearing neutral insoles (toe blister and increased knee pain). They also looked at volume of arthritic bone marrow lesions (BML) found by MRI and saw no significant difference in BML change between study and control groups.<br><br>In Bennell 2011, 89 patients with mild to moderate knee arthritis completed follow up with lateral insoles worn daily for 12 months. 90 patients completed follow up as the control group wearing neutral insoles. Pain relief after 12 months showed no significant difference between the groups.<br><br>In Hsieh 2016, 90 patients with Kellgren-Lawrence Grade 2 or higher radiographic changes were randomized to either a rigid insole with lateral wedge arch support (LWAS) or a soft insole with lateral wedge. Dropout rate was 20% with rigid and 15.6% with soft insoles over the 3-month long	Consider the following medical advice:<br>Lateral wedge insoles are not recommended for patients with knee osteoarthritis.<br>Rate the medical advice using the following criteria, and make a selection from integer 1,2,3,4:<br>Score 1 (Consensus): There is no supporting evidence, and the medical advice is possibly based on common practice or expert opinion.<br>Score 2 (Limited): Supported by evidence from one or more low-quality studies with consistent findings, or evidence from a single moderate-quality study.<br>Score 3 (Moderate): Supported by evidence from two or more moderate-quality studies with consistent findings, or evidence from a single high-quality study.<br>Score 4 (Strong): Supported by evidence from two or more high-quality studies with consistent findings.	Strength of recommendation: 4(Strong)<br>Rationale: Although lateral heel wedges had historical support for their use in knee arthritis, contemporary studies have not shown a reliable improvement in pain relief, and no contemporary studies have shown sufficient functional improvement for patients suffering from knee arthritis to recommend using lateral wedge insoles. Lateral heel wedges can be prepared as an insert for the heel alone, or included in the heel of an independent arch support (i.e. lateral heel wedge arch support of LWAS) or built into the shoe itself (as was used by Hinman et al 2016). In the arthritic knee, medial compartment compression forces are commonly increased, especially in the knee with varus tibiofemoral alignment. The knee adduction moment can be calculated by gait analysis. The lateral wedge is thought to change the knee adduction moment thus relieving medial compartment pressure, hence relieving arthritic pain.<br><br>Our literature review screening culled several papers for analysis. Baker and Goggins 2007 was a high-quality study finding no important differences between insole and wedged insole. 90 patients were randomized to one treatment for 6 weeks followed by a 4-week washout period and then the opposite treatment. There were no major differences in pain during either phase of the study. More musculoskeletal symptoms and more blisters occurred with neutral insoles. No patient falls were attributed to the treatment alternatives.<br><br>Felson and Parke 2019 prescreened patients to eliminate those with patellofemoral OA and biomechanical non-responders. Lateral wedge insoles reduced knee pain, but the effect of treatment was small and was considered likely of clinical significance in only a minority of patients. 21 of 83 of patients did not show sufficient biomechanical correction. Only 28% of patients in the active phase of treatment had minimally important improvement whereas 22% of patients wearing neutral insoles reached the same level of improvement. 2 patients stopped treatment while wearing lateral wedge insoles (calf pain at night and increased knee pain) and 2 stopped while wearing neutral insoles (toe blister and increased knee pain). They also looked at volume of arthritic bone marrow lesions (BML) found by MRI and saw no significant difference in BML change between study and control groups.<br><br>In Bennell 2011, 89 patients with mild to moderate knee arthritis completed follow up with lateral insoles worn daily for 12 months. 90 patients completed follow up as the control group wearing neutral insoles. Pain relief after 12 months showed no significant difference between the groups.<br><br>In Hsieh 2016, 90 patients with Kellgren-Lawrence Grade 2 or higher radiographic changes were randomized to either a rigid insole with lateral wedge arch support (LWAS) or a soft insole with lateral wedge. Dropout rate was 20% with rigid and 15.6% with soft insoles over the 3-month long study. They concluded that patients using the soft insole LWAS had improved pain and function. However, their primary data suggests better walking time and speed going up and down stairs with rigid LWAS.<br><br>Furthermore, pain was improved with soft LWAS only at the 3-month mark. Authors suggested longer-term follow-up for soft insoles.<br><br>Hinman 2016 evaluated an unloading shoe with stiff lateral midsole and 5-degree lateral wedge insole in comparison to a standard walking shoe. 164 patients were enrolled with 96% retention during the 6 months study. 83 patients received the unloading shoes and the control shoes. 14 of 83 stopped wearing the unloading shoes for various reasons and 8 of 81 stopped wearing the control shoes. 160 completed primary outcome measures at 6 months. There was no significant difference between groups with regard to pain or function, although both groups did show improvement.<br><br>20% of participants with the study shoes reported ankle and foot pain whereas 9% of control shoe participants did so. There was no difference in the reason to

		study. They concluded that patients using the soft insole LWAS had improved pain and function. However, their primary data suggests better walking time and speed going up and down stairs with rigid LWAS.<br><br>Furthermore, pain was improved with soft LWAS only at the 3-month mark. Authors suggested longer-term follow-up for soft insoles.<br><br>Hinman 2016 evaluated an unloading shoe with stiff lateral midsole and 5-degree lateral wedge insole in comparison to a standard walking shoe. 164 patients were enrolled with 96% retention during the 6 months study. 83 patients received the unloading shoes and the control shoes. 14 of 83 stopped wearing the unloading shoes for various reasons and 8 of 81 stopped wearing the control shoes. 160 completed primary outcome measures at 6 months. There was no significant difference between groups with regard to pain or function, although both groups did show improvement.<br><br>20% of participants with the study shoes reported ankle and foot pain whereas 9% of control shoe participants did so. There was no difference in the reason to discontinue treatment (unloading shoe 4% versus 2% control). Other reported adverse events were back pain, hip pain, knee pain, knee stiffness/swelling, and shin/calf pain. 2 of 83 experimental group patients reported an increase in knee pain with the unloading shoe and 2 of 81 control patients reported that the conventional shoe did not relieve knee pain.<br><br>Toda 2004 followed 84 knee female arthritis patients were followed for one month wearing either a hard rubber insole or urethane insole secured to the foot with a subtalar strap used for ankle sprains. 12 mm lateral wedge was manufactured for both. 17 of the 42 rubber insole patients had complications (foot pain in 8; popliteal pain in 6; low back pain in 3) versus 8 of the 42 using urethane insoles (popliteal pain in 4; foot pain in 3; low back pain in 1). All patients improved by the Lequesne Index with the urethane group achieving statistically significant improvement.<br><br>Niazi 2014 was a comparison of off-loading knee brace versus lateral wedge insole. 120 patients with both radiographic medial compartment arthritis and gene varum were randomized to either knee brace or lateral wedge insole. Pain improvement with the knee brace group was statistically significant compared to the lateral wedge insole, but clinically minor (VAS 3.97 in the study group compared to 4.53 in controls).<br><br>In Hatef 2013, 118 of 150 patients completed the 2-month long study (101 women and 17 men). Half were given LWAS, and the control group wore neutral insoles. Patient compliance was much worse in the LWAS group. They noted statistically significant decline in knee pain and EKFS in women in the LWAS, but not men. Overall, there was improvement in the LWAS group. There was a much higher non-compliance rate in the LWAS than with the neutral insoles with 29 of 57 patients stopping use of the insoles by weeks 5 to 6 of an 8-week study.<br><br>We identified one potential study within our literature which addressed the question of special shoe		discontinue treatment (unloading shoe 4% versus 2% control). Other reported adverse events were back pain, hip pain, knee pain, knee stiffness/swelling, and shin/calf pain. 2 of 83 experimental group patients reported an increase in knee pain with the unloading shoe and 2 of 81 control patients reported that the conventional shoe did not relieve knee pain.<br><br>Toda 2004 followed 84 knee female arthritis patients were followed for one month wearing either a hard rubber insole or urethane insole secured to the foot with a subtalar strap used for ankle sprains. 12 mm lateral wedge was manufactured for both. 17 of the 42 rubber insole patients had complications (foot pain in 8; popliteal pain in 6; low back pain in 3) versus 8 of the 42 using urethane insoles (popliteal pain in 4; foot pain in 3; low back pain in 1). All patients improved by the Lequesne Index with the urethane group achieving statistically significant improvement.<br><br>Niazi 2014 was a comparison of off-loading knee brace versus lateral wedge insole. 120 patients with both radiographic medial compartment arthritis and gene varum were randomized to either knee brace or lateral wedge insole. Pain improvement with the knee brace group was statistically significant compared to the lateral wedge insole, but clinically minor (VAS 3.97 in the study group compared to 4.53 in controls).<br><br>In Hatef 2013, 118 of 150 patients completed the 2-month long study (101 women and 17 men). Half were given LWAS, and the control group wore neutral insoles. Patient compliance was much worse in the LWAS group. They noted statistically significant decline in knee pain and EKFS in women in the LWAS, but not men. Overall, there was improvement in the LWAS group. There was a much higher non-compliance rate in the LWAS than with the neutral insoles with 29 of 57 patients stopping use of the insoles by weeks 5 to 6 of an 8-week study.<br><br>We identified one potential study within our literature which addressed the question of special shoe versus a conventional shoe. Nigg 2006 evaluated a training shoe which purports to convert a flat hard surface into "natural uneven ground", thus prompting increased muscle activity in the lower extremity. The control shoe was a standard walking shoe. 58 patients were enrolled in the study group and 67 in the control group. Both groups had one patient drop out (cumbersome shoes in the study group and increased knee pain with the control shoe). Pain with walking was improved at 12 weeks in both groups, without between groups difference. The study shoe showed increased pain relief at 3, 6, and 12 weeks. The control shoe showed increased pain relief at 3 and 12 weeks. They also reported improved balance from baseline in the study shoe at 12 weeks, which was not statistically significant.

		versus a conventional shoe. Nigg 2006 evaluated a training shoe which purports to convert a flat hard surface into "natural uneven ground", thus prompting increased muscle activity in the lower extremity. The control shoe was a standard walking shoe. 58 patients were enrolled in the study group and 67 in the control group. Both groups had one patient drop out (cumbersome shoes in the study group and increased knee pain with the control shoe). Pain with walking was improved at 12 weeks in both groups, without between groups difference. The study shoe showed increased pain relief at 3, 6, and 12 weeks. The control shoe showed increased pain relief at 3 and 12 weeks. They also reported improved balance from baseline in the study shoe at 12 weeks, which was not statistically significant.		
Canes could be used to improve pain and function in patients with knee osteoarthritis.	3(moderate)	Canes have been used since antiquity for lower extremity orthopaedic disorders. With that in mind, only a small number of modern studies have formally investigated use of a cane for knee osteoarthritis. Our literature review found one high-quality study (Jones 2012) showing support for use regarding moderate pain relief and another study (Van Ginckel 2019) of moderate quality showing no major improvement in pain.<br><br>Jones 2012 performed a comprehensive study of use of a cane for knee osteoarthritis. Their primary outcome was pain, but they also looked at function, general health, consumption of NSAIDs, and energy expenditure. 64 patients were selected out of a total of 323 patients nominated from a rheumatology clinic. The majority of possible patients (168 out of 323) refused to participate in the study. 32 patients were randomized to use of a cane for 60 days (EG, experimental group). The cane was cut to appropriate height, and they received instructions on its use. The 32 control patients (CG) were instructed to maintain a normal lifestyle and not to use auxiliary gait devices. At 30 and 60 days, the EG patients had less pain compared to controls. The greatest improvement was in the VAS (10 cm scale): EG averaging 3.84 cm and CG 5.95 cm at 60 days. The Lequesne scale (0-24) difference was only 2.53 (CG 15.09 and EG 12.56 at 60 days). At 60 days, the study group consumed fewer NSAIDs than control.<br><br>Van Ginckel 2019 evaluated use of a cane in patients with medial compartment knee osteoarthritis and bone marrow lesions (BML) on MRI. The primary intent of their study was to identify an effect on the size of BML by using a cane. Out of 1989 potential patients (contacted by phone or online) 231 were considered eligible for radiographic screening and of those, only 79 showed arthritic changes on plain films and BML on MRI and chose to continue with the study. 40 patients were assigned to use a cane whenever walking for the next 12 weeks. 39 control patients were instructed to maintain their usual lifestyle without any gait aids. Only one patient in the control group was lost to follow up. After 3 months, there was no significant improvement in BML size. Secondary information was obtained relative to clinical characteristics. There was no significant difference between the two groups with regards to knee pain (WOMAC scale) or quality of life (AQoL 6-D scale) although there was improvement in global knee pain in the group using the cane.	Consider the following medical advice:<br>Canes could be used to improve pain and function in patients with knee osteoarthritis.<br>Rate the medical advice using the following criteria, and make a selection from integer 1,2,3,4:<br>Score 1 (Consensus): There is no supporting evidence, and the medical advice is possibly based on common practice or expert opinion.<br>Score 2 (Limited): Supported by evidence from one or more low-quality studies with consistent findings, or evidence from a single moderate-quality study.<br>Score 3 (Moderate): Supported by evidence from two or more moderate-quality studies with consistent findings, or evidence from a single high-quality study.<br>Score 4 (Strong): Supported by evidence from two or more high-quality studies with consistent findings.	Strength of recommendation: 3(moderate)<br>Rationale: Canes have been used since antiquity for lower extremity orthopaedic disorders. With that in mind, only a small number of modern studies have formally investigated use of a cane for knee osteoarthritis. Our literature review found one high-quality study (Jones 2012) showing support for use regarding moderate pain relief and another study (Van Ginckel 2019) of moderate quality showing no major improvement in pain.<br><br>Jones 2012 performed a comprehensive study of use of a cane for knee osteoarthritis. Their primary outcome was pain, but they also looked at function, general health, consumption of NSAIDs, and energy expenditure. 64 patients were selected out of a total of 323 patients nominated from a rheumatology clinic. The majority of possible patients (168 out of 323) refused to participate in the study. 32 patients were randomized to use of a cane for 60 days (EG, experimental group). The cane was cut to appropriate height, and they received instructions on its use. The 32 control patients (CG) were instructed to maintain a normal lifestyle and not to use auxiliary gait devices. At 30 and 60 days, the EG patients had less pain compared to controls. The greatest improvement was in the VAS (10 cm scale): EG averaging 3.84 cm and CG 5.95 cm at 60 days. The Lequesne scale (0-24) difference was only 2.53 (CG 15.09 and EG 12.56 at 60 days). At 60 days, the study group consumed fewer NSAIDs than control.<br><br>Van Ginckel 2019 evaluated use of a cane in patients with medial compartment knee osteoarthritis and bone marrow lesions (BML) on MRI. The primary intent of their study was to identify an effect on the size of BML by using a cane. Out of 1989 potential patients (contacted by phone or online) 231 were considered eligible for radiographic screening and of those, only 79 showed arthritic changes on plain films and BML on MRI and chose to continue with the study. 40 patients were assigned to use a cane whenever walking for the next 12 weeks. 39 control patients were instructed to maintain their usual lifestyle without any gait aids. Only one patient in the control group was lost to follow up. After 3 months, there was no significant improvement in BML size. Secondary information was obtained relative to clinical characteristics. There was no significant difference between the two groups with regards to knee pain (WOMAC scale) or quality of life (AQoL 6-D scale) although there was improvement in global knee pain in the group using the cane.
Brace treatment could be used to improve function, pain, and quality of life in patients with knee osteoarthritis.	3(moderate)	Four high, four moderate and two low-quality studies were included for review, following the application of exclusion criteria by committee (Brouwer 2006b; Kirkley 1999; Callaghan 2015; Van Raaij 2010; Thoumie 2018; Hjartarson 2018; Petersen 2018; Niazi 2014; Hungerford 2013; Yu 2016). Three prospective randomized controlled trials compared bracing to control groups for	Consider the following medical advice:<br>Brace treatment could be used to improve function, pain, and quality of life in patients with knee osteoarthritis.<br>Rate the medical advice using the following criteria, and make a	Strength of recommendation: 3(moderate)<br>Rationale: Four high, four moderate and two low-quality studies were included for review, following the application of exclusion criteria by committee (Brouwer 2006b; Kirkley 1999; Callaghan 2015; Van Raaij 2010; Thoumie 2018; Hjartarson 2018; Petersen 2018; Niazi 2014; Hungerford 2013; Yu 2016). Three prospective randomized controlled trials compared bracing to control groups for treatment of symptomatic OA of the knee joint and found statistically significant and clinically

		treatment of symptomatic OA of the knee joint and found statistically significant and clinically meaningful improvement in patient symptoms related to symptomatic OA of the knee (Callaghan 2015; Thoumie 2018; Brouwer 2006b). Kirkley et al compared outcomes between valgus offloading brace, neoprene sleeve, and non-brace control for symptomatic OA and Varus alignment. This study reported statistically significant improvement in disease-specific quality of life and function in both study groups (Kirkley 1999). The study by Brouwer et al., comparing valgus bracing to a non-braced control, and reported no significant difference in functional assessment, PRO or pain; however, clinically significant improvements were noted in walking distance (1.25km[0.15,2.35]) for the brace group. Subgroup analysis demonstrated a greater positive effect of bracing in patients with varus alignment and more severe symptoms. Callaghan et al examined the effects of bracing for patella-femoral OA and found significant improvement from baseline VAS and KOOS pain scores. Finally, Hjartarson et al examined outcomes of bracing vs. placebo by removing valgus tension straps from the control group brace. In their study, they reported statistically significant and clinically meaningful improvements in KOOS sub-scores: symptoms, ADL, sports and recreation, and quality of life.<br><br>The Braces recommendation has been downgraded one level because of heterogeneity.	selection from integer 1,2,3,4:<br>Score 1 (Consensus): There is no supporting evidence, and the medical advice is possibly based on common practice or expert opinion.<br>Score 2 (Limited): Supported by evidence from one or more low-quality studies with consistent findings, or evidence from a single moderate-quality study.<br>Score 3 (Moderate): Supported by evidence from two or more moderate-quality studies with consistent findings, or evidence from a single high-quality study.<br>Score 4 (Strong): Supported by evidence from two or more high-quality studies with consistent findings.	meaningful improvement in patient symptoms related to symptomatic OA of the knee (Callaghan 2015; Thoumie 2018; Brouwer 2006b). Kirkley et al compared outcomes between valgus offloading brace, neoprene sleeve, and non-brace control for symptomatic OA and Varus alignment. This study reported statistically significant improvement in disease-specific quality of life and function in both study groups (Kirkley 1999). The study by Brouwer et al., comparing valgus bracing to a non-braced control, and reported no significant difference in functional assessment, PRO or pain; however, clinically significant improvements were noted in walking distance (1.25km[0.15,2.35]) for the brace group. Subgroup analysis demonstrated a greater positive effect of bracing in patients with varus alignment and more severe symptoms. Callaghan et al examined the effects of bracing for patella-femoral OA and found significant improvement from baseline VAS and KOOS pain scores. Finally, Hjartarson et al examined outcomes of bracing vs. placebo by removing valgus tension straps from the control group brace. In their study, they reported statistically significant and clinically meaningful improvements in KOOS sub-scores: symptoms, ADL, sports and recreation, and quality of life.<br><br>The Braces recommendation has been downgraded one level because of heterogeneity.
Turmeric may be helpful in reducing pain and improving function for patients with mild to moderate knee osteoarthritis.	2(limited)	One high-quality study (Srivastava 2016) that met inclusion criteria showed that Turmeric extract could be used over control to improve adverse events, function, and pain in patients with osteoarthritis of the knee.<br><br>This recommendation has been downgraded two levels because of inconsistency and the need for additional clarity of efficacy.	Consider the following medical advice:<br>Turmeric may be helpful in reducing pain and improving function for patients with mild to moderate knee osteoarthritis.<br>Rate the medical advice using the following criteria, and make a selection from integer 1,2,3,4:<br>Score 1 (Consensus): There is no supporting evidence, and the medical advice is possibly based on common practice or expert opinion.<br>Score 2 (Limited): Supported by evidence from one or more low-quality studies with consistent findings, or evidence from a single moderate-quality study.<br>Score 3 (Moderate): Supported by evidence from two or more moderate-quality studies with consistent findings, or evidence from a single high-quality study.<br>Score 4 (Strong): Supported by evidence from two or more high-quality studies with consistent findings.	Strength of recommendation: 2(limited)<br>Rationale: One high-quality study (Srivastava 2016) that met inclusion criteria showed that Turmeric extract could be used over control to improve adverse events, function, and pain in patients with osteoarthritis of the knee.<br><br>This recommendation has been downgraded two levels because of inconsistency and the need for additional clarity of efficacy.
Ginger extract may be helpful in reducing pain and improving function for patients with mild to moderate knee osteoarthritis.	2(limited)	One high-quality study (Zakeri 2011) and one moderate-quality study (Altman 2001) that met inclusion criteria showed that ginger extract may be used to improve pain in patients with osteoarthritis of the knee. However, there was no significant difference in function between ginger extract and control.<br><br>This recommendation has been downgraded two levels because of inconsistency and the need for additional clarity of efficacy.	Consider the following medical advice:<br>Ginger extract may be helpful in reducing pain and improving function for patients with mild to moderate knee osteoarthritis.<br>Rate the medical advice using the following criteria, and make a selection from integer 1,2,3,4:<br>Score 1 (Consensus): There is no supporting evidence, and the medical advice is possibly based on common practice or expert opinion.<br>Score 2 (Limited): Supported by evidence from one or more low-quality studies with consistent findings, or evidence from a single moderate-quality study.	Strength of recommendation: 2(limited)<br>Rationale: One high-quality study (Zakeri 2011) and one moderate-quality study (Altman 2001) that met inclusion criteria showed that ginger extract may be used to improve pain in patients with osteoarthritis of the knee. However, there was no significant difference in function between ginger extract and control.<br><br>This recommendation has been downgraded two levels because of inconsistency and the need for additional clarity of efficacy.

			Score 3 (Moderate): Supported by evidence from two or more moderate-quality studies with consistent findings, or evidence from a single high-quality study.<br>Score 4 (Strong): Supported by evidence from two or more high-quality studies with consistent findings.	
Glucosamine may be helpful in reducing pain and improving function for patients with mild to moderate knee osteoarthritis.	2(limited)	The majority of 6 high-quality trials (Reginster 2001, Cibere 2004, McAlindon 2004, Clegg 2006, Herrero-Beaumont 2007, Fransen 2015), the majority of 6 moderate-quality trials (Noack 1994, Houpt 1999, Rindone 2000, Pavelka 2002, Giordano 2009, and Shahine 2014), and 1 low-quality study that met inclusion criteria showed either improvement or no change in patient outcomes for those with osteoarthritis of the knee when taking glucosamine versus control.<br><br>This recommendation has been downgraded two levels because of inconsistency and the need for additional clarity of efficacy.	Consider the following medical advice:<br>Glucosamine may be helpful in reducing pain and improving function for patients with mild to moderate knee osteoarthritis.<br>Rate the medical advice using the following criteria, and make a selection from integer 1,2,3,4:<br>Score 1 (Consensus): There is no supporting evidence, and the medical advice is possibly based on common practice or expert opinion.<br>Score 2 (Limited): Supported by evidence from one or more low-quality studies with consistent findings, or evidence from a single moderate-quality study.<br>Score 3 (Moderate): Supported by evidence from two or more moderate-quality studies with consistent findings, or evidence from a single high-quality study.<br>Score 4 (Strong): Supported by evidence from two or more high-quality studies with consistent findings.	Strength of recommendation: 2(limited)<br>Rationale: The majority of 6 high-quality trials (Reginster 2001, Cibere 2004, McAlindon 2004, Clegg 2006, Herrero-Beaumont 2007, Fransen 2015), the majority of 6 moderate-quality trials (Noack 1994, Houpt 1999, Rindone 2000, Pavelka 2002, Giordano 2009, and Shahine 2014), and 1 low-quality study that met inclusion criteria showed either improvement or no change in patient outcomes for those with osteoarthritis of the knee when taking glucosamine versus control.<br><br>This recommendation has been downgraded two levels because of inconsistency and the need for additional clarity of efficacy.
Chondroitin may be helpful in reducing pain and improving function for patients with mild to moderate knee osteoarthritis.	2(limited)	The majority of 8 high-quality trials (Fransen 2015, Clegg 2006, Uebelhart 2004, Reginster 2017, Morita 2018, Zegels 2013, Kahan 2009, Rondanelli 2019) and 6 moderate-quality trials (Mazieres 2007, Moller 2010, Rondanelli 2019, Bourgeois 1998, Mazieres 2001, Bucsi 1998) that met inclusion criteria showed either improvement or no change in patient outcomes for those with osteoarthritis of the knee when taking chondroitin.<br><br>This recommendation has been downgraded two levels because of inconsistency and the need for additional clarity of efficacy.	Consider the following medical advice:<br>Chondroitin may be helpful in reducing pain and improving function for patients with mild to moderate knee osteoarthritis.<br>Rate the medical advice using the following criteria, and make a selection from integer 1,2,3,4:<br>Score 1 (Consensus): There is no supporting evidence, and the medical advice is possibly based on common practice or expert opinion.<br>Score 2 (Limited): Supported by evidence from one or more low-quality studies with consistent findings, or evidence from a single moderate-quality study.<br>Score 3 (Moderate): Supported by evidence from two or more moderate-quality studies with consistent findings, or evidence from a single high-quality study.<br>Score 4 (Strong): Supported by evidence from two or more high-quality studies with consistent findings.	Strength of recommendation: 2(limited)<br>Rationale: The majority of 8 high-quality trials (Fransen 2015, Clegg 2006, Uebelhart 2004, Reginster 2017, Morita 2018, Zegels 2013, Kahan 2009, Rondanelli 2019) and 6 moderate-quality trials (Mazieres 2007, Moller 2010, Rondanelli 2019, Bourgeois 1998, Mazieres 2001, Bucsi 1998) that met inclusion criteria showed either improvement or no change in patient outcomes for those with osteoarthritis of the knee when taking chondroitin.<br><br>This recommendation has been downgraded two levels because of inconsistency and the need for additional clarity of efficacy.
Vitamin D may be helpful in reducing pain and improving function for patients with mild to moderate knee osteoarthritis.	2(limited)	Three high-quality studies (McAlindon 2013, Sanghi 2013, and Jin 2016) and 1 moderate-quality study (Arden 2016) that met inclusion criteria showed either improvement or no significant difference in patient outcomes for those with osteoarthritis of the knee between Vitamin D and control.<br><br>This recommendation has been downgraded two levels because of inconsistency and the need for additional clarity of efficacy.	Consider the following medical advice:<br>Vitamin D may be helpful in reducing pain and improving function for patients with mild to moderate knee osteoarthritis.<br>Rate the medical advice using the following criteria, and make a selection from integer 1,2,3,4:<br>Score 1 (Consensus): There is no supporting evidence, and the medical advice is possibly based on common practice or expert opinion.	Strength of recommendation: 2(limited)<br>Rationale: Three high-quality studies (McAlindon 2013, Sanghi 2013, and Jin 2016) and 1 moderate-quality study (Arden 2016) that met inclusion criteria showed either improvement or no significant difference in patient outcomes for those with osteoarthritis of the knee between Vitamin D and control.<br><br>This recommendation has been downgraded two levels because of inconsistency and the need for additional clarity of efficacy.

			Score 2 (Limited): Supported by evidence from one or more low-quality studies with consistent findings, or evidence from a single moderate-quality study.<br>Score 3 (Moderate): Supported by evidence from two or more moderate-quality studies with consistent findings, or evidence from a single high-quality study.<br>Score 4 (Strong): Supported by evidence from two or more high-quality studies with consistent findings.	
Topical NSAIDs should be used to improve function and quality of life for treatment of osteoarthritis of the knee, when not contraindicated.	4(Strong)	Twelve high-quality studies (Baer 2005, Roth 2004, Conaghan 2013, Simon 2009, Kneer 2013, Rother 2013, Bookman 2004, Wadsworth 2016, Sandelin 1997, Dehghan 2019, Dehghan 2020, Rother 2007) and two moderate-quality studies (Barthel 2009, Ottillinger 2001) show that topical NSAIDs could result in improved function and quality of life over placebo gel. However, inconsistent evidence suggests no significant difference in pain and adverse events between topical NSAIDs and control.	Consider the following medical advice:<br>Topical NSAIDs should be used to improve function and quality of life for treatment of osteoarthritis of the knee, when not contraindicated.<br>Rate the medical advice using the following criteria, and make a selection from integer 1,2,3,4:<br>Score 1 (Consensus): There is no supporting evidence, and the medical advice is possibly based on common practice or expert opinion.<br>Score 2 (Limited): Supported by evidence from one or more low-quality studies with consistent findings, or evidence from a single moderate-quality study.<br>Score 3 (Moderate): Supported by evidence from two or more moderate-quality studies with consistent findings, or evidence from a single high-quality study.<br>Score 4 (Strong): Supported by evidence from two or more high-quality studies with consistent findings.	Strength of recommendation: 4(Strong)<br>Rationale: Twelve high-quality studies (Baer 2005, Roth 2004, Conaghan 2013, Simon 2009, Kneer 2013, Rother 2013, Bookman 2004, Wadsworth 2016, Sandelin 1997, Dehghan 2019, Dehghan 2020, Rother 2007) and two moderate-quality studies (Barthel 2009, Ottillinger 2001) show that topical NSAIDs could result in improved function and quality of life over placebo gel. However, inconsistent evidence suggests no significant difference in pain and adverse events between topical NSAIDs and control.
Supervised exercise, unsupervised exercise, and/or aquatic exercise are recommended over no exercise to improve pain and function for treatment of knee osteoarthritis.	4(Strong)	Ten high-quality studies were reviewed that compared a supervised exercise program to a non-exercise control (e.g., no treatment, heat only, education, usual primary care). (Christensen 2015, Holsgaard-Larsen 2018, Oliveira 2012, Willamson 2007, de Rooij 2017, Imoto 2012, Topp 2002, Hu 2020, Kim 2013, Chen 2014) Seven of these studies found greater improvements in pain, function, or both pain and function over the non-exercise control group (Oliveira 2012, de Rooij 2017, Imoto 2012, Topp 2002, Hu 2020, Kim 2013, Chen 2014).<br><br>One high-quality study and four moderate-quality studies were reviewed that compared supervised exercise to a non-supervised exercise program (e.g., home program, internet-based program, exercise brochure). (McCarthy 2004, Allen 2018, Yilmaz 2019, Tunay 2010, Bennell 2014). Patients from both groups received benefit from the interventions but there were mixed results as to whether supervised exercise was superior to the non-supervised exercise programs. It appears that both supervised or non-supervised exercise programs can result in improved pain and function in people with knee osteoarthritis.<br><br>Four high-quality studies and one moderate-quality study were reviewed that compared aquatic exercise to either usual primary care, education, or self-management. (Kuptniratsaikul 2019, Rewald 2020, Waller 2017, Munukka 2020, Dias 2017.) Three high-quality studies reported greater improvements in pain, function, or global ratings of improvement for the aquatic groups over the	Consider the following medical advice:<br>Supervised exercise, unsupervised exercise, and/or aquatic exercise are recommended over no exercise to improve pain and function for treatment of knee osteoarthritis.<br>Rate the medical advice using the following criteria, and make a selection from integer 1,2,3,4:<br>Score 1 (Consensus): There is no supporting evidence, and the medical advice is possibly based on common practice or expert opinion.<br>Score 2 (Limited): Supported by evidence from one or more low-quality studies with consistent findings, or evidence from a single moderate-quality study.<br>Score 3 (Moderate): Supported by evidence from two or more moderate-quality studies with consistent findings, or evidence from a single high-quality study.<br>Score 4 (Strong): Supported by evidence from two or more high-quality studies with consistent findings.	Strength of recommendation: 4(Strong)<br>Rationale: Ten high-quality studies were reviewed that compared a supervised exercise program to a non-exercise control (e.g., no treatment, heat only, education, usual primary care). (Christensen 2015, Holsgaard-Larsen 2018, Oliveira 2012, Willamson 2007, de Rooij 2017, Imoto 2012, Topp 2002, Hu 2020, Kim 2013, Chen 2014) Seven of these studies found greater improvements in pain, function, or both pain and function over the non-exercise control group (Oliveira 2012, de Rooij 2017, Imoto 2012, Topp 2002, Hu 2020, Kim 2013, Chen 2014).<br><br>One high-quality study and four moderate-quality studies were reviewed that compared supervised exercise to a non-supervised exercise program (e.g., home program, internet-based program, exercise brochure). (McCarthy 2004, Allen 2018, Yilmaz 2019, Tunay 2010, Bennell 2014). Patients from both groups received benefit from the interventions but there were mixed results as to whether supervised exercise was superior to the non-supervised exercise programs. It appears that both supervised or non-supervised exercise programs can result in improved pain and function in people with knee osteoarthritis.<br><br>Four high-quality studies and one moderate-quality study were reviewed that compared aquatic exercise to either usual primary care, education, or self-management. (Kuptniratsaikul 2019, Rewald 2020, Waller 2017, Munukka 2020, Dias 2017.) Three high-quality studies reported greater improvements in pain, function, or global ratings of improvement for the aquatic groups over the control groups. (Kuptniratsaikul 2019, Rewald 2020, Dias 2017) One high-quality study reported increased leisure time activity for the aquatic group compared to the control. (Waller 2017) One moderate-quality study compared aquatic exercise to land-based exercise. (Silva 2008) There was no difference in WOMAC pain and function scores reported between groups for this study, but the aquatic exercise group had less pain with walking compared to the land-based group. Although there may be some benefit from aquatic exercise, inconsistent results do not allow us to recommend aquatic exercise over land-based exercise at this time.

		control groups. (Kuptniratsaikul 2019, Rewald 2020, Dias 2017) One high-quality study reported increased leisure time activity for the aquatic group compared to the control. (Waller 2017) One moderate-quality study compared aquatic exercise to land-based exercise. (Silva 2008) There was no difference in WOMAC pain and function scores reported between groups for this study, but the aquatic exercise group had less pain with walking compared to the land-based group. Although there may be some benefit from aquatic exercise, inconsistent results do not allow us to recommend aquatic exercise over land-based exercise at this time.<br><br>Several studies examined clinical outcomes for different modes of exercise in patients with knee osteoarthritis. Ebnezar 2012 reported some improvement in anxiety measures when comparing yoga to non-yoga exercise. (Ebnezar 2012) Other studies compared weightbearing to non-weightbearing exercise (Bennell 2020, Jan 2009), high versus low resistance training (Jan 2008), isokinetic, isometric, and isotonic exercise (Huang 2005), and leg versus hip exercise (Lun 2015) and did not find substantial differences in the mode of exercise. It appears that exercise is beneficial, but the mode of exercise may not matter as much as engaging in any exercise program.		Several studies examined clinical outcomes for different modes of exercise in patients with knee osteoarthritis. Ebnezar 2012 reported some improvement in anxiety measures when comparing yoga to non-yoga exercise. (Ebnezar 2012) Other studies compared weightbearing to non-weightbearing exercise (Bennell 2020, Jan 2009), high versus low resistance training (Jan 2008), isokinetic, isometric, and isotonic exercise (Huang 2005), and leg versus hip exercise (Lun 2015) and did not find substantial differences in the mode of exercise. It appears that exercise is beneficial, but the mode of exercise may not matter as much as engaging in any exercise program.
Neuromuscular training (i.e. balance, agility, coordination) programs in combination with traditional exercise could be used to improve performance-based function and walking speed for treatment of knee osteoarthritis.	3(moderate)	Three high-quality studies (Fitzgerald 2011, Gomiero 2018, Apparao 2017) and two moderate-quality studies (Bennell 2014 and Diracoglu 2005) comparing neuromuscular training combined with traditional strength and joint mobility exercise programs to strength and joint mobility exercise alone were reviewed. There were no differences in knee pain reported between groups in any of the studies. There were mixed results on function measures with two studies reporting greater improvements in self-reported function (Apparao 2017, Diracoglu 2005) and two studies reporting greater improvements in walking speed (Bennell 2014, Diracoglu 2005) for the neuromuscular training group.<br><br>The Neuromuscular training recommendation has been downgraded one level because of inconsistent evidence.	Consider the following medical advice:<br>Neuromuscular training (i.e. balance, agility, coordination) programs in combination with traditional exercise could be used to improve performance-based function and walking speed for treatment of knee osteoarthritis.<br>Rate the medical advice using the following criteria, and make a selection from integer 1,2,3,4:<br>Score 1 (Consensus): There is no supporting evidence, and the medical advice is possibly based on common practice or expert opinion.<br>Score 2 (Limited): Supported by evidence from one or more low-quality studies with consistent findings, or evidence from a single moderate-quality study.<br>Score 3 (Moderate): Supported by evidence from two or more moderate-quality studies with consistent findings, or evidence from a single high-quality study.<br>Score 4 (Strong): Supported by evidence from two or more high-quality studies with consistent findings.	Strength of recommendation: 3(moderate)<br>Rationale: Three high-quality studies (Fitzgerald 2011, Gomiero 2018, Apparao 2017) and two moderate-quality studies (Bennell 2014 and Diracoglu 2005) comparing neuromuscular training combined with traditional strength and joint mobility exercise programs to strength and joint mobility exercise alone were reviewed. There were no differences in knee pain reported between groups in any of the studies. There were mixed results on function measures with two studies reporting greater improvements in self-reported function (Apparao 2017, Diracoglu 2005) and two studies reporting greater improvements in walking speed (Bennell 2014, Diracoglu 2005) for the neuromuscular training group.<br><br>The Neuromuscular training recommendation has been downgraded one level because of inconsistent evidence.
Self- management programs are recommended to improve pain and function for patients with knee osteoarthritis.	4(Strong)	Self-management programs refer to formalized training and education programs that are taught by both healthcare professionals and trained layperson instructors. They typically include several sessions over several weeks. These programs train people in several elements of self-management for osteoarthritis including medication compliance, pain management and pain coping strategies, joint protection strategies during physical activity, exercise advice, problem-solving approaches, and stress management techniques.<br><br>Four high-quality studies (Saffari 2018, Somers 2012, Hurley 2007, Omidi 2018) and one moderate-quality study (Coleman 2012) compared self-management to usual care or no treatment. These studies reported greater improvements in pain, function, or both compared to the control	Consider the following medical advice:<br>Self- management programs are recommended to improve pain and function for patients with knee osteoarthritis.<br>Rate the medical advice using the following criteria, and make a selection from integer 1,2,3,4:<br>Score 1 (Consensus): There is no supporting evidence, and the medical advice is possibly based on common practice or expert opinion.<br>Score 2 (Limited): Supported by evidence from one or more low-quality studies with consistent findings, or evidence from a single moderate-quality study.	Strength of recommendation: 4(Strong)<br>Rationale: Self-management programs refer to formalized training and education programs that are taught by both healthcare professionals and trained layperson instructors. They typically include several sessions over several weeks. These programs train people in several elements of self-management for osteoarthritis including medication compliance, pain management and pain coping strategies, joint protection strategies during physical activity, exercise advice, problem-solving approaches, and stress management techniques.<br><br>Four high-quality studies (Saffari 2018, Somers 2012, Hurley 2007, Omidi 2018) and one moderate-quality study (Coleman 2012) compared self-management to usual care or no treatment. These studies reported greater improvements in pain, function, or both compared to the control groups. In addition, some of these studies reported greater improvements in quality of life, pain catastrophizing, and self-efficacy in the self-management groups (Saffari 2018, Somers 2012).

		groups. In addition, some of these studies reported greater improvements in quality of life, pain catastrophizing, and self-efficacy in the self-management groups (Saffari 2018, Somers 2012).<br><br>One high-quality study (Marconcin 2018) and three moderate-quality studies examined the combined use of self-management and exercise to either groups that received self-management or exercise alone (Bennell 2016) or usual care (Yip 2007, Kao 2012). Yip et al. reported greater improvements in pain, time spent in leisure activities, and self-efficacy, compared to usual care. (Yip 2007) reported greater improvements in pain and function compared to the control groups. Bennell, et al, reported improvements in pain and function in all groups. There were no differences between groups on pain measures but the combined use of self-management (i.e., pain coping skills training) and exercise had greater improvements in function compared to those receiving only self-management or exercise (Bennell 2016).<br><br>An attempt was made to examine the literature on cognitive-behavioral therapy (CBT) in the management of people with knee osteoarthritis. One high-quality study (Helminen 2015) and 4 moderate-quality (Focht 2012, Focht 2017, Smith 2015, Lerman 2017) studies were reviewed. Control groups consisted of usual care (Helminen 2015), traditional exercise approaches for knee osteoarthritis (Focht 2012, Focht 2017), or behavioral desensitization (Smith 2015, Lerman 2017). Inconsistency in outcome results across studies made it difficult to provide a recommendation for this intervention approach at this time.	Score 3 (Moderate): Supported by evidence from two or more moderate-quality studies with consistent findings, or evidence from a single high-quality study.<br>Score 4 (Strong): Supported by evidence from two or more high-quality studies with consistent findings.	One high-quality study (Marconcin 2018) and three moderate-quality studies examined the combined use of self-management and exercise to either groups that received self-management or exercise alone (Bennell 2016) or usual care (Yip 2007, Kao 2012). Yip et al. reported greater improvements in pain, time spent in leisure activities, and self-efficacy, compared to usual care. (Yip 2007) reported greater improvements in pain and function compared to the control groups. Bennell, et al, reported improvements in pain and function in all groups. There were no differences between groups on pain measures but the combined use of self-management (i.e., pain coping skills training) and exercise had greater improvements in function compared to those receiving only self-management or exercise (Bennell 2016).<br><br>An attempt was made to examine the literature on cognitive-behavioral therapy (CBT) in the management of people with knee osteoarthritis. One high-quality study (Helminen 2015) and 4 moderate-quality (Focht 2012, Focht 2017, Smith 2015, Lerman 2017) studies were reviewed. Control groups consisted of usual care (Helminen 2015), traditional exercise approaches for knee osteoarthritis (Focht 2012, Focht 2017), or behavioral desensitization (Smith 2015, Lerman 2017). Inconsistency in outcome results across studies made it difficult to provide a recommendation for this intervention approach at this time.
Patient education programs are recommended to improve pain in patients with knee osteoarthritis.	4(Strong)	Six high-quality studies (Saffari 2018, Somers 2012, Cagnin 2019, Gilbert 2018, Baker 2019, Berman 2004) thirteen moderate quality study (Brosseau 2012, Allen 2010, O'Brien 2018, Allen 2010, Bennell 2017, Marra 2012, Rezende 2017, Sandeghi 2019, Rodriguez da Silva 2017, Rini 2015, Moseng 2020, Chen 2020, Ravaud 2009) and two limited quality studies compared patient education and control. These studies reported more significant improvements in pain compared to the control groups.<br><br>Patient education programs in studies overlap with self-management programs. Patient education programs vary from patient handout, 2+ hour DVD, one-day education to multiple sessions over a month (Saffari 2018, Cagnin 2019, Brosseau 2012, O'Brien 2018, Rezende 2017, Rodriguez da Silva 2017, Rini 2015). Many studies are challenging to evaluate the effects of education because they involve exercise classes and other proven interventions (Marra 2012, Ravaud 2009). Self-management programs train people in several elements of self-management for osteoarthritis (1148), including medication compliance, pain management, and pain coping strategies, joint protection strategies (1149) during physical activity, exercise advice, problem-solving approaches, and stress management techniques. Patient education programs may not be as labor-intensive, and further work is needed to identify the amount of education needed to improve patient-related outcome measures, like pain.<br><br>Programs that focused on education are two high quality (Saffari 2018, Cagnin 2019) and four moderate quality (Brosseau 2012, O'Brien 2018, Rodriguez da Silva 2017, Rini 2015). Saffari used seven (7) group sessions over one month and provided a CD-ROM and booklet describing	Consider the following medical advice:<br>Patient education programs are recommended to improve pain in patients with knee osteoarthritis.<br>Rate the medical advice using the following criteria, and make a selection from integer 1,2,3,4:<br>Score 1 (Consensus): There is no supporting evidence, and the medical advice is possibly based on common practice or expert opinion.<br>Score 2 (Limited): Supported by evidence from one or more low-quality studies with consistent findings, or evidence from a single moderate-quality study.<br>Score 3 (Moderate): Supported by evidence from two or more moderate-quality studies with consistent findings, or evidence from a single high-quality study.<br>Score 4 (Strong): Supported by evidence from two or more high-quality studies with consistent findings.	Strength of recommendation: 4(Strong)<br>Rationale: Six high-quality studies (Saffari 2018, Somers 2012, Cagnin 2019, Gilbert 2018, Baker 2019, Berman 2004) thirteen moderate quality study (Brosseau 2012, Allen 2010, O'Brien 2018, Allen 2010, Bennell 2017, Marra 2012, Rezende 2017, Sandeghi 2019, Rodriguez da Silva 2017, Rini 2015, Moseng 2020, Chen 2020, Ravaud 2009) and two limited quality studies compared patient education and control. These studies reported more significant improvements in pain compared to the control groups.<br><br>Patient education programs in studies overlap with self-management programs. Patient education programs vary from patient handout, 2+ hour DVD, one-day education to multiple sessions over a month (Saffari 2018, Cagnin 2019, Brosseau 2012, O'Brien 2018, Rezende 2017, Rodriguez da Silva 2017, Rini 2015). Many studies are challenging to evaluate the effects of education because they involve exercise classes and other proven interventions (Marra 2012, Ravaud 2009). Self-management programs train people in several elements of self-management for osteoarthritis (1148), including medication compliance, pain management, and pain coping strategies, joint protection strategies (1149) during physical activity, exercise advice, problem-solving approaches, and stress management techniques. Patient education programs may not be as labor-intensive, and further work is needed to identify the amount of education needed to improve patient-related outcome measures, like pain.<br><br>Programs that focused on education are two high quality (Saffari 2018, Cagnin 2019) and four moderate quality (Brosseau 2012, O'Brien 2018, Rodriguez da Silva 2017, Rini 2015). Saffari used seven (7) group sessions over one month and provided a CD-ROM and booklet describing preventive lifestyle procedures and the importance of treatment adherence (Saffari 2018). They found improvement in SF-12 and pain scores. Cagnin used an educational session with a physical therapist who demonstrated how recommended exercises should be performed and how patients can manage their pain. They demonstrated improvement in KOOS pain scores (Cagnin 2019). Brousseau looked at education (educational pamphlet) vs. walking and education vs. walking and behavioral intervention (Brouseau 2012). There was a non-clinically significant improvement in pain in the education-only group at 12 months compared to walking and behavioral intervention. O'Brien used weight loss education, where trained telephone interviewers provided brief advice and education about the benefits of weight loss and physical activity for knee osteoarthritis immediately after randomization [O'Brien 2018]. The intervention group provided an evidence-based public health non-disease-specific telephone-based coaching

		preventive lifestyle procedures and the importance of treatment adherence (Saffari 2018). They found improvement in SF-12 and pain scores. Cagnin used an educational session with a physical therapist who demonstrated how recommended exercises should be performed and how patients can manage their pain. They demonstrated improvement in KOOS pain scores (Cagnin 2019). Brousseau looked at education (educational pamphlet) vs. walking and education vs. walking and behavioral intervention (Brouseau 2012). There was a non-clinically significant improvement in pain in the education-only group at 12 months compared to walking and behavioral intervention. O'Brien used weight loss education, where trained telephone interviewers provided brief advice and education about the benefits of weight loss and physical activity for knee osteoarthritis immediately after randomization [O'Brien 2018]. The intervention group provided an evidence-based public health non-disease-specific telephone-based coaching service funded by the local Australian state government to support adults in making sustained lifestyle improvements, including diet, physical activity, and achieving a healthy weight and, where appropriate, access smoking cessation services. They did not find an added benefit from the coaching service over the brief telephone education in pain nor WOMAC scores. Rini compared an internet-based app (PainCoach) [http://tri.ad/projects-2/] to usual care and found a non-clinically significant reduction in VAS pain scores (Rini 2015). Rodriguez da Silva used a single day (Saturday, from 8 a.m. to 5 p.m.), which included seven lectures of 30 min by each professional team, and 60-min workshops by the physical education, physical therapy, and occupational therapy professionals, approaching the importance of their area in knee OA treatment/management. The study did not report pain scores but did note an increase in mobility with improvements in the get-up and go test. The two high-quality and four moderate-quality studies showed improved pain scores from the education given during educational sessions. Most studies (15 of the 21) incorporate education with other interventions; therefore, it is impossible to isolate the effects of education in these other 15 studies.<br><br>One high quality (Gilbert 2018) and three moderate quality (Rezende 2017, Chen 202, Ravaud 2009) used the transtheoretical model (TTM) and motivational interviewing to improve osteoarthritis treatment adherence. These studies showed improvement in WOMAC pain scores. TTM has been used successfully in other conditions that benefit from lifestyle changes [PMID: 24500864].		service funded by the local Australian state government to support adults in making sustained lifestyle improvements, including diet, physical activity, and achieving a healthy weight and, where appropriate, access smoking cessation services. They did not find an added benefit from the coaching service over the brief telephone education in pain nor WOMAC scores. Rini compared an internet-based app (PainCoach) [http://tri.ad/projects-2/] to usual care and found a non-clinically significant reduction in VAS pain scores (Rini 2015). Rodriguez da Silva used a single day (Saturday, from 8 a.m. to 5 p.m.), which included seven lectures of 30 min by each professional team, and 60-min workshops by the physical education, physical therapy, and occupational therapy professionals, approaching the importance of their area in knee OA treatment/management. The study did not report pain scores but did note an increase in mobility with improvements in the get-up and go test. The two high-quality and four moderate-quality studies showed improved pain scores from the education given during educational sessions. Most studies (15 of the 21) incorporate education with other interventions; therefore, it is impossible to isolate the effects of education in these other 15 studies.<br><br>One high quality (Gilbert 2018) and three moderate quality (Rezende 2017, Chen 202, Ravaud 2009) used the transtheoretical model (TTM) and motivational interviewing to improve osteoarthritis treatment adherence. These studies showed improvement in WOMAC pain scores. TTM has been used successfully in other conditions that benefit from lifestyle changes [PMID: 24500864].
Sustained weight loss is recommended to improve pain and function in overweight and obese patients with knee osteoarthritis.	3(moderate)	There were 1 high (Jenkinson et. al 2009), 1 moderate (Miller 2006), and 2 low strength (Focht 2005, Rejeski 2002) studies evaluating diet and exercise as weight loss interventions to treat knee osteoarthritis. Overall pain and function improved with weight loss achieved through a combination of diet and exercise. However, when evaluating only diet vs control, 2 high (Bliddal 2011, Christensen 2015), 2 moderate (Messier 2013, Mihalko 2018) and 2 low strength (Rejeski 2002, Fochyt 2005) there was no clear clinically significant change in patient outcomes. Specifically, Christensen et al, 2015 published a high-quality study investigating the effect of weight on symptoms of knee osteoarthritis. They showed no significant difference in pain and function at 1 year. Bliddal et al, 2010 published another high-quality study which investigated the effect of weight loss on symptoms of knee OA in the obese patient, showing that perceived pain (via WOMAC) was significantly lessened despite not being able to show improvement in function and quality of life at 1 year.	Consider the following medical advice:<br>Sustained weight loss is recommended to improve pain and function in overweight and obese patients with knee osteoarthritis.<br>Rate the medical advice using the following criteria, and make a selection from integer 1,2,3,4:<br>Score 1 (Consensus): There is no supporting evidence, and the medical advice is possibly based on common practice or expert opinion.<br>Score 2 (Limited): Supported by evidence from one or more low-quality studies with consistent findings, or evidence from a single moderate-quality study.<br>Score 3 (Moderate): Supported by evidence from two or more moderate-quality studies with consistent findings, or evidence from a single high-quality study.	Strength of recommendation: 3(moderate)<br>Rationale: There were 1 high (Jenkinson et. al 2009), 1 moderate (Miller 2006), and 2 low strength (Focht 2005, Rejeski 2002) studies evaluating diet and exercise as weight loss interventions to treat knee osteoarthritis. Overall pain and function improved with weight loss achieved through a combination of diet and exercise. However, when evaluating only diet vs control, 2 high (Bliddal 2011, Christensen 2015), 2 moderate (Messier 2013, Mihalko 2018) and 2 low strength (Rejeski 2002, Fochyt 2005) there was no clear clinically significant change in patient outcomes. Specifically, Christensen et al, 2015 published a high-quality study investigating the effect of weight on symptoms of knee osteoarthritis. They showed no significant difference in pain and function at 1 year. Bliddal et al, 2010 published another high-quality study which investigated the effect of weight loss on symptoms of knee OA in the obese patient, showing that perceived pain (via WOMAC) was significantly lessened despite not being able to show improvement in function and quality of life at 1 year.<br><br>There were 2 moderate strength studies (Messier 2013, Mihalko 2018) which evaluated diet vs exercise, which favored exercises. To note, Messier et al 2013 published results of the IDEA trial with moderate quality study which was an attempt to determine if a 10% reduction in body weight (induced by diet, with or without exercise) would improve "clinical and mechanistic" outcomes in sedentary lifestyle patients (BMI 27 thru 41). Interestingly, in this primary study, they were unable to show an improvement in WOMAC pain but they did show improvement in the WOMAC function subscale, and also showed improvements in the 6-minute

		There were 2 moderate strength studies (Messier 2013, Mihalko 2018) which evaluated diet vs exercise, which favored exercises. To note, Messier et al 2013 published results of the IDEA trial with moderate quality study which was an attempt to determine if a 10% reduction in body weight (induced by diet, with or without exercise) would improve "clinical and mechanistic" outcomes in sedentary lifestyle patients (BMI 27 thru 41). Interestingly, in this primary study, they were unable to show an improvement in WOMAC pain but they did show improvement in the WOMAC function subscale, and also showed improvements in the 6-minute walk test.<br><br>Given the current evidence, it is at the discretion of the surgeon as to which approach is utilized to address weight loss, however a combination of diet and exercises appears to be the preferred alternative.<br><br>The Weight Loss Intervention recommendation has been downgraded one level because of inconsistent evidence.	Score 4 (Strong): Supported by evidence from two or more high-quality studies with consistent findings.	walk test.<br><br>Given the current evidence, it is at the discretion of the surgeon as to which approach is utilized to address weight loss, however a combination of diet and exercises appears to be the preferred alternative.<br><br>The Weight Loss Intervention recommendation has been downgraded one level because of inconsistent evidence.
Manual therapy in addition to an exercise program may be used to improve pain and function in patients with knee osteoarthritis.	2(limited)	Manual therapy consists of maneuvers applied with manual force from the treating therapist to the patient's body to improve joint mobility and/or relieve pain. The techniques may generally consist of manually applied joint mobilization techniques, manually applied joint range of motion and/or muscle stretching, and soft tissue massage. One high-quality study (Fitzgerald 2016) and one moderate-quality study (Deyle 2000) were reviewed that examined manual therapy combined with exercise compared to exercise alone (Fitzgerald 2016) or non-therapeutic ultrasound (placebo physical therapy) in subjects with knee osteoarthritis (Deyle 2000). Fitzgerald, et al, reported that both groups yielded significant improvements in clinical outcomes from baseline, but the manual therapy group had greater improvements in the WOMAC total score and were more likely to meet the OMERACT-OARSI Responder Criteria at the 9-week follow-up (Fitzgerald 2016). While both groups demonstrated sustained improvements in clinical outcomes at 1 year, there was no difference between groups on any measures at this timepoint. Deyle et al. reported similar findings with the manual therapy and exercise group demonstrating greater improvements at 8 weeks but no significant differences between groups at 1 year (Deyle 2000).<br><br>The Manual Therapy recommendation has been downgraded one level because of inconsistent evidence and lack of internal consistency with recommendations of equal supporting evidence.	Consider the following medical advice:<br>Manual therapy in addition to an exercise program may be used to improve pain and function in patients with knee osteoarthritis.<br>Rate the medical advice using the following criteria, and make a selection from integer 1,2,3,4:<br>Score 1 (Consensus): There is no supporting evidence, and the medical advice is possibly based on common practice or expert opinion.<br>Score 2 (Limited): Supported by evidence from one or more low-quality studies with consistent findings, or evidence from a single moderate-quality study.<br>Score 3 (Moderate): Supported by evidence from two or more moderate-quality studies with consistent findings, or evidence from a single high-quality study.<br>Score 4 (Strong): Supported by evidence from two or more high-quality studies with consistent findings.	Strength of recommendation: 2(limited)<br>Rationale: Manual therapy consists of maneuvers applied with manual force from the treating therapist to the patient's body to improve joint mobility and/or relieve pain. The techniques may generally consist of manually applied joint mobilization techniques, manually applied joint range of motion and/or muscle stretching, and soft tissue massage. One high-quality study (Fitzgerald 2016) and one moderate-quality study (Deyle 2000) were reviewed that examined manual therapy combined with exercise compared to exercise alone (Fitzgerald 2016) or non-therapeutic ultrasound (placebo physical therapy) in subjects with knee osteoarthritis (Deyle 2000). Fitzgerald, et al, reported that both groups yielded significant improvements in clinical outcomes from baseline, but the manual therapy group had greater improvements in the WOMAC total score and were more likely to meet the OMERACT-OARSI Responder Criteria at the 9-week follow-up (Fitzgerald 2016). While both groups demonstrated sustained improvements in clinical outcomes at 1 year, there was no difference between groups on any measures at this timepoint. Deyle et al. reported similar findings with the manual therapy and exercise group demonstrating greater improvements at 8 weeks but no significant differences between groups at 1 year (Deyle 2000).<br><br>The Manual Therapy recommendation has been downgraded one level because of inconsistent evidence and lack of internal consistency with recommendations of equal supporting evidence.
Massage may be used in addition to usual care to improve pain and function in patients with knee osteoarthritis.	2(limited)	Two high-quality studies (Pehlivan 2018, Sansila 2019) and one moderate-quality study (Perlman 2018) were reviewed that examined massage plus usual care to a usual care control group. Pehlivan et al. applied a leg massage focusing primarily on tissues around the knee for a total of 6 sessions over 3 weeks (Pehlivan 2018). Subjects receiving massage had greater improvements in knee pain at 4 weeks follow-up compared to the usual care control group, but there was no difference between groups at 8 weeks. There were also no differences in function between groups at either time point. Perlman et al. applied a 60-minute total body massage one time per week for 8 weeks, followed by bi-weekly sessions for 52 weeks (Perlman 2018). There was greater improvement in pain and function in the massage group at the 8-week follow-up, but these effects were not sustained for any of the longer-term follow-up time points.	Consider the following medical advice:<br>Massage may be used in addition to usual care to improve pain and function in patients with knee osteoarthritis.<br>Rate the medical advice using the following criteria, and make a selection from integer 1,2,3,4:<br>Score 1 (Consensus): There is no supporting evidence, and the medical advice is possibly based on common practice or expert opinion.<br>Score 2 (Limited): Supported by evidence from one or more low-quality studies with consistent findings, or evidence from a single moderate-quality study.<br>Score 3 (Moderate): Supported by evidence from two or more	Strength of recommendation: 2(limited)<br>Rationale: Two high-quality studies (Pehlivan 2018, Sansila 2019) and one moderate-quality study (Perlman 2018) were reviewed that examined massage plus usual care to a usual care control group. Pehlivan et al. applied a leg massage focusing primarily on tissues around the knee for a total of 6 sessions over 3 weeks (Pehlivan 2018). Subjects receiving massage had greater improvements in knee pain at 4 weeks follow-up compared to the usual care control group, but there was no difference between groups at 8 weeks. There were also no differences in function between groups at either time point. Perlman et al. applied a 60-minute total body massage one time per week for 8 weeks, followed by bi-weekly sessions for 52 weeks (Perlman 2018). There was greater improvement in pain and function in the massage group at the 8-week follow-up, but these effects were not sustained for any of the longer-term follow-up time points.<br><br>The Massage recommendation has been downgraded one level because of inconsistent evidence and due to the lack of internal consistency with recommendations of equal supporting evidence.

		The Massage recommendation has been downgraded one level because of inconsistent evidence and due to the lack of internal consistency with recommendations of equal supporting evidence.	moderate-quality studies with consistent findings, or evidence from a single high-quality study.<br>Score 4 (Strong): Supported by evidence from two or more high-quality studies with consistent findings.	
FDA-approved laser treatment may be used to improve pain and function in patients with knee osteoarthritis.	2(limited)	A meta-analysis was performed using pain data from two high-quality studies (Gur 2003, Nazari 2018) and one moderate-quality (Marquina 2012) study examining high-intensity laser treatment compared to either placebo laser treatment or no treatment groups. The results of the analysis are provided in Figure 10 in the appendix. The overall findings were in favor of the laser intervention over the sham or no-treatment groups. In addition, two of these studies reported greater improvements in function (Gur 2003, Nazari 2018). Gur 2003 also compared high dose vs. low dose laser treatment on clinical outcomes and found no significant difference between the groups.<br><br>The Laser Treatment recommendation has been downgraded two levels because of feasibility, usage in practice, and a lack of internal consistency with recommendations of equal supporting evidence.	Consider the following medical advice:<br>FDA-approved laser treatment may be used to improve pain and function in patients with knee osteoarthritis.<br>Rate the medical advice using the following criteria, and make a selection from integer 1,2,3,4:<br>Score 1 (Consensus): There is no supporting evidence, and the medical advice is possibly based on common practice or expert opinion.<br>Score 2 (Limited): Supported by evidence from one or more low-quality studies with consistent findings, or evidence from a single moderate-quality study.<br>Score 3 (Moderate): Supported by evidence from two or more moderate-quality studies with consistent findings, or evidence from a single high-quality study.<br>Score 4 (Strong): Supported by evidence from two or more high-quality studies with consistent findings.	Strength of recommendation: 2(limited)<br>Rationale: A meta-analysis was performed using pain data from two high-quality studies (Gur 2003, Nazari 2018) and one moderate-quality (Marquina 2012) study examining high-intensity laser treatment compared to either placebo laser treatment or no treatment groups. The results of the analysis are provided in Figure 10 in the appendix. The overall findings were in favor of the laser intervention over the sham or no-treatment groups. In addition, two of these studies reported greater improvements in function (Gur 2003, Nazari 2018). Gur 2003 also compared high dose vs. low dose laser treatment on clinical outcomes and found no significant difference between the groups.<br><br>The Laser Treatment recommendation has been downgraded two levels because of feasibility, usage in practice, and a lack of internal consistency with recommendations of equal supporting evidence.
Acupuncture may improve pain and function in patients with knee osteoarthritis.	2(limited)	A meta-analysis was performed using pain data from five high-quality studies (Chen 2013, Suarez-Almazor 2010, Mavrommatis 2012, Berman 2004, Hinman 2014) and two moderate-quality studies (Vas 2007, Berman 1999). Acupuncture treatments were either traditional (Chen 2013, Hinman 2014) or electro-acupuncture (Suarez-Almazor 2010, Mavrommatis 2012, Vas 2007, Berman 2004, Berman 1999). Control groups consisted of either no acupuncture (Hinman 2014), sham acupuncture (Mavrommatis 2012, Suarez-Almazor 2010, Vas 2007, Berman 2004), sham TENS (Chen 2013), or usual care (Berman 1999). The meta-analysis also accounted for the degree of blinding effectiveness of the studies. The results of the meta-analysis can be seen in Figure 11 in the appendix. The overall findings were in favor of acupuncture for reducing pain in subjects with knee osteoarthritis. There appeared to be no effect in two studies where blinding was effective. In studies where there was no blinding or the effects of blinding were unclear, there were greater effects favoring acupuncture. This prompted our decision to apply a limited strength of recommendation in favor of acupuncture for pain control.<br><br>A similar meta-analysis was performed using the same studies for measures of function. The results of this meta-analysis can be seen in Figure 12 in the appendix. The overall findings were in favor of acupuncture for improving measures of function in subjects with knee osteoarthritis. However, the effects of blinding effectiveness on the results were similar to that described above for pain. Again, this prompted our decision to apply a limited strength of recommendation in favor of acupuncture for improving function.<br><br>Some investigators examined variations in the delivery of acupuncture treatment. Ju et al. examined high intensity vs. low intensity electro-acupuncture and found no difference between these	Consider the following medical advice:<br>Acupuncture may improve pain and function in patients with knee osteoarthritis.<br>Rate the medical advice using the following criteria, and make a selection from integer 1,2,3,4:<br>Score 1 (Consensus): There is no supporting evidence, and the medical advice is possibly based on common practice or expert opinion.<br>Score 2 (Limited): Supported by evidence from one or more low-quality studies with consistent findings, or evidence from a single moderate-quality study.<br>Score 3 (Moderate): Supported by evidence from two or more moderate-quality studies with consistent findings, or evidence from a single high-quality study.<br>Score 4 (Strong): Supported by evidence from two or more high-quality studies with consistent findings.	Strength of recommendation: 2(limited)<br>Rationale: A meta-analysis was performed using pain data from five high-quality studies (Chen 2013, Suarez-Almazor 2010, Mavrommatis 2012, Berman 2004, Hinman 2014) and two moderate-quality studies (Vas 2007, Berman 1999). Acupuncture treatments were either traditional (Chen 2013, Hinman 2014) or electro-acupuncture (Suarez-Almazor 2010, Mavrommatis 2012, Vas 2007, Berman 2004, Berman 1999). Control groups consisted of either no acupuncture (Hinman 2014), sham acupuncture (Mavrommatis 2012, Suarez-Almazor 2010, Vas 2007, Berman 2004), sham TENS (Chen 2013), or usual care (Berman 1999). The meta-analysis also accounted for the degree of blinding effectiveness of the studies. The results of the meta-analysis can be seen in Figure 11 in the appendix. The overall findings were in favor of acupuncture for reducing pain in subjects with knee osteoarthritis. There appeared to be no effect in two studies where blinding was effective. In studies where there was no blinding or the effects of blinding were unclear, there were greater effects favoring acupuncture. This prompted our decision to apply a limited strength of recommendation in favor of acupuncture for pain control.<br><br>A similar meta-analysis was performed using the same studies for measures of function. The results of this meta-analysis can be seen in Figure 12 in the appendix. The overall findings were in favor of acupuncture for improving measures of function in subjects with knee osteoarthritis. However, the effects of blinding effectiveness on the results were similar to that described above for pain. Again, this prompted our decision to apply a limited strength of recommendation in favor of acupuncture for improving function.<br><br>Some investigators examined variations in the delivery of acupuncture treatment. Ju et al. examined high intensity vs. low intensity electro-acupuncture and found no difference between these approaches for pain but possibly better improvements in function favoring the high-intensity group (Ju 2015). Others found no meaningful differences between using 2-point, 4-point, or 6-point acupuncture approaches (Qi 2016, Taechaarpornkul 2009).<br><br>The Acupuncture recommendation has been downgraded two levels because of inconsistent evidence and a lack of internal consistency with recommendations of equal supporting evidence.

		approaches for pain but possibly better improvements in function favoring the high-intensity group (Ju 2015). Others found no meaningful differences between using 2-point, 4-point, or 6-point acupuncture approaches (Qi 2016, Taechaarpornkul 2009).<br><br>The Acupuncture recommendation has been downgraded two levels because of inconsistent evidence and a lack of internal consistency with recommendations of equal supporting evidence.		
Transcutaneous Electrical Nerve Stimulation may be used to improve pain in patients with knee osteoarthritis.	2(limited)	A meta-analysis was performed using pain data from two high-quality studies (Palmer 2014, Inal 2016) and one moderate-quality study (Atamaz 2012) in which Transcutaneous Electrical Nerve Stimulation (TENS) was compared to sham TENS in subjects with knee osteoarthritis. Blinding effectiveness was considered fair in all three studies. The results of the meta-analysis can be seen in Figure 15 in the appendix. The overall findings were in favor of receiving TENS for reducing pain in subjects with knee osteoarthritis. A similar meta-analysis was performed using the same studies for measures of function. The results of this meta-analysis can be seen in Figure 16 in the appendix. The overall findings did not favor the use of TENS to improve measures of function in subjects with knee osteoarthritis.<br><br>The Transcutaneous Electrical Nerve Stimulation recommendation has been downgraded two levels because of inconsistent evidence and a lack of internal consistency with recommendations of equal supporting evidence.	Consider the following medical advice:<br>Transcutaneous Electrical Nerve Stimulation may be used to improve pain in patients with knee osteoarthritis.<br>Rate the medical advice using the following criteria, and make a selection from integer 1,2,3,4:<br>Score 1 (Consensus): There is no supporting evidence, and the medical advice is possibly based on common practice or expert opinion.<br>Score 2 (Limited): Supported by evidence from one or more low-quality studies with consistent findings, or evidence from a single moderate-quality study.<br>Score 3 (Moderate): Supported by evidence from two or more moderate-quality studies with consistent findings, or evidence from a single high-quality study.<br>Score 4 (Strong): Supported by evidence from two or more high-quality studies with consistent findings.	Strength of recommendation: 2(limited)<br>Rationale: A meta-analysis was performed using pain data from two high-quality studies (Palmer 2014, Inal 2016) and one moderate-quality study (Atamaz 2012) in which Transcutaneous Electrical Nerve Stimulation (TENS) was compared to sham TENS in subjects with knee osteoarthritis. Blinding effectiveness was considered fair in all three studies. The results of the meta-analysis can be seen in Figure 15 in the appendix. The overall findings were in favor of receiving TENS for reducing pain in subjects with knee osteoarthritis. A similar meta-analysis was performed using the same studies for measures of function. The results of this meta-analysis can be seen in Figure 16 in the appendix. The overall findings did not favor the use of TENS to improve measures of function in subjects with knee osteoarthritis.<br><br>The Transcutaneous Electrical Nerve Stimulation recommendation has been downgraded two levels because of inconsistent evidence and a lack of internal consistency with recommendations of equal supporting evidence.
Percutaneous Electrical Nerve Stimulation may be used to improve pain and function in patients with knee osteoarthritis.	2(limited)	One high-quality study was reviewed that examined the use of Percutaneous Electrical Nerve Stimulation (PENS) combined with a Cox-2 inhibitor to sham PENS combined with a Cox-2 inhibitor in subjects with knee osteoarthritis. (He 2019) The results indicated greater improvements in pain and function measures in subjects receiving PENS compared to sham PENS.<br><br>This recommendation has been downgraded one level because of feasibility issues.	Consider the following medical advice:<br>Percutaneous Electrical Nerve Stimulation may be used to improve pain and function in patients with knee osteoarthritis.<br>Rate the medical advice using the following criteria, and make a selection from integer 1,2,3,4:<br>Score 1 (Consensus): There is no supporting evidence, and the medical advice is possibly based on common practice or expert opinion.<br>Score 2 (Limited): Supported by evidence from one or more low-quality studies with consistent findings, or evidence from a single moderate-quality study.<br>Score 3 (Moderate): Supported by evidence from two or more moderate-quality studies with consistent findings, or evidence from a single high-quality study.<br>Score 4 (Strong): Supported by evidence from two or more high-quality studies with consistent findings.	Strength of recommendation: 2(limited)<br>Rationale: One high-quality study was reviewed that examined the use of Percutaneous Electrical Nerve Stimulation (PENS) combined with a Cox-2 inhibitor to sham PENS combined with a Cox-2 inhibitor in subjects with knee osteoarthritis. (He 2019) The results indicated greater improvements in pain and function measures in subjects receiving PENS compared to sham PENS.<br><br>This recommendation has been downgraded one level because of feasibility issues.
Pulsed Electromagnetic Field Therapy may be used to improve pain and function in patients with knee osteoarthritis.	2(limited)	One high-quality study was reviewed that examined the use of a wearable Pulsed Electromagnetic Field (PEMF) device for pain management in subjects with knee osteoarthritis. (Bagnato 2016) Subjects were randomized to either the PEMF group or a sham PEMF group. PEMF was applied 12 hours per day for a period of 4 weeks. The results indicated greater improvement in WOMAC pain and VAS pain scores for subjects receiving PEMF over sham PEMF. There was no difference between groups on WOMAC function scores.	Consider the following medical advice:<br>Pulsed Electromagnetic Field Therapy may be used to improve pain and function in patients with knee osteoarthritis.<br>Rate the medical advice using the following criteria, and make a selection from integer 1,2,3,4:<br>Score 1 (Consensus): There is no supporting evidence, and the medical	Strength of recommendation: 2(limited)<br>Rationale: One high-quality study was reviewed that examined the use of a wearable Pulsed Electromagnetic Field (PEMF) device for pain management in subjects with knee osteoarthritis. (Bagnato 2016) Subjects were randomized to either the PEMF group or a sham PEMF group. PEMF was applied 12 hours per day for a period of 4 weeks. The results indicated greater improvement in WOMAC pain and VAS pain scores for subjects receiving PEMF over sham PEMF. There was no difference between groups on WOMAC function scores.

		The Percutaneous Electrical Nerve Stimulation/Pulsed Electromagnetic Field Therapy recommendation has been downgraded one level because of feasibility issues.	advice is possibly based on common practice or expert opinion.<br>Score 2 (Limited): Supported by evidence from one or more low-quality studies with consistent findings, or evidence from a single moderate-quality study.<br>Score 3 (Moderate): Supported by evidence from two or more moderate-quality studies with consistent findings, or evidence from a single high-quality study.<br>Score 4 (Strong): Supported by evidence from two or more high-quality studies with consistent findings.	The Percutaneous Electrical Nerve Stimulation/Pulsed Electromagnetic Field Therapy recommendation has been downgraded one level because of feasibility issues.
Extracorporeal shockwave therapy may be used to improve pain and function for treatment of osteoarthritis of the knee.	2(limited)	A meta-analysis was performed using pain data from three high-quality studies (Zhong 2019, Ediz 2018, and Uysal 2020) in which Extracorporeal Shockwave Therapy (ESWT) was compared to sham ESWT in subjects with knee osteoarthritis. The results of the meta-analysis can be seen in Figure 19 in the appendix. The overall findings were in favor of receiving ESWT for reducing pain in subjects with knee osteoarthritis. In addition, four high-quality studies reported greater improvements in function scores in subjects receiving ESWT compared to the sham group at 4 to 12 weeks but not at 1-year follow-up. (Zhao 2013, Ediz 2018, Zhong 2019, Uysal 2020).<br><br>The Extracorporeal Shockwave Therapy recommendation has been downgraded two levels because of inconsistent evidence and a lack of internal consistency with recommendations of equal supporting evidence.	Consider the following medical advice:<br>Extracorporeal shockwave therapy may be used to improve pain and function for treatment of osteoarthritis of the knee.<br>Rate the medical advice using the following criteria, and make a selection from integer 1,2,3,4:<br>Score 1 (Consensus): There is no supporting evidence, and the medical advice is possibly based on common practice or expert opinion.<br>Score 2 (Limited): Supported by evidence from one or more low-quality studies with consistent findings, or evidence from a single moderate-quality study.<br>Score 3 (Moderate): Supported by evidence from two or more moderate-quality studies with consistent findings, or evidence from a single high-quality study.<br>Score 4 (Strong): Supported by evidence from two or more high-quality studies with consistent findings.	Strength of recommendation: 2(limited)<br>Rationale: A meta-analysis was performed using pain data from three high-quality studies (Zhong 2019, Ediz 2018, and Uysal 2020) in which Extracorporeal Shockwave Therapy (ESWT) was compared to sham ESWT in subjects with knee osteoarthritis. The results of the meta-analysis can be seen in Figure 19 in the appendix. The overall findings were in favor of receiving ESWT for reducing pain in subjects with knee osteoarthritis. In addition, four high-quality studies reported greater improvements in function scores in subjects receiving ESWT compared to the sham group at 4 to 12 weeks but not at 1-year follow-up. (Zhao 2013, Ediz 2018, Zhong 2019, Uysal 2020).<br><br>The Extracorporeal Shockwave Therapy recommendation has been downgraded two levels because of inconsistent evidence and a lack of internal consistency with recommendations of equal supporting evidence.
Oral NSAIDs are recommended to improve pain and function in the treatment of knee osteoarthritis when not contraindicated.	4(Strong)	Among the 34 high-quality, 23 moderate-quality, and 1 low-quality studies that met the inclusion criteria, non-selective and selective cyclooxygenase-2 (COX-2) oral nonsteroidal anti-inflammatory drugs (NSAIDs) consistently improved pain and function compared to controls in the treatment of osteoarthritis of the knee (Reginster 2017; Lee 2017; Gordo 2017; Strand 2017; Essex 2014; Kongtharvonskul 2016; Altman 2015; Gibofsky 2014; Ishijima 2014; Conaghan 2013; Essex 2012; Singh 2012; Elsaman 2016; Schnitzer 2011; Kivitz 2004; Fleischmann 1997; Lee 1986; Davies 1999; Sandelin 1997; Puopolo 2007; Gibofsky 2003; Bensen 1999; Kivits 2002; Clegg 2006; Sangdee 2002; Sheldon 2005; Tannenbaum 2004; Lehmann 2005; Rother 2007; Simon 2009; Svensson 2006; Schnitzer 2010; Doherty 2011; McKenna 2001 (a); Paul 2009; Bolten 2015; Essex 2015; Ekman 2014; Ohtori 2013; Selvan 2012; Pavelka 2007; Ehrich 1999; Lee 1985; Dwicandra 2018; Asmus 2014; Smugar 2006; Bingham 2007; Altman 1998; Schnitzer 1999; Birbara 2006; Williams 2001; Miceli 2004; Mckenna 2001 (b); Pincus 2004; Lohmander 2005; Schnitzer 2005b; Williams 2000; Fleischmann 2006). Although meta-analysis of non-selective oral NSAIDs compared to controls demonstrated a meaningful reduction in pain, the results need to be interpreted with caution due to the relatively high degree of heterogeneity. The meta-analysis of non-selective oral NSAIDs compared to controls demonstrated a meaningful improvement in function with an acceptable degree of heterogeneity. In terms of selective COX-2 oral NSAIDs, the meta-analysis of celecoxib, the only available selective COX-2 oral NSAID on the United States market,	Consider the following medical advice:<br>Oral NSAIDs are recommended to improve pain and function in the treatment of knee osteoarthritis when not contraindicated.<br>Rate the medical advice using the following criteria, and make a selection from integer 1,2,3,4:<br>Score 1 (Consensus): There is no supporting evidence, and the medical advice is possibly based on common practice or expert opinion.<br>Score 2 (Limited): Supported by evidence from one or more low-quality studies with consistent findings, or evidence from a single moderate-quality study.<br>Score 3 (Moderate): Supported by evidence from two or more moderate-quality studies with consistent findings, or evidence from a single high-quality study.<br>Score 4 (Strong): Supported by evidence from two or more high-quality studies with consistent findings.	Strength of recommendation: 4(Strong)<br>Rationale: Among the 34 high-quality, 23 moderate-quality, and 1 low-quality studies that met the inclusion criteria, non-selective and selective cyclooxygenase-2 (COX-2) oral nonsteroidal anti-inflammatory drugs (NSAIDs) consistently improved pain and function compared to controls in the treatment of osteoarthritis of the knee (Reginster 2017; Lee 2017; Gordo 2017; Strand 2017; Essex 2014; Kongtharvonskul 2016; Altman 2015; Gibofsky 2014; Ishijima 2014; Conaghan 2013; Essex 2012; Singh 2012; Elsaman 2016; Schnitzer 2011; Kivitz 2004; Fleischmann 1997; Lee 1986; Davies 1999; Sandelin 1997; Puopolo 2007; Gibofsky 2003; Bensen 1999; Kivits 2002; Clegg 2006; Sangdee 2002; Sheldon 2005; Tannenbaum 2004; Lehmann 2005; Rother 2007; Simon 2009; Svensson 2006; Schnitzer 2010; Doherty 2011; McKenna 2001 (a); Paul 2009; Bolten 2015; Essex 2015; Ekman 2014; Ohtori 2013; Selvan 2012; Pavelka 2007; Ehrich 1999; Lee 1985; Dwicandra 2018; Asmus 2014; Smugar 2006; Bingham 2007; Altman 1998; Schnitzer 1999; Birbara 2006; Williams 2001; Miceli 2004; Mckenna 2001 (b); Pincus 2004; Lohmander 2005; Schnitzer 2005b; Williams 2000; Fleischmann 2006). Although meta-analysis of non-selective oral NSAIDs compared to controls demonstrated a meaningful reduction in pain, the results need to be interpreted with caution due to the relatively high degree of heterogeneity. The meta-analysis of non-selective oral NSAIDs compared to controls demonstrated a meaningful improvement in function with an acceptable degree of heterogeneity. In terms of selective COX-2 oral NSAIDs, the meta-analysis of celecoxib, the only available selective COX-2 oral NSAID on the United States market, demonstrated a meaningful reduction in pain and improved function with an acceptable degree of heterogeneity. The comparison of non-selective and selective COX-2 oral NSAIDs shows no significant difference in the effectiveness between the types of oral NSAIDs (Gordo 2017; Essex 2014; Essex 2016; Essex 2012; Bensen 1999; Kivits 2002; Kivitz 2004; Puopolo 2007; Hochberg 2011; McKenna 2001 (b); Schnitzer 2005b; Malik 2017). Although NSAIDs effectively reduce pain and improve function in the treatment of osteoarthritis of the knee, providers should consider patient comorbidities, the type of NSAID administered, dose, and duration of administration. In fact, the United States Food and Drug Administration (FDA) has a black-box warning for NSAIDs citing an increased risk of serious cardiovascular thrombotic events and serious gastrointestinal events. Therefore, we recommend the lowest effective dose for the shortest duration possible for the patient. Although selective COX-2 oral

		demonstrated a meaningful reduction in pain and improved function with an acceptable degree of heterogeneity. The comparison of non-selective and selective COX-2 oral NSAIDs shows no significant difference in the effectiveness between the types of oral NSAIDs (Gordo 2017; Essex 2014; Essex 2016; Essex 2012; Bensen 1999; Kivits 2002; Kivitz 2004; Puopolo 2007; Hochberg 2011; McKenna 2001 (b); Schnitzer 2005b; Malik 2017). Although NSAIDs effectively reduce pain and improve function in the treatment of osteoarthritis of the knee, providers should consider patient comorbidities, the type of NSAID administered, dose, and duration of administration. In fact, the United States Food and Drug Administration (FDA) has a black-box warning for NSAIDs citing an increased risk of serious cardiovascular thrombotic events and serious gastrointestinal events. Therefore, we recommend the lowest effective dose for the shortest duration possible for the patient. Although selective COX-2 oral NSAIDs were developed to reduce gastrointestinal adverse events compared to non-selective oral NSAIDs, meta-analysis did not reveal a significant reduction in gastrointestinal adverse events.		NSAIDs were developed to reduce gastrointestinal adverse events compared to non-selective oral NSAIDs, meta-analysis did not reveal a significant reduction in gastrointestinal adverse events.
Oral acetaminophen is recommended to improve pain and function in the treatment of knee osteoarthritis when not contraindicated.	4(Strong)	Among the 4 high-quality and 3 moderate quality studies that met the inclusion criteria, oral acetaminophen consistently improved pain and function compared to controls in the treatment of osteoarthritis of the knee (Herrero-Beaumont 2007; Doherty 2011; Reed 2018; Prior 2014; Micelli 2004; Pincus 2004; Altman 2007). The meta-analysis of oral acetaminophen compared to controls demonstrated a meaningful reduction in pain and improved function with no evidence of confounding heterogeneity. Overall, acetaminophen is considered a safe medication with no evidence of significantly increased adverse events among the included studies. However, the United States FDA has a black-box warning for acetaminophen secondary to concern of overdose leading to hepatotoxicity or death. When oral acetaminophen was compared to NSAIDs, the use of oral NSAIDs provided a significant reduction in pain and improved function. As a result, providers may consider using oral NSAIDs instead of acetaminophen when a contraindication to oral NSAIDs does not exist in the patient.	Consider the following medical advice:<br>Oral acetaminophen is recommended to improve pain and function in the treatment of knee osteoarthritis when not contraindicated.<br>Rate the medical advice using the following criteria, and make a selection from integer 1,2,3,4:<br>Score 1 (Consensus): There is no supporting evidence, and the medical advice is possibly based on common practice or expert opinion.<br>Score 2 (Limited): Supported by evidence from one or more low-quality studies with consistent findings, or evidence from a single moderate-quality study.<br>Score 3 (Moderate): Supported by evidence from two or more moderate-quality studies with consistent findings, or evidence from a single high-quality study.<br>Score 4 (Strong): Supported by evidence from two or more high-quality studies with consistent findings.	Strength of recommendation: 4(Strong)<br>Rationale: Among the 4 high-quality and 3 moderate quality studies that met the inclusion criteria, oral acetaminophen consistently improved pain and function compared to controls in the treatment of osteoarthritis of the knee (Herrero-Beaumont 2007; Doherty 2011; Reed 2018; Prior 2014; Micelli 2004; Pincus 2004; Altman 2007). The meta-analysis of oral acetaminophen compared to controls demonstrated a meaningful reduction in pain and improved function with no evidence of confounding heterogeneity. Overall, acetaminophen is considered a safe medication with no evidence of significantly increased adverse events among the included studies. However, the United States FDA has a black-box warning for acetaminophen secondary to concern of overdose leading to hepatotoxicity or death. When oral acetaminophen was compared to NSAIDs, the use of oral NSAIDs provided a significant reduction in pain and improved function. As a result, providers may consider using oral NSAIDs instead of acetaminophen when a contraindication to oral NSAIDs does not exist in the patient.
Oral narcotics, including tramadol, result in a significant increase of adverse events and are not effective at improving pain or function for treatment of osteoarthritis of the knee.	4(Strong)	Among the 5 high-quality and 2 moderate quality studies that met the inclusion criteria, oral narcotic medications are not an effective treatment to reduce pain and improve function in osteoarthritis of the knee (Serrie 2017; Afilalo 2010; Mayorga 2016; Fishman 2007; Fleischmann 2001; Burch 2007; Babul 2004). In fact, the use of narcotics to treat osteoarthritis of the knee is consistently associated with a significantly high risk of adverse events. Due to the lack of efficacy and increase of adverse event, we would recommend against the use of narcotics for the treatment of osteoarthritis of the knee. Given the effective and relatively safe alternatives of oral NSAIDs and acetaminophen, oral narcotics should be avoided when the provider is considering the recommendation of an oral medication.	Consider the following medical advice:<br>Oral narcotics, including tramadol, result in a significant increase of adverse events and are not effective at improving pain or function for treatment of osteoarthritis of the knee.<br>Rate the medical advice using the following criteria, and make a selection from integer 1,2,3,4:<br>Score 1 (Consensus): There is no supporting evidence, and the medical advice is possibly based on common practice or expert opinion.<br>Score 2 (Limited): Supported by evidence from one or more low-quality studies with consistent findings, or evidence from a single moderate-quality study.<br>Score 3 (Moderate): Supported by evidence from two or more moderate-quality studies with consistent findings, or evidence from a single high-quality study.	Strength of recommendation: 4(Strong)<br>Rationale: Among the 5 high-quality and 2 moderate quality studies that met the inclusion criteria, oral narcotic medications are not an effective treatment to reduce pain and improve function in osteoarthritis of the knee (Serrie 2017; Afilalo 2010; Mayorga 2016; Fishman 2007; Fleischmann 2001; Burch 2007; Babul 2004). In fact, the use of narcotics to treat osteoarthritis of the knee is consistently associated with a significantly high risk of adverse events. Due to the lack of efficacy and increase of adverse event, we would recommend against the use of narcotics for the treatment of osteoarthritis of the knee. Given the effective and relatively safe alternatives of oral NSAIDs and acetaminophen, oral narcotics should be avoided when the provider is considering the recommendation of an oral medication.

			Score 4 (Strong): Supported by evidence from two or more high-quality studies with consistent findings.	
Hyaluronic acid intra-articular injection(s) is not recommended for routine use in the treatment of symptomatic osteoarthritis of the knee.	3(moderate)	Twenty-eight studies (17 high-strength (Chevalier 2010, Petterson 2018, Maheu 2019, Neustadt 2005, Baltzer 2009, Lundsgaard 2008, Altman 2004, Huang 2011, van der Weergen 2015, Altman 2009, Day 2004, Jorgensen 2010, Henrotin 2017, Henderson 1994, Hangody 2018, Saccomanno 2016, Altman 1998) and 11 moderate-strength (Jubb 2003, Navarro-Sarabia 2011, Farr 2019, Kahan 2003, Kahan 2003, Karlsson 2002, Hermans 2019, Huskisson 1999, Heybeli 2008, Petrella 2006, Takamura 2018, Wobig 1998)) assessed intraarticular hyaluronic acid (HA) injections when compared to controls. A comparison of patients from these studies and from studies validating the MCIDs were used to judge clinical significance. Results revealed that patients were demographically comparable for WOMAC and VAS pain as well as WOMAC function based on age, baseline pain scores, BMI, weight, and gender. Meta-analysis in meaningfully important difference (MID) units showed that the effect was less than 0.5 MID units, indicating a low likelihood that an appreciable number of patients achieved clinically important benefits after intraarticular HA injection (Guyatt et al.). When we differentiated high- versus low-molecular weight viscosupplementation (three high, two moderate and two low quality studies), our analyses demonstrated no significant differences among different viscosupplementation formulations. Crosslinking features of the viscosupplemtation product was assessed in two high quality studies. In patients with OA, there was no difference between cross-linked and non-cross-linked HA.<br><br>Some studies demonstrated a statistical benefit with the use of HA but could not reach the significance for a minimally clinical meaningful difference, leading to the conclusion that viscosupplementation can represent a viable option for some patients that failed other treatments when appropriately indicated. The number needed to treat to see a tangible benefit from HA was 17 patients. Furthermore, this difference was most evident at 6 weeks and 3 months. Most of the studies that exist in the literature evaluate low to moderate arthritic knees (Kellgren Lawrence of I-III) with worse results in patients with severely affected knees (KL IV).<br><br>The 2013 edition of this guideline strongly recommended against the use of viscosupplementation. In contrast to this updated version, the 2021 version found that statistically significant improvements were associated with high-molecular cross-linked hyaluronic acid but when compared to mid-range molecular weight, statistical significance was not maintained. This newer analysis did not demonstrate clinically relevant differences when compared to controls. However, as previous research reported benefits in their use, the group felt that a specific subset of patients might benefit from its use.<br><br>The Hyaluronic Acid recommendation was downgraded one level due to a lack of generalized results.	Consider the following medical advice:<br>Hyaluronic acid intra-articular injection(s) is not recommended for routine use in the treatment of symptomatic osteoarthritis of the knee.<br>Rate the medical advice using the following criteria, and make a selection from integer 1,2,3,4:<br>Score 1 (Consensus): There is no supporting evidence, and the medical advice is possibly based on common practice or expert opinion.<br>Score 2 (Limited): Supported by evidence from one or more low-quality studies with consistent findings, or evidence from a single moderate-quality study.<br>Score 3 (Moderate): Supported by evidence from two or more moderate-quality studies with consistent findings, or evidence from a single high-quality study.<br>Score 4 (Strong): Supported by evidence from two or more high-quality studies with consistent findings.	Strength of recommendation: 3(moderate)<br>Rationale: Twenty-eight studies (17 high-strength (Chevalier 2010, Petterson 2018, Maheu 2019, Neustadt 2005, Baltzer 2009, Lundsgaard 2008, Altman 2004, Huang 2011, van der Weergen 2015, Altman 2009, Day 2004, Jorgensen 2010, Henrotin 2017, Henderson 1994, Hangody 2018, Saccomanno 2016, Altman 1998) and 11 moderate-strength (Jubb 2003, Navarro-Sarabia 2011, Farr 2019, Kahan 2003, Kahan 2003, Karlsson 2002, Hermans 2019, Huskisson 1999, Heybeli 2008, Petrella 2006, Takamura 2018, Wobig 1998)) assessed intraarticular hyaluronic acid (HA) injections when compared to controls. A comparison of patients from these studies and from studies validating the MCIDs were used to judge clinical significance. Results revealed that patients were demographically comparable for WOMAC and VAS pain as well as WOMAC function based on age, baseline pain scores, BMI, weight, and gender. Meta-analysis in meaningfully important difference (MID) units showed that the effect was less than 0.5 MID units, indicating a low likelihood that an appreciable number of patients achieved clinically important benefits after intraarticular HA injection (Guyatt et al.). When we differentiated high- versus low-molecular weight viscosupplementation (three high, two moderate and two low quality studies), our analyses demonstrated no significant differences among different viscosupplementation formulations. Crosslinking features of the viscosupplemtation product was assessed in two high quality studies. In patients with OA, there was no difference between cross-linked and non-cross-linked HA.<br><br>Some studies demonstrated a statistical benefit with the use of HA but could not reach the significance for a minimally clinical meaningful difference, leading to the conclusion that viscosupplementation can represent a viable option for some patients that failed other treatments when appropriately indicated. The number needed to treat to see a tangible benefit from HA was 17 patients. Furthermore, this difference was most evident at 6 weeks and 3 months. Most of the studies that exist in the literature evaluate low to moderate arthritic knees (Kellgren Lawrence of I-III) with worse results in patients with severely affected knees (KL IV).<br><br>The 2013 edition of this guideline strongly recommended against the use of viscosupplementation. In contrast to this updated version, the 2021 version found that statistically significant improvements were associated with high-molecular cross-linked hyaluronic acid but when compared to mid-range molecular weight, statistical significance was not maintained. This newer analysis did not demonstrate clinically relevant differences when compared to controls. However, as previous research reported benefits in their use, the group felt that a specific subset of patients might benefit from its use.<br><br>The Hyaluronic Acid recommendation was downgraded one level due to a lack of generalized results.
Intra-articular corticosteroids could provide short-term relief for patients with symptomatic osteoarthritis of the knee.	3(moderate)	Our search found 18 high (Campos 2017, Cai 2019, Erturk 2016, de Campos 2013, Shrestha 2018, Mendes 2019, Yilmaz 2019, Chao 2010, Raynauld 2003, McAlindon 2017, Henrikson 2015, Neilsen 2018, Riis 2017, Arden 2014, Delgado-Enciso 2019, Smith 2003, Soriano-Maldonado 2016) and 6 moderate quality studies (Conaghan 2018, Langworthy 2019, Gaffney 1995, Yavuz 2012, Yilmaz	Consider the following medical advice:<br>Intra-articular corticosteroids could provide short-term relief for patients with symptomatic osteoarthritis of the knee.<br>Rate the medical advice using the following criteria, and make a	Strength of recommendation: 3(moderate)<br>Rationale: Our search found 18 high (Campos 2017, Cai 2019, Erturk 2016, de Campos 2013, Shrestha 2018, Mendes 2019, Yilmaz 2019, Chao 2010, Raynauld 2003, McAlindon 2017, Henrikson 2015, Neilsen 2018, Riis 2017, Arden 2014, Delgado-Enciso 2019, Smith 2003, Soriano-Maldonado 2016) and 6 moderate quality studies (Conaghan 2018, Langworthy 2019, Gaffney 1995, Yavuz 2012, Yilmaz 2019, Jones 1996) comparing intra-articular corticosteroids to control to treat knee

		2019, Jones 1996) comparing intra-articular corticosteroids to control to treat knee osteoarthritis. Overall pain and function improved with intra-articular corticosteroids; however, it is important to note that such effect lasted only up to 3 months. When we differentiated intra-articular corticosteroids extended versus immediate release (one high, two moderate quality studies) (Bodick 2015, Conaghan 2018 and Langworthy 2019), our analyses demonstrated that extended release IA steroids can be used over immediate release to improve patient outcomes (Moderate strength recommendation).<br><br>The Intra-Articular Corticosteroids recommendation has been downgraded one level because of potential risk in accelerating osteoarthritis from injections.	selection from integer 1,2,3,4:<br>Score 1 (Consensus): There is no supporting evidence, and the medical advice is possibly based on common practice or expert opinion.<br>Score 2 (Limited): Supported by evidence from one or more low-quality studies with consistent findings, or evidence from a single moderate-quality study.<br>Score 3 (Moderate): Supported by evidence from two or more moderate-quality studies with consistent findings, or evidence from a single high-quality study.<br>Score 4 (Strong): Supported by evidence from two or more high-quality studies with consistent findings.	osteoarthritis. Overall pain and function improved with intra-articular corticosteroids; however, it is important to note that such effect lasted only up to 3 months. When we differentiated intra-articular corticosteroids extended versus immediate release (one high, two moderate quality studies) (Bodick 2015, Conaghan 2018 and Langworthy 2019), our analyses demonstrated that extended release IA steroids can be used over immediate release to improve patient outcomes (Moderate strength recommendation).<br><br>The Intra-Articular Corticosteroids recommendation has been downgraded one level because of potential risk in accelerating osteoarthritis from injections.
Platelet-rich plasma (PRP) may reduce pain and improve function in patients with symptomatic osteoarthritis of the knee.	2(limited)	There were two high (Rayegani; 2014, Gormeli; 2017) and one moderate (Akan; 2018) study with 30 people per group comparing PRP vs. control. There were mixed results in the studies for pain and function. A meta-analysis was not performed due to heterogeneity. Two studies (Akan; 2018, Gormeli; 2017) looked at PRP in severe OA with mixed results. Two studies (Rayegani; 2014, Gormeli; 2017) looked at change in all stages of OA at a six-month timeframe. The studies had mixed results. One study (Gormeli; 2017) looked at Kellgren-Lawrence 1-3 stage OA with improvement in IKDC and EQ-VAS. Therefore, due to the heterogeneity of results and the difference in early and late stage OA results, we downgraded the recommendation to Limited from Strong. We feel these recommendations may change with future research on the use of PRP in different levels of severity of OA.<br><br>The number of PRP injections had mixed results with the studies with three PRP injections (Akan; 2018, Gormeli; 2017) having positive results outcomes for pain and function. Studies with one and two PRP injections had mixed results, with the positive being less likely clinically significant changes in pain and function. Further research should be done to determine the number of PRP injections for treatment of KOA. Currently, three IA-PRP injections appear to have more favorable results.<br><br>Adverse events from PRP injections have been investigated in one high-quality study (Huang; 2018) reported adverse events for PRP vs. control. They reported hypertension and proteinuria were treatment-related side-effects. These met Common Toxicity Criteria grade ≥3. This raises questions on the safety of PRP, which needs further evaluation. Therefore, the strength of recommendation was downgraded to Limited.<br><br>When evaluating the effectiveness of PRP vs HA, there were eight high-quality studies (Sanchez; 2012, Vaquerizo; 2013, Filardo; 2015, Gormeli; 2017, Cole; 2017, Buendia-Lopez; 2018, Di Martino; 2019, Yaradilmis; 2020) and six (6) medium-quality studies (Spakova; 2012, Raeissadat; 2015, Lana; 2016, Duymus; 2017, Raeissadat; 2017, Ahmad; 2018) and one low-quality study (Sanchez; 2008) that investigated IA-PRP vs. IA-HA. Four studies were included in a meta-analysis of Total WOMAC results at the 9- OR 12-months mark. This analysis showed a clinically significant difference for IA-PRP over IA-HA. The results between IA-PRP vs IA-HA diverged after 6 months.	Consider the following medical advice:<br>Platelet-rich plasma (PRP) may reduce pain and improve function in patients with symptomatic osteoarthritis of the knee.<br>Rate the medical advice using the following criteria, and make a selection from integer 1,2,3,4:<br>Score 1 (Consensus): There is no supporting evidence, and the medical advice is possibly based on common practice or expert opinion.<br>Score 2 (Limited): Supported by evidence from one or more low-quality studies with consistent findings, or evidence from a single moderate-quality study.<br>Score 3 (Moderate): Supported by evidence from two or more moderate-quality studies with consistent findings, or evidence from a single high-quality study.<br>Score 4 (Strong): Supported by evidence from two or more high-quality studies with consistent findings.	Strength of recommendation: 2(limited)<br>Rationale: There were two high (Rayegani; 2014, Gormeli; 2017) and one moderate (Akan; 2018) study with 30 people per group comparing PRP vs. control. There were mixed results in the studies for pain and function. A meta-analysis was not performed due to heterogeneity. Two studies (Akan; 2018, Gormeli; 2017) looked at PRP in severe OA with mixed results. Two studies (Rayegani; 2014, Gormeli; 2017) looked at change in all stages of OA at a six-month timeframe. The studies had mixed results. One study (Gormeli; 2017) looked at Kellgren-Lawrence 1-3 stage OA with improvement in IKDC and EQ-VAS. Therefore, due to the heterogeneity of results and the difference in early and late stage OA results, we downgraded the recommendation to Limited from Strong. We feel these recommendations may change with future research on the use of PRP in different levels of severity of OA.<br><br>The number of PRP injections had mixed results with the studies with three PRP injections (Akan; 2018, Gormeli; 2017) having positive results outcomes for pain and function. Studies with one and two PRP injections had mixed results, with the positive being less likely clinically significant changes in pain and function. Further research should be done to determine the number of PRP injections for treatment of KOA. Currently, three IA-PRP injections appear to have more favorable results.<br><br>Adverse events from PRP injections have been investigated in one high-quality study (Huang; 2018) reported adverse events for PRP vs. control. They reported hypertension and proteinuria were treatment-related side-effects. These met Common Toxicity Criteria grade ≥3. This raises questions on the safety of PRP, which needs further evaluation. Therefore, the strength of recommendation was downgraded to Limited.<br><br>When evaluating the effectiveness of PRP vs HA, there were eight high-quality studies (Sanchez; 2012, Vaquerizo; 2013, Filardo; 2015, Gormeli; 2017, Cole; 2017, Buendia-Lopez; 2018, Di Martino; 2019, Yaradilmis; 2020) and six (6) medium-quality studies (Spakova; 2012, Raeissadat; 2015, Lana; 2016, Duymus; 2017, Raeissadat; 2017, Ahmad; 2018) and one low-quality study (Sanchez; 2008) that investigated IA-PRP vs. IA-HA. Four studies were included in a meta-analysis of Total WOMAC results at the 9- OR 12-months mark. This analysis showed a clinically significant difference for IA-PRP over IA-HA. The results between IA-PRP vs IA-HA diverged after 6 months. Most studies showed similar results between IA-PRP and IA-HA at six months, except one (Yaradilimis 2020) where the LR-PRP total WOMAC was better at all time points than the IA HA. Both the patients in the IA HA and IA PRP improved in total WOMAC at six months. Patients in the IA-PRP-arms maintained improvement after 6 months at the 9- OR 12-months mark for total WOMAC vs. IA-HA which started to have a worsening score. The standard is to inject IA-HA every six months in patients with painful KOA. The preparation of the PRP (LR-PRP vs LP-PRP) was noted to be different with the LR-PRP had higher MID values than LP-PRP vs. IA-HA. The research highlights the prolonged effect of IA-PRP over IA-HA, though both appear to be equivalent at 6 months.<br>Patient-related outcome measures (OARSI-OMERACT responders, percentage of subjects meeting a percentage reduction in VAS Pain OR WOMAC Pain scores) (Sanchez; 2008, Sanchez; 2012, Vaquerizo; 2013, Buendia-Lopez; 2018) more often favored IA-PRP at both the six-month and 12-month time frame. Further research is needed using standardized PROMs to investigate the effectiveness of IA-PRP to determine if more patients will benefit from IA-PRP at six months over IA-HA.

		Most studies showed similar results between IA-PRP and IA-HA at six months, except one (Yaradilimis 2020) where the LR-PRP total WOMAC was better at all time points than the IA HA. Both the patients in the IA HA and IA PRP improved in total WOMAC at six months. Patients in the IA-PRP-arms maintained improvement after 6 months at the 9- OR 12-months mark for total WOMAC vs. IA-HA which started to have a worsening score. The standard is to inject IA-HA every six months in patients with painful KOA. The preparation of the PRP (LR-PRP vs LP-PRP) was noted to be different with the LR-PRP had higher MID values than LP-PRP vs. IA-HA. The research highlights the prolonged effect of IA-PRP over IA-HA, though both appear to be equivalent at 6 months.<br>Patient-related outcome measures (OARSI-OMERACT responders, percentage of subjects meeting a percentage reduction in VAS Pain OR WOMAC Pain scores) (Sanchez; 2008, Sanchez; 2012, Vaquerizo; 2013, Buendia-Lopez; 2018) more often favored IA-PRP at both the six-month and 12-month time frame. Further research is needed using standardized PROMs to investigate the effectiveness of IA-PRP to determine if more patients will benefit from IA-PRP at six months over IA-HA.<br><br>Adverse events were higher in the PRP group than IA HA, both local soreness and injection pain (two studies (Spakova; 2012, Yaradilmis; 2020)) and one study (Huang; 2018) reported systemic events (proteinuria and hypertension). One study (Vaquerizo; 2013) did not find a difference in comparing any adverse event, and one study (Raeissadat; 2017) did not see a difference for minor injection-site adverse events. Therefore, there appears to be more studies finding IA-PRP to have more adverse events vs. IA-HA, more research is needed to determine if the adverse events outweigh the benefit of IA-PRP over IA-HA at 9 and 12 months. This is another reason for the downgrade in evidence from Strong to Limited.<br><br>Comparisons between IA-PRP and IA-CS, there were three high (Joshi Juber; 2017, Khan; 2018, Nabi; 2018) and one moderate quality study (Huang; 2019). One study (Joshi Juber; 2017) was KL IV end-stage OA and did not find a difference. One study (Khan; 2018) was repeat injections every other month (0, 2, 4 months) with follow up at six months in KL II OAK with no difference. One study (Nabi; 2018) used patients with KL II-III given three injections one month apart showed improvement at three months (one month after the last injection) and six months (4 months after the final injection). One study (Huang; 2019) did three PRP injections every three weeks on KL I-II OAK showed improvement in pain and function at six months (4 months after last injection) and 12 months (10 months after last injection). Therefore, the IA-PRP given in three injections evaluated at 4 months post last injection is more likely to show benefit in KL II and III stages of KOA. More research is needed to evaluate long-term benefits of IA-PRP vs IA-CS over a two- or five-year period to determine if IA-PRP is cartilage sparing vs IA-CS concern for possible cartilage damage over time.<br><br>PRP is defined in LR-PRP and LP-PRP. There may be a difference in the effectiveness in knee osteoarthritis between these two preparations. Currently, there is limited data from one direct comparison (Yaradilimis; 2020) and our meta-analysis (Figure 45) of IA-PRP and IA-HA that would		Adverse events were higher in the PRP group than IA HA, both local soreness and injection pain (two studies (Spakova; 2012, Yaradilmis; 2020)) and one study (Huang; 2018) reported systemic events (proteinuria and hypertension). One study (Vaquerizo; 2013) did not find a difference in comparing any adverse event, and one study (Raeissadat; 2017) did not see a difference for minor injection-site adverse events. Therefore, there appears to be more studies finding IA-PRP to have more adverse events vs. IA-HA, more research is needed to determine if the adverse events outweigh the benefit of IA-PRP over IA-HA at 9 and 12 months. This is another reason for the downgrade in evidence from Strong to Limited.<br><br>Comparisons between IA-PRP and IA-CS, there were three high (Joshi Juber; 2017, Khan; 2018, Nabi; 2018) and one moderate quality study (Huang; 2019). One study (Joshi Juber; 2017) was KL IV end-stage OA and did not find a difference. One study (Khan; 2018) was repeat injections every other month (0, 2, 4 months) with follow up at six months in KL II OAK with no difference. One study (Nabi; 2018) used patients with KL II-III given three injections one month apart showed improvement at three months (one month after the last injection) and six months (4 months after the final injection). One study (Huang; 2019) did three PRP injections every three weeks on KL I-II OAK showed improvement in pain and function at six months (4 months after last injection) and 12 months (10 months after last injection). Therefore, the IA-PRP given in three injections evaluated at 4 months post last injection is more likely to show benefit in KL II and III stages of KOA. More research is needed to evaluate long-term benefits of IA-PRP vs IA-CS over a two- or five-year period to determine if IA-PRP is cartilage sparing vs IA-CS concern for possible cartilage damage over time.<br><br>PRP is defined in LR-PRP and LP-PRP. There may be a difference in the effectiveness in knee osteoarthritis between these two preparations. Currently, there is limited data from one direct comparison (Yaradilimis; 2020) and our meta-analysis (Figure 45) of IA-PRP and IA-HA that would demonstrate that intra-articular LR-PRP vs. LP-PRP for KOA is more likely to demonstrate a benefit at 9 and 12 months. The number of studies is limited, therefore determining the better choice between LR-PRP vs. LP-PRP is still inconclusive, but at this time appears to favor LR-PRP.<br><br>The Platelet-Rich Plasma recommendation has been downgraded two levels because of inconsistent evidence.

		demonstrate that intra-articular LR-PRP vs. LP-PRP for KOA is more likely to demonstrate a benefit at 9 and 12 months. The number of studies is limited, therefore determining the better choice between LR-PRP vs. LP-PRP is still inconclusive, but at this time appears to favor LR-PRP.<br><br>The Platelet-Rich Plasma recommendation has been downgraded two levels because of inconsistent evidence.		
Denervation therapy may reduce pain and improve function in patients with symptomatic osteoarthritis of the knee.	2(limited)	For the denervation therapies, there are 2 high-quality studies (Radnovic et al 2017 and Mendes et al 2019) and 2 moderate-quality studies (McAlindon et al 2017 and El-Hakeim et al 2018) comparing denervation technique with placebo.<br><br>One high-quality study (Radnovic et al 2017) specifically evaluated the efficacy of cryoneurolysis in comparison to placebo control in patients with knee OA. It was found that the group receiving cryoneurolysis had improved total WOMAC, WOMAC stiffness, WOMAC pain, WOMAC physical function and in VAS pain compared to the placebo control group.<br><br>Another high-quality study (Mendes et al 2019) evaluated the efficacy of chemical ablation in comparison to placebo control in patients with knee OA. It was found that the group receiving chemical denervation had improved in WOMAC pain compared to the placebo control group. Another moderate-quality study (McAlindon et al 2017) comparing the efficacy of chemical ablation in comparison to placebo control in patients with knee OA found no major difference between the two groups.<br><br>One moderate-quality study (El-Hakeim et al 2018) specifically evaluated the efficacy of thermal ablation in comparison to placebo control in patients with knee OA. It was found that the group receiving thermal ablation had improved WOMAC total, WOMAC function, and VAS pain compared to the placebo control group.<br><br>One high-quality study (Davis et al) and one moderate-evidence study (Davis et al 2018) compared IA HA to thermal ablation in patients with knee OA. The first study (Davis et al 2018) showed worse Oxford Knee Score, Global Perceived Index, and Numeric Rating Scale in the HA group compared to the thermal ablation group, while the second study (Davis et al 2018) showed worse Oxford Knee Score, Change in Medication Use (mg) from Baseline, Knee pain-Numeric Rating scale, and Mean Reduction in average NRS score in the HA group compared to the thermal ablation.<br><br>One high-quality study (Gulec et al 2017) compared unipolar to bipolar radiofrequency ablation of the knee in patients with knee OA. In patients with OA, Bipolar intra-articular pulsed radiofrequency thermocoagulation may be used over Unipolar intra-articular pulsed radiofrequency thermocoagulation to improve patient pain.<br><br>One moderate-quality study (Sari et al 2018) compared IA steroids to thermal ablation of the knee in patients with knee OA. The study showed worse WOMAC total, WOMAC function, WOMAC stiffness, and worse VAS pain in the IA steroids group compared to the thermal ablation group.	Consider the following medical advice:<br>Denervation therapy may reduce pain and improve function in patients with symptomatic osteoarthritis of the knee.<br>Rate the medical advice using the following criteria, and make a selection from integer 1,2,3,4:<br>Score 1 (Consensus): There is no supporting evidence, and the medical advice is possibly based on common practice or expert opinion.<br>Score 2 (Limited): Supported by evidence from one or more low-quality studies with consistent findings, or evidence from a single moderate-quality study.<br>Score 3 (Moderate): Supported by evidence from two or more moderate-quality studies with consistent findings, or evidence from a single high-quality study.<br>Score 4 (Strong): Supported by evidence from two or more high-quality studies with consistent findings.	Strength of recommendation: 2(limited)<br>Rationale: For the denervation therapies, there are 2 high-quality studies (Radnovic et al 2017 and Mendes et al 2019) and 2 moderate-quality studies (McAlindon et al 2017 and El-Hakeim et al 2018) comparing denervation technique with placebo.<br><br>One high-quality study (Radnovic et al 2017) specifically evaluated the efficacy of cryoneurolysis in comparison to placebo control in patients with knee OA. It was found that the group receiving cryoneurolysis had improved total WOMAC, WOMAC stiffness, WOMAC pain, WOMAC physical function and in VAS pain compared to the placebo control group.<br><br>Another high-quality study (Mendes et al 2019) evaluated the efficacy of chemical ablation in comparison to placebo control in patients with knee OA. It was found that the group receiving chemical denervation had improved in WOMAC pain compared to the placebo control group. Another moderate-quality study (McAlindon et al 2017) comparing the efficacy of chemical ablation in comparison to placebo control in patients with knee OA found no major difference between the two groups.<br><br>One moderate-quality study (El-Hakeim et al 2018) specifically evaluated the efficacy of thermal ablation in comparison to placebo control in patients with knee OA. It was found that the group receiving thermal ablation had improved WOMAC total, WOMAC function, and VAS pain compared to the placebo control group.<br><br>One high-quality study (Davis et al) and one moderate-evidence study (Davis et al 2018) compared IA HA to thermal ablation in patients with knee OA. The first study (Davis et al 2018) showed worse Oxford Knee Score, Global Perceived Index, and Numeric Rating Scale in the HA group compared to the thermal ablation group, while the second study (Davis et al 2018) showed worse Oxford Knee Score, Change in Medication Use (mg) from Baseline, Knee pain-Numeric Rating scale, and Mean Reduction in average NRS score in the HA group compared to the thermal ablation.<br><br>One high-quality study (Gulec et al 2017) compared unipolar to bipolar radiofrequency ablation of the knee in patients with knee OA. In patients with OA, Bipolar intra-articular pulsed radiofrequency thermocoagulation may be used over Unipolar intra-articular pulsed radiofrequency thermocoagulation to improve patient pain.<br><br>One moderate-quality study (Sari et al 2018) compared IA steroids to thermal ablation of the knee in patients with knee OA. The study showed worse WOMAC total, WOMAC function, WOMAC stiffness, and worse VAS pain in the IA steroids group compared to the thermal ablation group.<br><br>In summary, our analysis demonstrates that denervation therapy may reduce pain and improve function in patients with symptomatic osteoarthritis of the knee.<br><br>The Denervation Therapy recommendation has been downgraded two levels because of inconsistent evidence and bias.

		In summary, our analysis demonstrates that denervation therapy may reduce pain and improve function in patients with symptomatic osteoarthritis of the knee.<br><br>The Denervation Therapy recommendation has been downgraded two levels because of inconsistent evidence and bias.		
Arthroscopy with lavage and/or debridement in patients with a primary diagnosis of knee osteoarthritis is not recommended.	3(moderate)	There were four studies that met the inclusion criteria for this recommendation. There was one high strength (Moseley et al 2002), two moderate strength (Kirkley et al. 2008, Kalunian et al. 2000), and one low quality (Saeed et al. 2015).<br><br>Kirkely et al. 2008 compared arthroscopic surgery which included lavage and debridement combined with physical therapy and medical treatment versus physical therapy and medical treatment alone. The outcome measures utilized were the total Western Ontario and McMaster Universities Osteoarthritis Index (WOMAC) score, Short Form-36 (SF-36) Physical Component Summary score, McMaster– Toronto Arthritis Patient Preference Disability Questionnaire (MACTAR), and the Arthritis Self-Efficacy Scale (ASES) and standard-gamble utility scores. Six patients assigned to surgery elected not to have the procedure; data from these patients were analyzed, according to the intention to- treat principle, with data from the surgery group. Out of all potential outcomes, only two were statistically significant in favor of surgery. In summary, this randomized controlled trial demonstrated no benefit of arthroscopic lavage and debridement compared to physical therapy and medical treatment for osteoarthritis of the knee.<br><br>Kalunian et al. 2000 compared arthroscopic lavage (3000ml) with placebo (250ml). The study was performed at 4 different institutes and included a large number of enrolled patients from one institution with intra-articular crystals in their knee. The arthroscopes used were less than usual caliber in size ranging from 1.7mm to 2.7mm. Outcome measures were WOMAC scores at 12 months. There were not any statistically significant differences in aggregate WOMAC scores between the two treatment groups. The study concludes that irrigation may be helpful in a small subset of patients, especially those with crystals.<br><br>Mosley et al. 2002 study is an RCT comparing arthroscopic debridement, arthroscopic lavage, versus placebo / sham surgery. The study provides strong evidence that knee arthroscopy with or without debridement is not better and appears to be equivalent to a placebo procedure in improving knee pain and self-reported function. However, the study raised questions regarding its limited sampling (mostly male veterans) as well as the number of potential study participants who declined randomization into a treatment group. They also used a non-validated Knee Specific Pain score. Also, patients with substantial malalignment (varus or valgus deformity) and those with advanced disease, who might have a poorer response to surgical intervention were included in the trial.<br><br>Saeed et al. 2015 compared HA injections versus arthroscopic debridement in patients with OA in an RCT where only the pain component of the knee society score was utilized. In the short-term follow-up of 6 months, arthroscopy failed to show better pain outcome than injections.	Consider the following medical advice:<br>Arthroscopy with lavage and/or debridement in patients with a primary diagnosis of knee osteoarthritis is not recommended.<br>Rate the medical advice using the following criteria, and make a selection from integer 1,2,3,4:<br>Score 1 (Consensus): There is no supporting evidence, and the medical advice is possibly based on common practice or expert opinion.<br>Score 2 (Limited): Supported by evidence from one or more low-quality studies with consistent findings, or evidence from a single moderate-quality study.<br>Score 3 (Moderate): Supported by evidence from two or more moderate-quality studies with consistent findings, or evidence from a single high-quality study.<br>Score 4 (Strong): Supported by evidence from two or more high-quality studies with consistent findings.	Strength of recommendation: 3(moderate)<br>Rationale: There were four studies that met the inclusion criteria for this recommendation. There was one high strength (Moseley et al 2002), two moderate strength (Kirkley et al. 2008, Kalunian et al. 2000), and one low quality (Saeed et al. 2015).<br><br>Kirkely et al. 2008 compared arthroscopic surgery which included lavage and debridement combined with physical therapy and medical treatment versus physical therapy and medical treatment alone. The outcome measures utilized were the total Western Ontario and McMaster Universities Osteoarthritis Index (WOMAC) score, Short Form-36 (SF-36) Physical Component Summary score, McMaster– Toronto Arthritis Patient Preference Disability Questionnaire (MACTAR), and the Arthritis Self-Efficacy Scale (ASES) and standard-gamble utility scores. Six patients assigned to surgery elected not to have the procedure; data from these patients were analyzed, according to the intention to- treat principle, with data from the surgery group. Out of all potential outcomes, only two were statistically significant in favor of surgery. In summary, this randomized controlled trial demonstrated no benefit of arthroscopic lavage and debridement compared to physical therapy and medical treatment for osteoarthritis of the knee.<br><br>Kalunian et al. 2000 compared arthroscopic lavage (3000ml) with placebo (250ml). The study was performed at 4 different institutes and included a large number of enrolled patients from one institution with intra-articular crystals in their knee. The arthroscopes used were less than usual caliber in size ranging from 1.7mm to 2.7mm. Outcome measures were WOMAC scores at 12 months. There were not any statistically significant differences in aggregate WOMAC scores between the two treatment groups. The study concludes that irrigation may be helpful in a small subset of patients, especially those with crystals.<br><br>Mosley et al. 2002 study is an RCT comparing arthroscopic debridement, arthroscopic lavage, versus placebo / sham surgery. The study provides strong evidence that knee arthroscopy with or without debridement is not better and appears to be equivalent to a placebo procedure in improving knee pain and self-reported function. However, the study raised questions regarding its limited sampling (mostly male veterans) as well as the number of potential study participants who declined randomization into a treatment group. They also used a non-validated Knee Specific Pain score. Also, patients with substantial malalignment (varus or valgus deformity) and those with advanced disease, who might have a poorer response to surgical intervention were included in the trial.<br><br>Saeed et al. 2015 compared HA injections versus arthroscopic debridement in patients with OA in an RCT where only the pain component of the knee society score was utilized. In the short-term follow-up of 6 months, arthroscopy failed to show better pain outcome than injections.<br><br>Most of the studies excluded patients with meniscal tear, loose body, or other mechanical derangement, with concomitant diagnosis of osteoarthritis of the knee. The present recommendation does not apply to such patients.

		Most of the studies excluded patients with meniscal tear, loose body, or other mechanical derangement, with concomitant diagnosis of osteoarthritis of the knee. The present recommendation does not apply to such patients.		
Arthroscopic partial meniscectomy can be used for the treatment of meniscal tears in patients with concomitant mild to moderate osteoarthritis who have failed physical therapy or other nonsurgical treatments.	3(moderate)	The three studies discussed below compare outcomes following arthroscopic partial meniscectomy with physical therapy and demonstrate that knee arthroscopy with partial meniscectomy is as effective as physical therapy. In PICO 5, this workgroup recommended supervised or unsupervised exercise as opposed to no exercise to improve pain and function in patients with knee osteoarthritis. Currently, there are no studies that compare outcomes (knee pain and function) following arthroscopic partial meniscectomy versus physical therapy alone in patients who have failed to improve with an initial course of physical therapy. It is important to clearly define the appropriate indications for arthroscopic partial meniscectomy in patients with knee OA. This procedure should be considered in patients with mild-to-moderate knee OA and an MRI-confirmed meniscal tear who have previously failed appropriate conservative treatment such as physical therapy, corticosteroid injections, and a course of non-steroidal anti-inflammatory medications.<br><br>Katz et al (2013) conducted a multicenter, randomized, controlled trial of symptomatic patients over the age 45 or older with a meniscal tear and evidence of mild-to-moderate knee osteoarthritis to determine the efficacy of arthroscopic partial meniscectomy compared to standardized physical therapy in this patient population. Three hundred fifty-one patients were randomly assigned to surgery and postoperative physical therapy or to a standardized physical therapy regimen (with the option to cross over to surgery at the discretion of the patient and surgeon). The patients were evaluated at 6 and 12 months, and the primary outcome was the difference between the groups with respect to the change in the Western Ontario and McMaster Universities Osteoarthritis Index (WOMAC) physical function score. The mean improvement in WOMAC score at 6 months was similar between the groups. At 6 months, 51 patients who were randomized to physical therapy alone (30%) had undergone surgery. The authors concluded that in their intention-to-treat analysis, there were no significant differences in functional improvement 6 months after randomization; however, 30% of patients in the physical therapy alone group underwent surgery. These patients were analyzed in their original group, based on the intention-to-treat analysis.<br><br>Van de Graaf et al. (2018) performed a multicenter randomized clinical trial in the Netherlands to determine whether physical therapy is inferior to arthroscopic partial meniscectomy (APM) for improving patient-reported knee function in patients with meniscal tears. Three hundred twenty-one patients were randomly assigned to APM or a predefined physical therapy protocol. Patients were excluded if they had locking of the knee, prior knee surgery, instability caused by anterior or posterior cruciate ligament rupture, severe osteoarthritis (Kellgren Lawrence score of 4), and a BMI > 35 kg/m2. Change in patient-reported knee function on the International Knee Documentation Committee Subscale Knee form (IKDC) over a 24-month period was used as the primary outcome. In the PT group, 47 patients (29%) had APM during the 24-month follow-up period. The authors noted a similar level of improvement in knee function between the APM and PT groups. They concluded that PT was noninferior to APM for improving patient-reported knee	Consider the following medical advice:<br>Arthroscopic partial meniscectomy can be used for the treatment of meniscal tears in patients with concomitant mild to moderate osteoarthritis who have failed physical therapy or other nonsurgical treatments.<br>Rate the medical advice using the following criteria, and make a selection from integer 1,2,3,4:<br>Score 1 (Consensus): There is no supporting evidence, and the medical advice is possibly based on common practice or expert opinion.<br>Score 2 (Limited): Supported by evidence from one or more low-quality studies with consistent findings, or evidence from a single moderate-quality study.<br>Score 3 (Moderate): Supported by evidence from two or more moderate-quality studies with consistent findings, or evidence from a single high-quality study.<br>Score 4 (Strong): Supported by evidence from two or more high-quality studies with consistent findings.	Strength of recommendation: 3(moderate)<br>Rationale: The three studies discussed below compare outcomes following arthroscopic partial meniscectomy with physical therapy and demonstrate that knee arthroscopy with partial meniscectomy is as effective as physical therapy. In PICO 5, this workgroup recommended supervised or unsupervised exercise as opposed to no exercise to improve pain and function in patients with knee osteoarthritis. Currently, there are no studies that compare outcomes (knee pain and function) following arthroscopic partial meniscectomy versus physical therapy alone in patients who have failed to improve with an initial course of physical therapy. It is important to clearly define the appropriate indications for arthroscopic partial meniscectomy in patients with knee OA. This procedure should be considered in patients with mild-to-moderate knee OA and an MRI-confirmed meniscal tear who have previously failed appropriate conservative treatment such as physical therapy, corticosteroid injections, and a course of non-steroidal anti-inflammatory medications.<br><br>Katz et al (2013) conducted a multicenter, randomized, controlled trial of symptomatic patients over the age 45 or older with a meniscal tear and evidence of mild-to-moderate knee osteoarthritis to determine the efficacy of arthroscopic partial meniscectomy compared to standardized physical therapy in this patient population. Three hundred fifty-one patients were randomly assigned to surgery and postoperative physical therapy or to a standardized physical therapy regimen (with the option to cross over to surgery at the discretion of the patient and surgeon). The patients were evaluated at 6 and 12 months, and the primary outcome was the difference between the groups with respect to the change in the Western Ontario and McMaster Universities Osteoarthritis Index (WOMAC) physical function score. The mean improvement in WOMAC score at 6 months was similar between the groups. At 6 months, 51 patients who were randomized to physical therapy alone (30%) had undergone surgery. The authors concluded that in their intention-to-treat analysis, there were no significant differences in functional improvement 6 months after randomization; however, 30% of patients in the physical therapy alone group underwent surgery. These patients were analyzed in their original group, based on the intention-to-treat analysis.<br><br>Van de Graaf et al. (2018) performed a multicenter randomized clinical trial in the Netherlands to determine whether physical therapy is inferior to arthroscopic partial meniscectomy (APM) for improving patient-reported knee function in patients with meniscal tears. Three hundred twenty-one patients were randomly assigned to APM or a predefined physical therapy protocol. Patients were excluded if they had locking of the knee, prior knee surgery, instability caused by anterior or posterior cruciate ligament rupture, severe osteoarthritis (Kellgren Lawrence score of 4), and a BMI > 35 kg/m2. Change in patient-reported knee function on the International Knee Documentation Committee Subscale Knee form (IKDC) over a 24-month period was used as the primary outcome. In the PT group, 47 patients (29%) had APM during the 24-month follow-up period. The authors noted a similar level of improvement in knee function between the APM and PT groups. They concluded that PT was noninferior to APM for improving patient-reported knee function over a 24-month follow-up period in patients with nonobstructive meniscal tears.<br><br>In 2007, Herrlin et al. performed a prospective randomized study to compare knee function and physical activity following arthroscopic partial meniscectomy followed by supervised exercise or supervised exercise alone in patients with non-traumatic medial meniscal tear. Ninety patients were evaluated using the Knee Injury and Osteoarthritis Outcomes Score (KOOS), the Lysholm Knee Scoring Scale, and Tegner Activity Scale and a Visual Analog Scale (VAS) for pain prior to the intervention and after 8 weeks of exercise and 6 months following intervention. The authors found that after the intervention, both groups reported decreased knee pain, improved knee function, and high satisfaction (p < 0.0001). They, therefore, concluded that arthroscopic partial meniscectomy was not superior to supervised exercise alone in terms of reduced knee pain, improved knee function, and improved quality of life.

		function over a 24-month follow-up period in patients with nonobstructive meniscal tears.<br><br>In 2007, Herrlin et al. performed a prospective randomized study to compare knee function and physical activity following arthroscopic partial meniscectomy followed by supervised exercise or supervised exercise alone in patients with non-traumatic medial meniscal tear. Ninety patients were evaluated using the Knee Injury and Osteoarthritis Outcomes Score (KOOS), the Lysholm Knee Scoring Scale, and Tegner Activity Scale and a Visual Analog Scale (VAS) for pain prior to the intervention and after 8 weeks of exercise and 6 months following intervention. The authors found that after the intervention, both groups reported decreased knee pain, improved knee function, and high satisfaction ($p < 0.0001$). They, therefore, concluded that arthroscopic partial meniscectomy was not superior to supervised exercise alone in terms of reduced knee pain, improved knee function, and improved quality of life.		
High tibial osteotomy may be considered to improve pain and function in properly indicated patients with unicompartmental knee osteoarthritis.	2(limited)	High tibial osteotomy (HTO) has been used for pain relief of medial compartment knee osteoarthritis. Realigning the varus knee provides mechanical decompression of the medial compartment. An osteotomy line is created in the proximal tibial, and either a wedge defect is created by opening the medial cortex and held open with a wedge or plate and screw hardware, or a lateral wedge is removed and secured commonly with staples or wires. In the Nerhus 2017 study, patients continued to show improvement 6 and 12 months post-operatively. Historical studies have reported pain reduction with survival rates approximately 70% at 10 years ("survival" usually interpreted with endpoint conversion to replacement) (van Outeren cites Brouwer 2014 and Niinimaki 2012).<br><br>Many studies available for review by the workgroup compared various techniques of osteotomy in randomized studies. Ogawa 2019 found osteotomy distal to the tibial tubercle to be superior to proximal osteotomy for an opening wedge procedure with regard to patellofemoral pain. Arthroscopic evaluation of the knee joint at the time of osteotomy and second look at the time of hardware removal showed less patellar and trochlear degeneration with the distal osteotomy group.<br><br>Nerhus 2017 saw no significant functional difference between surgical patients randomized to either opening or closing wedge, with all showing improvement.<br><br>Duivenvoorden 2014 reported improved HSS scores from 71 to 81 at 6 years post-op. VAS scores improved from 6.1 baseline to a statistically significant difference at follow up of 4.0 in the opening wedge patients and 3.2 with the closing wedge (albeit no statistical difference between the groups). It should be noted that patients lost to follow up started with a VAS score of 6.6, thus tempering the analysis of late results.<br><br>Brouwer 2006 performed a prospective randomized trial comparing closing wedge and opening wedge techniques. Closing wedge was secured with two surgical staples and opening wedge with a Puddu plate. After one year, VAS score had improved from 6.1 to 3.6.<br><br>Van Outeren 2017 is perhaps the closest attempt to a large randomized control trial between surgery	Consider the following medical advice:<br>High tibial osteotomy may be considered to improve pain and function in properly indicated patients with unicompartmental knee osteoarthritis.<br>Rate the medical advice using the following criteria, and make a selection from integer 1,2,3,4:<br>Score 1 (Consensus): There is no supporting evidence, and the medical advice is possibly based on common practice or expert opinion.<br>Score 2 (Limited): Supported by evidence from one or more low-quality studies with consistent findings, or evidence from a single moderate-quality study.<br>Score 3 (Moderate): Supported by evidence from two or more moderate-quality studies with consistent findings, or evidence from a single high-quality study.<br>Score 4 (Strong): Supported by evidence from two or more high-quality studies with consistent findings.	Strength of recommendation: 2(limited)<br>Rationale: High tibial osteotomy (HTO) has been used for pain relief of medial compartment knee osteoarthritis. Realigning the varus knee provides mechanical decompression of the medial compartment. An osteotomy line is created in the proximal tibial, and either a wedge defect is created by opening the medial cortex and held open with a wedge or plate and screw hardware, or a lateral wedge is removed and secured commonly with staples or wires. In the Nerhus 2017 study, patients continued to show improvement 6 and 12 months post-operatively. Historical studies have reported pain reduction with survival rates approximately 70% at 10 years ("survival" usually interpreted with endpoint conversion to replacement) (van Outeren cites Brouwer 2014 and Niinimaki 2012).<br><br>Many studies available for review by the workgroup compared various techniques of osteotomy in randomized studies. Ogawa 2019 found osteotomy distal to the tibial tubercle to be superior to proximal osteotomy for an opening wedge procedure with regard to patellofemoral pain. Arthroscopic evaluation of the knee joint at the time of osteotomy and second look at the time of hardware removal showed less patellar and trochlear degeneration with the distal osteotomy group.<br><br>Nerhus 2017 saw no significant functional difference between surgical patients randomized to either opening or closing wedge, with all showing improvement.<br><br>Duivenvoorden 2014 reported improved HSS scores from 71 to 81 at 6 years post-op. VAS scores improved from 6.1 baseline to a statistically significant difference at follow up of 4.0 in the opening wedge patients and 3.2 with the closing wedge (albeit no statistical difference between the groups). It should be noted that patients lost to follow up started with a VAS score of 6.6, thus tempering the analysis of late results.<br><br>Brouwer 2006 performed a prospective randomized trial comparing closing wedge and opening wedge techniques. Closing wedge was secured with two surgical staples and opening wedge with a Puddu plate. After one year, VAS score had improved from 6.1 to 3.6.<br><br>Van Outeren 2017 is perhaps the closest attempt to a large randomized control trial between surgery and non-operative management. However, this is still not a highest quality randomized control trial. The researchers gathered two different groups of patients at two different hospitals. The first group underwent randomization to valgus bracing versus usual care. The second group was randomized to HTO with either an opening wedge or closing wedge osteotomy. The groups were matched for baseline characteristics. They found HTO more effective in pain reduction compared to non-operative methods. VAS changed from baseline 6.2 in the surgery group to 3.8 post-op. The control group improved from 6.4 to 5.0. Function was improved only in comparison of surgical patients to usual care treatment.<br><br>The Wu 2017 study evaluated people with bilateral OA with pain around the medial part of the knee. The more degenerative knee got proximal tibial osteotomy, and the other knee got usual non-operative care. The study authors included a table of individual patient data, which allowed a model that controlled for differences in baseline knee society function scores between the knees to be run. With this model, the odds ratio of achieving satisfactory knee society function scores (defined as score >= 80) with osteotomy vs. non-operative treatment was 7.51 (CI 1.094, 51.6).

		and non-operative management. However, this is still not a highest quality randomized control trial. The researchers gathered two different groups of patients at two different hospitals. The first group underwent randomization to valgus bracing versus usual care. The second group was randomized to HTO with either an opening wedge or closing wedge osteotomy. The groups were matched for baseline characteristics. They found HTO more effective in pain reduction compared to non-operative methods. VAS changed from baseline 6.2 in the surgery group to 3.8 post-op. The control group improved from 6.4 to 5.0. Function was improved only in comparison of surgical patients to usual care treatment.<br><br>The Wu 2017 study evaluated people with bilateral OA with pain around the medial part of the knee. The more degenerative knee got proximal tibial osteotomy, and the other knee got usual non-operative care. The study authors included a table of individual patient data, which allowed a model that controlled for differences in baseline knee society function scores between the knees to be run. With this model, the odds ratio of achieving satisfactory knee society function scores (defined as score >= 80) with osteotomy vs. non-operative treatment was 7.51 (CI 1.094, 51.6).<br><br>The Tibial Osteotomy recommendation has been downgraded one level because of inconsistent evidence.		The Tibial Osteotomy recommendation has been downgraded one level because of inconsistent evidence.
Dry needling may be used to improve pain and function in patients with knee osteoarthritis.	1(consensus)	Two high-quality studies examining the effectiveness of dry needling in combination with either exercise versus sham dry needling and exercise (Sanchez, 2019) or dry needling combined with manual therapy and exercise versus manual therapy and exercise alone (Dunning, 2018) were reviewed. Sanchez et al. 2019 found no difference in clinical outcomes of pain or function between treatment groups. In contrast, Dunning et al. found greater improvements in measures of pain and function in the group receiving dry needling. The inconsistency in the results of these studies has prompted the workgroup not to make a recommendation for or against dry needling at this time. Additional evidence will be required before a recommendation can be made.	Consider the following medical advice:<br>Dry needling may be used to improve pain and function in patients with knee osteoarthritis.<br>Rate the medical advice using the following criteria, and make a selection from integer 1,2,3,4:<br>Score 1 (Consensus): There is no supporting evidence, and the medical advice is possibly based on common practice or expert opinion.<br>Score 2 (Limited): Supported by evidence from one or more low-quality studies with consistent findings, or evidence from a single moderate-quality study.<br>Score 3 (Moderate): Supported by evidence from two or more moderate-quality studies with consistent findings, or evidence from a single high-quality study.<br>Score 4 (Strong): Supported by evidence from two or more high-quality studies with consistent findings.	Strength of recommendation: 1(consensus)<br>Rationale: Two high-quality studies examining the effectiveness of dry needling in combination with either exercise versus sham dry needling and exercise (Sanchez, 2019) or dry needling combined with manual therapy and exercise versus manual therapy and exercise alone (Dunning, 2018) were reviewed. Sanchez et al. 2019 found no difference in clinical outcomes of pain or function between treatment groups. In contrast, Dunning et al. found greater improvements in measures of pain and function in the group receiving dry needling. The inconsistency in the results of these studies has prompted the workgroup not to make a recommendation for or against dry needling at this time. Additional evidence will be required before a recommendation can be made.
Free-floating (un-fixed) interpositional devices should not be used in patients with symptomatic medial compartment osteoarthritis of the knee.	1(consensus)	One study met inclusion criteria, and no additional studies were available for review since the prior edition OAK CPG was published. The single study was a case series and retrospective review of outcomes in patients receiving the surgical intervention for isolated medial compartment OA. The study indicated high reoperation rates in the patients who were followed, with 32% of patients being revised to total knee arthroplasty during the study period. Regarding pain and functional improvement, the study reported no statistical difference in preoperative and postoperative Knee Society Scores. Given the lack of evidence to support use, the AAOS workgroup modified the grade of this recommendation to consensus, because of the high revision rates in this study, and the potential harm associated with surgical intervention (anesthesia risks, VTE, infection, and	Consider the following medical advice:<br>Free-floating (un-fixed) interpositional devices should not be used in patients with symptomatic medial compartment osteoarthritis of the knee.<br>Rate the medical advice using the following criteria, and make a selection from integer 1,2,3,4:<br>Score 1 (Consensus): There is no supporting evidence, and the medical advice is possibly based on common practice or expert opinion.<br>Score 2 (Limited): Supported by evidence from one or more	Strength of recommendation: 1(consensus)<br>Rationale: One study met inclusion criteria, and no additional studies were available for review since the prior edition OAK CPG was published. The single study was a case series and retrospective review of outcomes in patients receiving the surgical intervention for isolated medial compartment OA. The study indicated high reoperation rates in the patients who were followed, with 32% of patients being revised to total knee arthroplasty during the study period. Regarding pain and functional improvement, the study reported no statistical difference in preoperative and postoperative Knee Society Scores. Given the lack of evidence to support use, the AAOS workgroup modified the grade of this recommendation to consensus, because of the high revision rates in this study, and the potential harm associated with surgical intervention (anesthesia risks, VTE, infection, and reoperation).

		reoperation).	low-quality studies with consistent findings, or evidence from a single moderate-quality study.<br>Score 3 (Moderate): Supported by evidence from two or more moderate-quality studies with consistent findings, or evidence from a single high-quality study.<br>Score 4 (Strong): Supported by evidence from two or more high-quality studies with consistent findings.	

Supplementary Table 7. Extracted advice.

Advice	Strength of the recommendation
Lateral wedge insoles are not recommended for patients with knee osteoarthritis.	4(Strong)
Canes could be used to improve pain and function in patients with knee osteoarthritis.	3(moderate)
Brace treatment could be used to improve function, pain, and quality of life in patients with knee osteoarthritis.	3(moderate)
Turmeric may be helpful in reducing pain and improving function for patients with mild to moderate knee osteoarthritis.	2(limited)
Ginger extract may be helpful in reducing pain and improving function for patients with mild to moderate knee osteoarthritis.	2(limited)
Glucosamine may be helpful in reducing pain and improving function for patients with mild to moderate knee osteoarthritis.	2(limited)
Chondroitin may be helpful in reducing pain and improving function for patients with mild to moderate knee osteoarthritis.	2(limited)
Vitamin D may be helpful in reducing pain and improving function for patients with mild to moderate knee osteoarthritis.	2(limited)
Topical NSAIDs should be used to improve function and quality of life for treatment of osteoarthritis of the knee, when not contraindicated.	4(Strong)
Supervised exercise, unsupervised exercise, and/or aquatic exercise are recommended over no exercise to improve pain and function for treatment of knee osteoarthritis.	4(Strong)
Neuromuscular training (i.e. balance, agility, coordination) programs in combination with traditional exercise could be used to improve performance-based function and walking speed for treatment of knee osteoarthritis.	3(moderate)
Self- management programs are recommended to improve pain and function for patients with knee osteoarthritis.	4(Strong)
Patient education programs are recommended to improve pain in patients with knee osteoarthritis.	4(Strong)
Sustained weight loss is recommended to improve pain and function in overweight and obese patients with knee osteoarthritis.	3(moderate)
Manual therapy in addition to an exercise program may be used to improve pain and function in patients with knee osteoarthritis.	2(limited)
Massage may be used in addition to usual care to improve pain and function in patients with knee osteoarthritis.	2(limited)
FDA-approved laser treatment may be used to improve pain and function in patients with knee osteoarthritis.	2(limited)
Acupuncture may improve pain and function in patients with knee osteoarthritis.	2(limited)
Transcutaneous Electrical Nerve Stimulation may be used to improve pain in patients with knee osteoarthritis.	2(limited)
Percutaneous Electrical Nerve Stimulation may be used to improve pain and function in patients with knee osteoarthritis.	2(limited)
Pulsed Electromagnetic Field Therapy may be used to improve pain and function in patients	2(limited)

with knee osteoarthritis.	
Extracorporeal shockwave therapy may be used to improve pain and function for treatment of osteoarthritis of the knee.	2(limited)
Oral NSAIDs are recommended to improve pain and function in the treatment of knee osteoarthritis when not contraindicated.	4(Strong)
Oral acetaminophen is recommended to improve pain and function in the treatment of knee osteoarthritis when not contraindicated.	4(Strong)
Oral narcotics, including tramadol, result in a significant increase of adverse events and are not effective at improving pain or function for treatment of osteoarthritis of the knee.	4(Strong)
Hyaluronic acid intra-articular injection(s) is not recommended for routine use in the treatment of symptomatic osteoarthritis of the knee.	3(moderate)
Intra-articular corticosteroids could provide short-term relief for patients with symptomatic osteoarthritis of the knee.	3(moderate)
Platelet-rich plasma (PRP) may reduce pain and improve function in patients with symptomatic osteoarthritis of the knee.	2(limited)
Denervation therapy may reduce pain and improve function in patients with symptomatic osteoarthritis of the knee.	2(limited)
Arthroscopy with lavage and/or debridement in patients with a primary diagnosis of knee osteoarthritis is not recommended.	3(moderate)
Arthroscopic partial meniscectomy can be used for the treatment of meniscal tears in patients with concomitant mild to moderate osteoarthritis who have failed physical therapy or other nonsurgical treatments.	3(moderate)
High tibial osteotomy may be considered to improve pain and function in properly indicated patients with unicompartmental knee osteoarthritis.	2(limited)
Dry needling may be used to improve pain and function in patients with knee osteoarthritis.	1(consensus)
Free-floating (un-fixed) interpositional devices should not be used in patients with symptomatic medial compartment osteoarthritis of the knee.	1(consensus)

Supplementary Note 1. An example of the ROT prompting and corresponding answer in gpt-4-Web.

User

Consider the following medical advice:

Lateral wedge insoles are not recommended for patients with knee osteoarthritis.

Rate the medical advice using the following criteria, and make a selection from integer 1,2,3,4:

Score 1 (Consensus): There is no supporting evidence, and the medical advice is possibly based on common practice or expert opinion.

Score 2 (Limited): Supported by evidence from one or more low-quality studies with consistent findings, or evidence from a single moderate-quality study.

Score 3 (Moderate): Supported by evidence from two or more moderate-quality studies with consistent findings, or evidence from a single high-quality study.

Score 4 (Strong): Supported by evidence from two or more high-quality studies with consistent findings.

Imagine three different experts (Expert A, Expert B, Expert C) are completing the task above step by step:

Step 1: Each expert independently understands the medical advice without knowing others' thoughts.

Step 2: Each expert independently gathers evidence up until your knowledge cut-off in September 2021 without knowing others' thoughts.

Step 3: Experts share all the gathered evidence.

Step 4: Each expert independently evaluates the quality and quantity of evidence gathered by all experts without knowing others' thoughts.

Step 5: Each expert independently evaluates the consistency of evidence gathered by all experts without knowing others' thoughts.

Step 6: Each expert independently rates the medical advice as the following format without knowing others' thoughts:

Score: <Insert an integer>

Reasons: <Explain the reasons for the score given>

Step 7: Experts discuss together, and experts should be open to reconsidering their initial evaluations based on the shared evidence and others' thoughts.

Step 8: After discussing, each expert rates the medical advice again as the following format:

Score: <Insert an integer>

Reasons: <Explain the reasons for the score given>

Step 9: Experts reach a final answer as the following format:

Score: <Insert an integer>

Reasons: <Explain the reasons for the score given>

I must emphasize that in Step 1, 2, 4, 5, and 6, experts absolutely should not know others' thoughts.

Show your work of each step.

ChatGPT

Sure, I'll simulate the actions and thoughts of three experts as they evaluate this medical advice.

Step 1: Understanding the medical advice

Expert A: Understands that the advice is not recommending the use of lateral wedge insoles for patients with knee osteoarthritis.

Expert B: Understands the advice is suggesting that lateral wedge insoles might not be beneficial for knee osteoarthritis patients.

Expert C: Understands that the advice is stating that knee osteoarthritis patients should not use lateral wedge insoles.

Step 2: Gathering evidence (as of my knowledge cut-off in September 2021)

Expert A: Finds a systematic review and meta-analysis from 2018 showing no significant difference in pain or function with the use of lateral wedge insoles for knee osteoarthritis.

Expert B: Finds a 2019 study which shows limited improvement with lateral wedge insoles but indicates further research is needed.

Expert C: Finds a 2020 study which suggests that while lateral wedge insoles may not show significant improvement for some patients, they could still be considered as an option depending on individual patient factors.

Step 3: Sharing all the gathered evidence

All experts share the studies they found.

Step 4: Evaluating the quality and quantity of evidence

Expert A: Evaluates the systematic review and meta-analysis as high quality, and the other studies as moderate quality.

Expert B: Evaluates the systematic review and meta-analysis as high quality, and the other studies as moderate quality.

Expert C: Evaluates the systematic review and meta-analysis as high quality, and the other studies as moderate quality.

Step 5: Evaluating the consistency of evidence

Expert A: Sees some inconsistencies in the results, but the overall trend suggests limited to no benefit from lateral wedge insoles for knee osteoarthritis.

Expert B: Notes that the studies show a range of results, from no significant benefit to possible benefit depending on individual factors.

Expert C: Observes that the results are somewhat mixed, but there's a general agreement that the benefits, if any, from lateral wedge insoles are limited.

Step 6: Initial rating of medical advice

Expert A: Score: 3. Reasons: There is a high-quality systematic review and meta-analysis suggesting limited to no benefit of lateral wedge insoles, along with other moderate-quality studies showing similar findings.

Expert B: Score: 2. Reasons: There are moderate-quality studies suggesting a possible benefit depending on individual factors, but a high-quality systematic review showing limited to no benefit.

Expert C: Score: 2. Reasons: While some moderate-quality studies suggest potential benefits for certain individuals, a high-quality systematic review indicates limited to no benefit.

Step 7: Experts discuss together

The experts discuss the studies and acknowledge that there's a trend towards limited to no benefit from lateral wedge insoles for knee osteoarthritis. However, they also note that some studies suggest potential benefit for certain individuals.

Step 8: After discussing, each expert rates the medical advice again

Expert A: Score: 3. Reasons: The high-quality systematic review and consistent findings from moderate-quality studies suggest limited to no benefit from lateral wedge insoles.

Expert B: Score: 3. Reasons: Despite potential individual benefits suggested by some studies, the high-quality systematic review and overall trend from moderate-quality studies point towards limited to no benefit.

Expert C: Score: 3. Reasons: While the benefits might be subject to individual factors, the overall evidence, including a high-quality systematic review, indicates limited to no benefit.

Step 9: Experts reach a final answer

Score: 3. Reasons: Based on a high-quality systematic review and consistent findings from moderate-quality studies, there's limited to no benefit from using lateral wedge insoles for patients with knee osteoarthritis. However, potential benefits for certain individuals cannot be completely ruled out.

Supplementary References

1 Yang, X. *et al.* A large language model for electronic health records. *NPJ digital medicine* **5**, 194, doi:10.1038/s41746-022-00742-2 (2022).

2 Kanjee, Z., Crowe, B. & Rodman, A. Accuracy of a Generative Artificial Intelligence Model in a Complex Diagnostic Challenge. *JAMA* **330**, 78-80, doi:10.1001/jama.2023.8288 (2023).

3 Singhal, K. *et al.* Large language models encode clinical knowledge. *Nature* **620**, 172-180, doi:10.1038/s41586-023-06291-2 (2023).

4 Rahsepar, A. A. *et al.* How AI Responds to Common Lung Cancer Questions: ChatGPT vs Google Bard. *Radiology* **307**, e230922, doi:10.1148/radiol.230922 (2023).

5 Bernstein, I. A. *et al.* Comparison of Ophthalmologist and Large Language Model Chatbot Responses to Online Patient Eye Care Questions. *JAMA network open* **6**, e2330320, doi:10.1001/jamanetworkopen.2023.30320 (2023).

6 Lim, Z. W. *et al.* Benchmarking large language models' performances for myopia care: a comparative analysis of ChatGPT-3.5, ChatGPT-4.0, and Google Bard. *EBioMedicine* **95**, 104770, doi:10.1016/j.ebiom.2023.104770 (2023).

7 Yeo, Y. H. *et al.* Assessing the performance of ChatGPT in answering questions regarding cirrhosis and hepatocellular carcinoma. *Clinical and molecular hepatology* **29**, 721-732, doi:10.3350/cmh.2023.0089 (2023).

8 Wilhelm, T. I., Roos, J. & Kaczmarczyk, R. Large Language Models for Therapy Recommendations Across 3 Clinical Specialties: Comparative Study. *Journal of medical Internet research* **25**, e49324, doi:10.2196/49324 (2023).

9 Walker, H. L. *et al.* Reliability of Medical Information Provided by ChatGPT: Assessment Against Clinical Guidelines and Patient Information Quality Instrument. *Journal of medical Internet research* **25**, e47479, doi:10.2196/47479 (2023).

10 Koga, S., Martin, N. B. & Dickson, D. W. Evaluating the performance of large language models: ChatGPT and Google Bard in generating differential diagnoses in clinicopathological conferences of neurodegenerative disorders. *Brain Pathol*, e13207, doi:10.1111/bpa.13207 (2023).

11 Bhayana, R., Krishna, S. & Bleakney, R. R. Performance of ChatGPT on a Radiology Board-style Examination: Insights into Current Strengths and Limitations. *Radiology* **307**, e230582, doi:10.1148/radiol.230582 (2023).

12 Hirosawa, T., Mizuta, K., Harada, Y. & Shimizu, T. Comparative Evaluation of Diagnostic Accuracy Between Google Bard and Physicians. *Am J Med* **136**, 1119-1123.e1118, doi:10.1016/j.amjmed.2023.08.003 (2023).

13 Pushpanathan, K. *et al.* Popular large language model chatbots' accuracy, comprehensiveness, and self-awareness in answering ocular symptom queries. *iScience* **26**, 108163, doi:10.1016/j.isci.2023.108163 (2023).

14 H, F. *et al.* Comparison of Diagnostic and Triage Accuracy of Ada Health and WebMD Symptom Checkers, ChatGPT, and Physicians for Patients in an Emergency Department: Clinical Data Analysis Study. *JMIR mHealth and uHealth* **11**, doi:10.2196/49995 (2023).

15 Suárez, A. *et al.* Unveiling the ChatGPT phenomenon: Evaluating the consistency and accuracy of endodontic question answers. *Int Endod J*, doi:10.1111/iej.13985 (2023).

16 Ali, R. *et al.* Performance of ChatGPT and GPT-4 on Neurosurgery Written Board Examinations. *Neurosurgery* **93**, 1353-1365, doi:10.1227/neu.0000000000002632 (2023).

17 Hermann, C. E. *et al.* Let's chat about cervical cancer: Assessing the accuracy of ChatGPT responses to cervical cancer questions. *Gynecol Oncol* **179**, 164-168, doi:10.1016/j.ygyno.2023.11.008 (2023).

18 Cai, L. Z. *et al.* Performance of Generative Large Language Models on Ophthalmology Board-Style Questions. *American journal of ophthalmology* **254**, 141-149, doi:10.1016/j.ajo.2023.05.024 (2023).

19 Antaki, F. *et al.* Capabilities of GPT-4 in ophthalmology: an analysis of model entropy and progress towards human-level medical question answering. *Br J Ophthalmol*, bjo-2023-324438, doi:10.1136/bjo-2023-324438 (2023).

20 Fowler, T., Pullen, S. & Birkett, L. Performance of ChatGPT and Bard on the official part 1 FRCOphth practice questions. *Br J Ophthalmol*, bjo-2023-324091, doi:10.1136/bjo-2023-324091 (2023).

21 Passby, L., Jenko, N. & Wernham, A. Performance of ChatGPT on dermatology Specialty Certificate Examination multiple choice questions. *Clin Exp Dermatol*, llad197, doi:10.1093/ced/llad197 (2023).

22 Krusche, M., Callhoff, J., Knitza, J. & Ruffer, N. Diagnostic accuracy of a large language model in rheumatology: comparison of physician and ChatGPT-4. *Rheumatology international*, doi:10.1007/s00296-023-05464-6 (2023).

23 Hu, X. *et al.* What can GPT-4 do for Diagnosing Rare Eye Diseases? A Pilot Study. *Ophthalmol Ther* **12**, 3395-3402, doi:10.1007/s40123-023-00789-8 (2023).

24 Gencer, A. & Aydin, S. Can ChatGPT pass the thoracic surgery exam? *Am J Med Sci* **366**, 291-295, doi:10.1016/j.amjms.2023.08.001 (2023).

25 Smith, J., Choi, P. M. & Buntine, P. Will code one day run a code? Performance of language models on ACEM primary examinations and implications. *Emerg Med Australas* **35**, 876-878, doi:10.1111/1742-6723.14280 (2023).

26 Toyama, Y. *et al.* Performance evaluation of ChatGPT, GPT-4, and Bard on the official board examination of the Japan Radiology Society. *Japanese journal of radiology*, doi:10.1007/s11604-023-01491-2 (2023).

27 Kleinig, O. *et al.* How to use large language models in ophthalmology: from prompt engineering to protecting confidentiality. *Eye (London, England)*, doi:10.1038/s41433-023-02772-w (2023).

28 Akinci D'Antonoli, T. *et al.* Large language models in radiology: fundamentals, applications, ethical considerations, risks, and future directions. *Diagnostic and interventional radiology (Ankara, Turkey)*, doi:10.4274/dir.2023.232417 (2023).

29 Wei, W. I. *et al.* Extracting symptoms from free-text responses using ChatGPT among COVID-19 cases in Hong Kong. *Clin Microbiol Infect*, S1198-1743X(1123)00540-00542, doi:10.1016/j.cmi.2023.11.002 (2023).

30 Fink, M. A. *et al.* Potential of ChatGPT and GPT-4 for Data Mining of Free-Text CT Reports on Lung Cancer. *Radiology* **308**, e231362, doi:10.1148/radiol.231362 (2023).

31 Meskó, B. Prompt Engineering as an Important Emerging Skill for Medical Professionals: Tutorial. *Journal of medical Internet research* **25**, e50638, doi:10.2196/50638 (2023).
